# Receptor allostery promotes context-specific Sonic Hedgehog signaling during embryonic development

**DOI:** 10.1038/s41467-026-75918-5

**Published:** 2026-07-24

**Authors:** Miriam E. Dillard, Christina A. Daly, Rojalin Pradhan, Shariq S. Ansari, Samantha J. Hack, William C. Wright, Daniel P. Stewart, Mohamed A. Ghonim, Yan Zhang, Robin Canac, Ivan P. Moskowitz, Shondra M. Pruett-Miller, Jeffrey Steinberg, Yong-Dong Wang, April D. Sykes, Motomi Mori, Christopher C. Hom, Taosheng Chen, Paul G. Thomas, James P. Bridges, Stacey K. Ogden

**Affiliations:** 1 Department of Cell and Molecular Biology, St. Jude Children’s Research Hospital, Memphis, TN USA; 2 Department of Chemical Biology and Therapeutics, St. Jude Children’s Research Hospital, Memphis, TN USA; 3 Department of Host Microbe Interactions, St. Jude Children’s Research Hospital, Memphis, TN USA; 4 Department of Pediatrics, The University of Chicago, Chicago, IL USA; 5 Center for Advanced Genome Engineering, St. Jude Children’s Research Hospital, Memphis, TN USA; 6 Center for In Vivo Imaging and Therapy, St. Jude Children’s Research Hospital, Memphis, TN USA; 7 Department of Biostatistics, St. Jude Children’s Research Hospital, Memphis, TN USA; 8 Department of Medicine, Division of Pulmonary, Critical Care and Sleep Medicine, National Jewish Health, Denver, CO USA; 9 Department of Medicine, Division of Pulmonary Sciences and Critical Care, University of Colorado Anschutz Medical Campus, Aurora, CO USA

**Keywords:** Morphogen signalling, Developmental biology, Membrane proteins

## Abstract

Sonic Hedgehog (SHH) signaling functions in temporal- and context-dependent manners to pattern diverse tissues during embryogenesis. The signal transducer Smoothened (SMO) is induced by sterols, oxysterols, and arachidonic acid (AA) through binding pockets in its extracellular cysteine-rich domain (CRD) and 7-transmembrane (7TM) bundle. In vitro analyses suggest SMO signaling is allosterically enhanced by combinatorial ligand binding to these pockets, but in vivo evidence of SMO allostery is lacking. Herein, we map an AA binding pocket at the top of the 7TM bundle and show that its disruption attenuates SHH- and sterol-stimulated SMO induction. A knock-in mouse model of compromised AA binding reveals that homozygous mutant mice are cyanotic, exhibit perinatal lethality, and display congenital heart disease. Surviving mutants demonstrate pulmonary maldevelopment and fail to thrive. Neurodevelopment is unaltered in these mice, suggesting that context-specific allosteric regulation of SMO signaling allows for precise tuning of pathway activity during cardiopulmonary development.

## Abstract (web summary)

Here, they identify a binding site on the Sonic Hedgehog signaling protein Smoothened for the allosteric regulator arachidonic acid (AA). A mutation that disrupts AA binding to this pocket compromises cardiopulmonary development.

## Introduction

Embryonic development relies on precise spatial and temporal coordination of cell behaviors that facilitate tissue morphogenesis. The Sonic Hedgehog (SHH) signaling pathway contributes to tissue organization by providing signal-responding cells with positional information and cell fate guidance cues^1,2^. Through this activity, SHH instructs body asymmetry and promotes development of diverse organ systems including the nervous system, heart, lungs, digestive tract, and musculoskeletal system^3–6^. Consistent with its role as a master regulator of tissue patterning and homeostasis, dysregulation of SHH signaling leads to developmental disorders such as holoprosencephaly, congenital heart disease (CHD), and lung hypoplasia, and aberrant signaling can drive tumor formation in the cerebellum and skin^1,4,6,7^.

In most vertebrate cell types, the SHH signaling response is coordinated through the primary cilium (PC), a specialized sensory organelle that extends from the basal body and facilitates signaling by several members of the G protein-coupled receptor (GPCR) superfamily^8,9^. In the absence of ligand, the SHH receptor Patched (PTCH) localizes to the ciliary base and depletes sterols from ciliary membranes^10–12^. Low sterol content prevents PC membrane accumulation of the GPCR Smoothened (SMO), which functions as the signal transducer for the SHH pathway. In this off state, the SHH transcriptional effectors GLI2 and GLI3 are phosphorylated by cAMP-dependent protein kinase (PKA) in the PC, which leads to their partial proteolysis into transcriptional repressors^13^. Pathway activation occurs upon SHH binding to PTCH, which triggers its internalization and degradation^14^. Removal of PTCH from ciliary and plasma membranes eliminates its sterol-depleting activity, allowing for localized accumulation of ciliary sterols that bind SMO to stimulate its PC retention and downstream signaling^10–12^.

Once active, SMO can signal through two distinct effector routes. The first is the canonical signal, which requires SMO accumulation in the PC. Ciliary SMO signaling blocks phosphorylation and partial proteolysis of GLI2/3 transcription factors, allowing for their activation to induce downstream target genes like *Gli1*^13,15,16^. The second SMO signal, referred to as the noncanonical effector route, can occur from outside the PC. This signal leads to activation of Gαi heterotrimeric G proteins, which signal to activate transcription-independent SHH responses that influence lipid metabolism, calcium release, and cytoskeletal regulation^15,17–20^. The canonical signal that controls GLI is thought to function independently of the noncanonical SMO-Gαi signaling arm^15^. However, we have reported that, in some contexts, noncanonical signaling through Gαi can enhance canonical pathway activity by augmenting ligand-stimulated SMO ciliary accumulation^18,21^.

Current models suggest that accumulation and activation of SMO in the PC result from direct sterol binding to sites in its extracellular cysteine-rich domain (CRD) and deep in its 7-transmembrane (7TM) bundle^10,22–24^. However, lipids including the oxysterols 20(S)-hydroxycholesterol (OHC), 7β,27-dihydroxycholesterol (DHC), and 24(S),25-epoxycholesterol (EpCHO) have also been reported to bind to sites in the CRD, 7TM bundle, and/or intracellular (IC) loops to promote SMO activation^23,25–29^. Although the identity of the natural ligand and location of its primary orthosteric binding pocket remain topics of debate, most reports agree that cholesterol or oxysterol binding to the SMO CRD is crucial for SHH-stimulated signal induction^10,22,23,25,27,28,30^. Accordingly, mutation of the residues that coordinate sterol binding disrupts SHH-mediated SMO activation^10,23,25–27,31^.

**Fig. 1:**
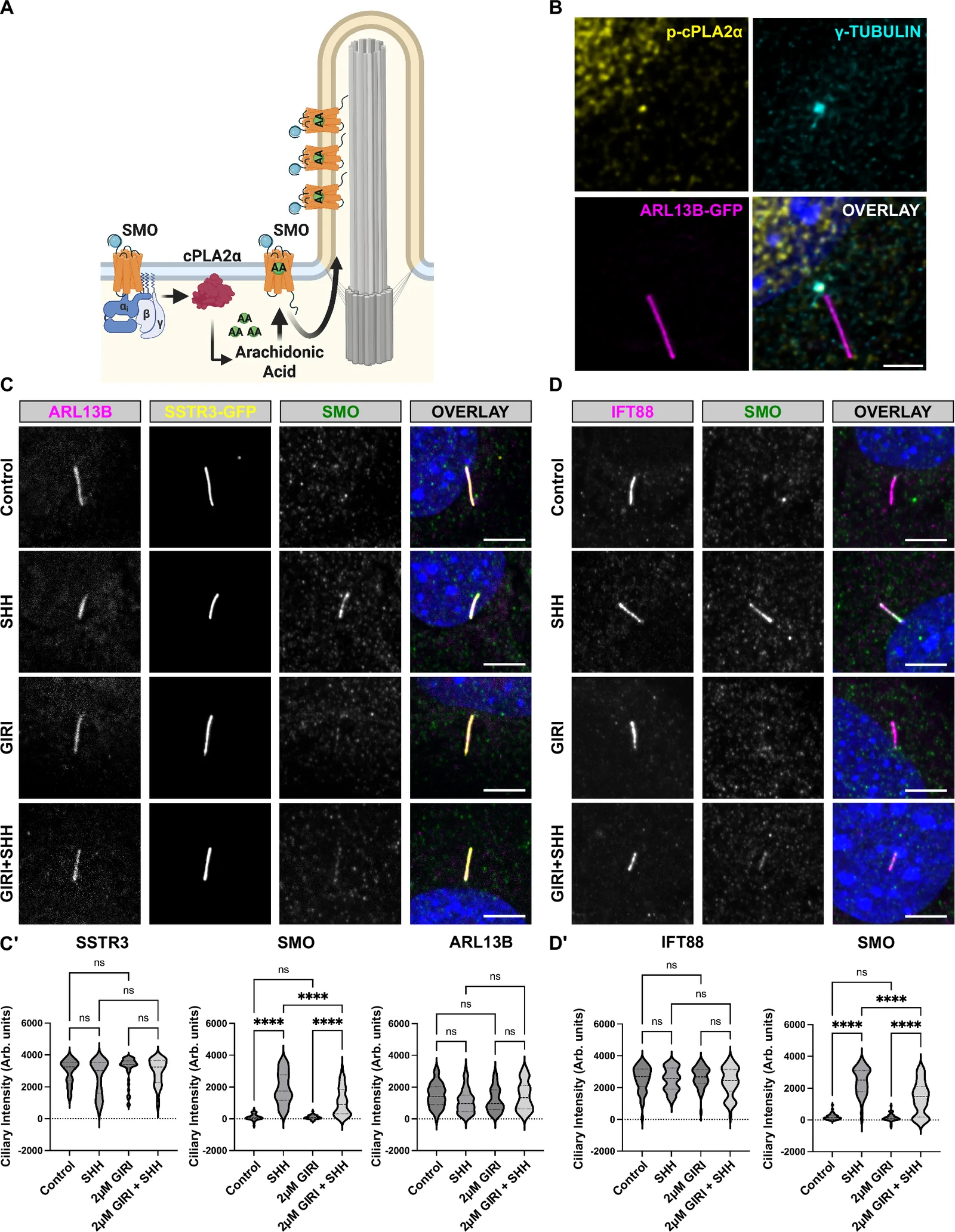
cPLA2α contributions to ciliary trafficking are SMO specific. **A** SMO signal induction by a cysteine-rich domain (CRD)-binding agonist (blue circle) activates Gαiβγ to stimulate cPLA2α production of arachidonic acid (AA). AA is proposed to interact with the 7TM domain of active SMO to enhance its primary cilium (PC) enrichment in SHH-stimulated cells. Created in BioRender. Daly, C. (2026) https://BioRender.com/aqxuhk9. **B** A fraction of endogenous phospho-cPLA2α (yellow) localizes to the base of an NIH-3T3 cell PC (marked by γ-tubulin, cyan). ARL13B-GFP (magenta) marks the ciliary axoneme. A representative image of *n* = 4 is shown. Scale bar = 2 μm. **C**-**C’** NIH-3T3 cells expressing SSTR3-GFP (yellow) were treated with vehicle (DMSO), 2 µM GIRI, and control or SHH-conditioned media as indicated for 18 hours. Endogenous ARL13B is magenta, endogenous SMO is green, and DAPI is blue. Scale bar = 5 μm. **C’** Quantification of ciliary signal intensity. The experiment was performed twice with a total of 50 cilia imaged per condition. **D**-**D’** NIH-3T3 cells were treated with control or SHH-conditioned media in the presence of 2 µM GIRI or vehicle. IFT88 is shown in magenta, SMO is green, and DAPI (blue) marks nuclei. Scale bar = 5 μm. **D’** Quantification of ciliary signal intensity. The experiment was performed twice with a total of 50 cilia imaged per condition. Data are represented as mean ± SD. Significance was determined by one-way ANOVA and is indicated as follows: *<0.05, **<0.01, ***<0.001, ****<0.0001, and ns, *p* > 0.05. Source data are provided as a Source Data file.

Notably, many GPCRs have secondary ligand binding pockets that are spatially distinct from the orthosteric site. These pockets are referred to as allosteric sites and, when occupied by allosteric ligands, allow for enhancement or suppression of orthosteric ligand-induced signaling activity^32^. Consistent with this biology, we previously identified arachidonic acid (AA) as a feed-forward enhancer of SHH- or oxysterol-activated SMO signaling^18^. The feed-forward signal is initiated by SMO signaling through Gαi to release Gβγ. Free Gβγ directly stimulates cytosolic phospholipase A2α (cPLA2α) at the ciliary base to cleave arachidonate-containing phospholipids for AA production^18^. Localized synthesis of AA in response to pathway induction enhances SMO signaling through both indirect and direct mechanisms. Indirect effects on pathway activity result from metabolism of AA into prostaglandin E2, which activates ciliary E-type prostanoid receptor 4 (EP4) to ensure PC length stability^21^. Direct effects on SMO signaling occur through AA targeting the 7TM domain to augment ligand-stimulated ciliary accumulation for enhanced communication with downstream GLI transcriptional effectors (Fig. 1A)^18^. This activity is thought to occur through direct binding of AA to the SMO 7TM domain because AA can displace the 7TM-binding inverse agonist cyclopamine in cell-based assays. Moreover, blocking SHH-stimulated AA production through inhibition of cPLA2α reduces the amplitude of the SMO signal response, consistent with AA acting as a feed-forward enhancer of canonical SMO signaling^18,21^.

Multiple reports have provided structural evidence and in vitro functional data supporting that SMO allostery enhances signal output^10,18,25,27,28^. However, in vivo evidence for allosteric regulation of SMO signaling has remained elusive. Herein, we interrogate SMO allostery in vitro and in vivo to define the precise contribution of AA to SMO signaling activity during tissue morphogenesis. We show that AA enhances SMO ciliary enrichment induced by CRD- and TM-binding oxysterols and cholesterol, map a specific AA binding site at the top of the 7TM bundle, and identify an essential anchoring residue for AA in extracellular loop 2 (EC2). A single amino acid change at this site specifically attenuates AA binding and blunts SMO ciliary localization and signaling without compromising the structural integrity of the binding pocket. We introduce a novel knock-in mouse model for reduced AA binding and show that homozygous mutation of the AA anchoring residue leads to high perinatal lethality. Neurodevelopment occurs normally in these animals, but cardiopulmonary development is disrupted. As such, we propose that AA allosterically enhances ligand-activated SMO signaling and that this functionality contributes to in vivo pathway activity during embryonic development in an organ-specific manner.

## Results

### AA specifically enhances SHH-stimulated SMO ciliary enrichment

The active phosphorylated form of cPLA2α is detected at the base of ARL13B-marked primary cilia of SHH-responsive NIH-3T3 cells, where it overlaps with the centrosome marker γ-tubulin (Fig. 1B)^18^. The presence of cPLA2α at the PC base raises the possibility that localized production of AA might impact ciliary enrichment of proteins other than SMO. To test this hypothesis, we selected three ciliary-resident proteins, the GPCR Somatostatin Receptor 3 (SSTR3), the GTPase ARL13B, and the intraflagellar transport protein IFT88, and evaluated whether cPLA2α inhibition would disrupt their PC localization. We expressed a green fluorescent protein (GFP)-tagged version of SSTR3 in NIH-3T3 cells and then monitored localization of SSTR3-GFP, endogenous ARL13B, and endogenous IFT88 following treatment with the cPLA2α-specific inhibitor Giripladib (GIRI)^18,21,33^. As a control for cPLA2α inhibition, we tracked SHH-stimulated PC accumulation of endogenous SMO in vehicle and GIRI-treated cells. Consistent with our previous reports that cPLA2α inhibition prevents SHH-stimulated AA production, GIRI treatment significantly reduced SHH-induced SMO ciliary enrichment (Fig. 1C- D’)^18,21^. Conversely, PC localization of ARL13B, SSTR3-GFP, and IFT88 was unaffected by GIRI in both control and SHH-stimulated cells (Fig. 1C- D’). Taken together with previously published results that GIRI does not alter SHH-regulated dynamic ciliary trafficking of the GPCR GPR161^21^, these results suggest that cPLA2α-mediated AA production is unlikely to be a general modulator of PC protein entry and that its effect is likely specific to SMO.

### AA enhances sterol-stimulated SMO ciliary enrichment for downstream signaling

SMO signaling can be induced by direct binding of cholesterol or oxysterols to distinct ligand-binding pockets in its CRD, 7TM, and IC loop domains. Previous reports show that active SMO conformations are induced by CRD-binding 7β,27-DHC, CRD and 7TM-binding cholesterol (CHO), and 24(S),25-EpCHO, which promotes SMO activation through the CRD, 7TM bundle, and IC loops (Supplementary Fig. 1A)^23–25,28,30,34,35^. AA synergizes with another CRD-binding agonist, 20(S)-OHC, to augment SMO ciliary accumulation for enhanced downstream signaling^18,31^. To probe the effect of cPLA2α-produced AA on SMO PC entry induced by 7β,27-DHC, 24(S),25-EpCHO, or CHO:MβCD^36^, hereafter collectively referred to as “sterols”, we treated NIH-3T3 cells with GIRI in the absence or presence of SHH or each sterol and then quantified SMO ciliary signal intensity.

**Fig. 2:**
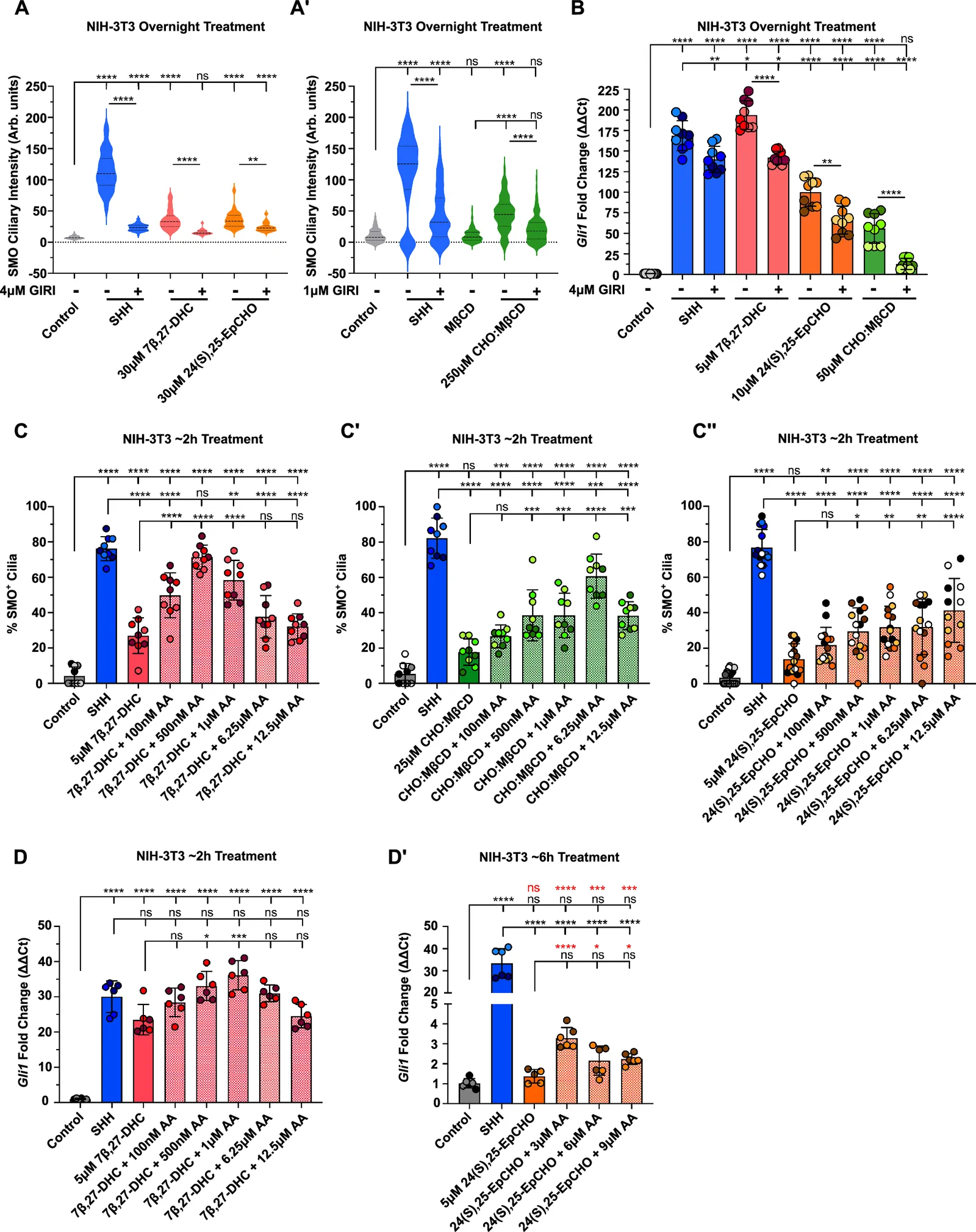
AA enhances sterol-mediated SMO activation. **A**-**A**’ Quantification of SMO ciliary signal intensity in NIH-3T3 cells. Cells were treated with vehicle or the indicated agonists following pretreatment with the indicated concentrations of GIRI or vehicle. Sterol-treated cells were compared with cells treated with control or SHH-N conditioned media. Lower concentrations of GIRI were used when combined with CHO:MβCD to prevent cells sloughing off the dish (**A’**). Experiments were repeated twice with a total of 43-199 cilia imaged per condition. **B** NIH-3T3 cells were incubated with vehicle or the indicated agonists following vehicle or 4 µM GIRI pretreatment. ∆∆Ct values of *Gli1* were calculated relative to control and normalized to the *Btf3* reference gene ~18 hours post agonist/GIRI treatment. The graph represents pooled data from three independent experiments done in triplicate. NIH-3T3 cells were treated with control or SHH-conditioned media or 5 µM 7β,27-DHC (**C**), 25 µM CHO: MβCD (**C’**), or 5 µM 24(S),25-EpCHO (**C”**) ± arachidonic acid (AA). Endogenous SMO was stained for immunofluorescence analysis, and percent SMO^+^ cilia was quantified over three independent experiments totaling 95-146 (**C**), 107-150 (**C’**), or 167-203 (**C”**) cilia per condition. NIH-3T3 cells were treated with control or SHH-conditioned media, 5 µM 7β,27-DHC (**D**), or 5 µM 24(S),25-EpCHO (**D’**) plus increasing concentrations of AA as indicated. ∆∆Ct values were calculated as in (**B**) for two experiments with three technical replicates per condition per experiment. Statistical significance was calculated for the full data set (black indicators) and for the dataset with SHH values omitted (**D’**, red indicators). Significance was determined by two-way ANOVA and indicated as follows: *<0.05, **<0.01, ***<0.001, ****<0.0001, and ns, *p* > 0.05. For **B**–**D’**, individual data points are shaded to indicate experimental replicates. All bar graphs are plotted as mean ± S.D. Source data are provided as a Source Data file.

SMO ciliary accumulation was induced by overnight treatment with each sterol, albeit to significantly lower levels than what occurred following SHH stimulation (Fig. 2A–A’, Supplementary Fig. 1B, C). These results are consistent with previously published reports and likely indicate that additional pathway responses that are activated by SHH are not fully recapitulated by these direct SMO sterol agonists^22,28^. Notably, the ability of SHH or activating sterols to induce SMO ciliary accumulation was significantly blunted by GIRI treatment (Fig. 2A–A’, Supplementary Fig. 1B, C). This suggests that AA production downstream of active SMO augments its ciliary entry independent of which upstream agonist is provided.

Consistent with our previous report that cPLA2α inhibition can shorten cilia of SHH-stimulated cells, average PC length trended down slightly in cells exposed to SHH plus GIRI (Supplementary Fig. 1B’, C’, blue)^21^. Curiously, combining 7β,27-DHC with GIRI or treating cells with CHO:MβCD in either the absence or presence of GIRI significantly increased average PC length. We do not know the reason for these increases. However, we do not attribute the CHO:MβCD-induced changes to differences in AA availability because length increase was unaffected by GIRI treatment (Supplementary Fig. 1C–C’, green). We hypothesize the effect may result from CHO supplementation because its depletion induces the opposite effect of compromising ciliogenesis and shortening PC length in zebrafish embryos and cultured IMCD3 cells^37^.

We next performed qPCR analyses to test whether GIRI-induced attenuation of SHH or sterol-stimulated SMO PC retention altered induction of the endogenous *Gli1* target gene. Owing to qPCR being a more sensitive assay than immunofluorescence analysis of SMO PC enrichment, we were able to measure downstream pathway activation at lower doses of agonist treatment than were used for SMO ciliary signal intensity studies. The SHH and 7β,27-DHC-stimulated *Gli1* transcriptional responses were highly robust, inducing activity ~175-fold over baseline following overnight treatment (Fig. 2B, blue and red). *Gli1* activation through 24(S),25-EpCHO and CHO:MβCD stimulation was also significant, but less pronounced than what occurred in response to SHH or 7β,27-DHC (Fig. 2B, orange and green). For all conditions, *Gli1* induction was significantly attenuated by GIRI treatment, supporting that the downstream transcriptional response is augmented by cPLA2α activity (Fig. 2B).

To directly test if the cPLA2α-mediated effects likely occurred through its product AA, we evaluated whether addition of supplemental AA in the presence of direct sterol agonists would enhance SMO ciliary localization or signaling. We first tested whether treatment of cells with supplemental AA in the absence of sterol would influence *Gli1* expression or impact cell viability when provided at high doses. Consistent with our previous report^18^, AA failed to induce *Gli1* expression at any dose when provided in the absence of a SMO sterol agonist (Supplementary Fig. 1D). Importantly, AA supplementation did not adversely impact cell viability, even after overnight treatment at high doses, as supported by stable expression of the reference gene *Btf3* (Supplementary Fig. 1D’).

We next assessed AA impact on SMO ciliary localization by treating NIH-3T3 cells with sterols alone or in the presence of increasing concentrations of supplemental AA. Because overnight sterol agonist treatment resulted in pronounced SMO activity increases that might mask allosteric signal enhancement, we chose to test for AA-stimulated feed-forward augmentation of SMO ciliary accumulation or target gene induction at lower agonist doses and shorter time points ranging from 2-6 hours. Ciliary occupancy was detected within ~2 hours of sterol stimulation, with each sterol inducing SMO PC occupancy in ~20% of cells scored (Fig. 2C–C”, Supplementary Fig. 1E). Sterol stimulation induced SMO ciliary occupancy less effectively than SHH, which showed ~80% ciliary occupancy by 2 hours post-treatment (Fig. 2C–C” blue, Supplementary Fig. 1E). Addition of increasing concentrations of AA in the presence of 7β,27-DHC augmented SMO ciliary enrichment to a level equivalent to SHH at maximal combined effectiveness and then reverted to the baseline level of 7β,27-DHC-induced ciliary occupancy at higher AA doses (Fig. 2C, Supplementary Fig. 1E). A similar bell-shaped response curve occurred for CHO:MβCD at 2 hours, again with the maximal effective CHO:MβCD + AA concentration triggering SMO ciliary occupancy near to what was observed with SHH (Fig. 2C’, Supplementary Fig. 1E). These bell-shaped curves suggest a mixed agonist/antagonist or feedback response by SMO to the combined action of AA plus 7β,27-DHC or CHO:MβCD. Intriguingly, SMO ciliary localization induced by multi-site binding 24(S),25-EpCHO was also enhanced by AA co-treatment at 2 hours, but augmentation of ciliary occupancy was less pronounced, reaching ~50% of the SHH-induced PC occupancy level at the highest AA dose evaluated (Fig. 2C”, Supplementary Fig. 1E).

To determine whether AA enhancement of SMO PC enrichment correlated with transcriptional changes, we tested the effect of AA supplementation on *Gli1* target gene induction. We observed a strong *Gli1* transcriptional response after only ~2 hours of 7β,27-DHC treatment (Fig. 2D). The transcriptional response to combining AA with 7β,27-DHC paralleled what was observed for SMO ciliary localization; AA enhanced transcriptional output along a similar bell-shaped curve (Fig. 2D). Despite its ability to induce SMO ciliary entry within 2 hours of treatment, 24(S),25-EpCHO-stimulated SMO signaling to GLI effectors was modest, showing nonsignificant *Gli1* induction by 6 hours post-treatment (Fig. 2D’). This result is consistent with a previous report that 24(S),25-EpCHO is a weaker SMO agonist than 7β,27-DHC^28^. However, combining AA with 24(S),25-EpCHO enhanced *Gli1* induction over the sterol baseline (Fig. 2D’). Whereas AA enhancement of 24(S),25-EpCHO-stimulated *Gli1* induction was significant when the SHH response was not included in statistical analyses, enhancement failed to reach significance when the SHH-stimulated condition was compared (Fig. 2D’, red vs. black significance indicators). We speculate that the differential effects of AA on SMO PC enrichment and *Gli1* induction by 7β,27-DHC and 24(S),25-EpCHO may stem from the ability of 24(S),25-EpCHO to occupy at least three binding pockets on SMO, which may lessen its responsiveness to AA allostery (Supplementary Fig. 1A).

We were unable to gauge the effect of AA on CHO:MβCD-stimulated *Gli1* induction because combining AA with CHO:MβCD blocked *Gli1* induction at every AA dose tested (Supplementary Fig. 1F). This is likely a technical complication resulting from combining AA with MβCD-loaded CHO and not an indication that AA blocks CHO-mediated SMO signaling to GLI. AA enriches in lipid rafts, which are disrupted by MβCD treatment^38,39^. Moreover, MβCD can mobilize fatty acids into and out of cell membranes at lower concentrations than are required for CHO extraction or addition^40^. This suggests that AA, which does not induce a *Gli1* transcriptional response on its own (Supplementary Fig. 1D), could be delivered to cells by the MβCD carrier without the inclusion of CHO. Nevertheless, the positive effects of AA on sterol-stimulated SMO PC localization, along with its ability to augment 7β,27-DHC and 24(S),25-EpCHO-mediated transcriptional activation, suggest that AA may function as an allosteric enhancer of sterol-activated SMO signaling. The difference in responsiveness of 7β,27-DHC, CHO:MβCD and 24(S),25-EpCHO-stimulated SMO to AA may indicate that the sterol binding pocket(s) may influence the potency of or requirement for AA-mediated allosteric enhancement.

### AA is predicted to bind at the top of the SMO 7TM bundle

**Fig. 3:**
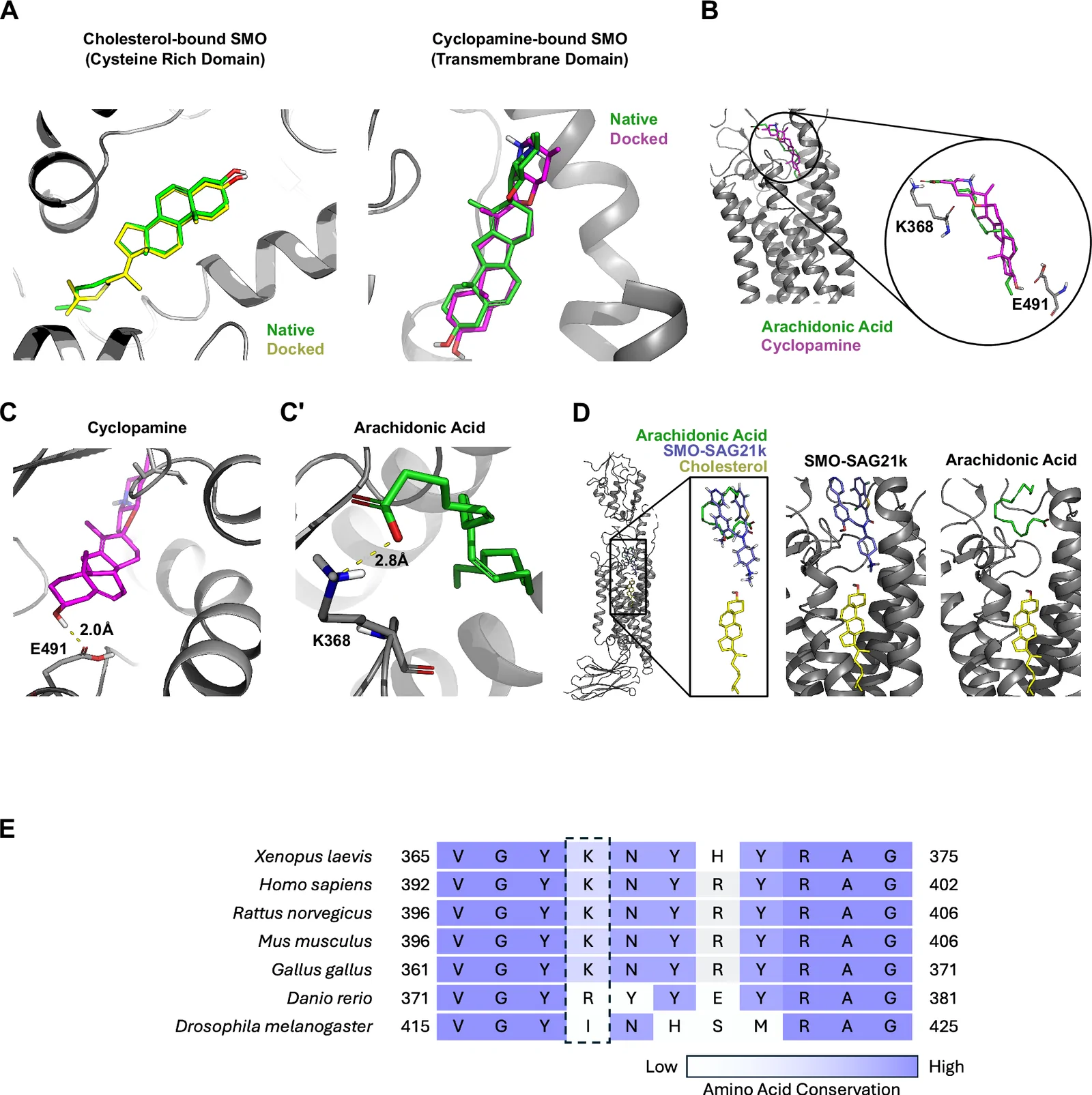
In silico docking predicts an AA binding site on SMO. **A** In silico docking (yellow) of Cholesterol (CHO) recapitulates the native (green) binding pose observed in the crystal structure of cholesterol bound to the human SMO extracellular cysteine-rich domain (CRD) (PDB ID: 5L7D). Docking of cyclopamine (magenta) to the transmembrane domain (7TM) recapitulates the binding pose observed in the crystal structure (green) of cyclopamine-bound *Xenopus* SMO (PDB ID: 6D32). **B** In silico docking of cyclopamine (magenta) and arachidonic acid (AA, green) onto *Xenopus* SMO using Maestro software predicts cyclopamine and AA bind through an overlapping pocket near the top of the 7TM of SMO. **C** Cyclopamine is anchored by *Xenopus* SMO E491 (E522 in murine SMO) in transmembrane helix 7. **C’** AA is predicted to form a salt bridge with *Xenopus* SMO K368 (murine SMO K399) in extracellular loop 2 (EC2). **D** In silico docking predicts that the AA (green) binding pocket overlaps with the SAG (blue) pocket, but not with the deep 7TM CHO (yellow) binding pocket of the murine SMO structure (PDB ID: 6O3C). **E** Alignment of EC2 sequence from SMO proteins. Darker shading indicates higher conservation. The predicted AA anchoring residue is indicated by the dashed box.

To identify a candidate binding site for AA on SMO that might clarify the differential sterol responsiveness to AA supplementation, molecular docking experiments were performed. We used Maestro software (Schrödinger Release 2019-3) with receptor grids based on published crystal structures of sterol- or cyclopamine-bound *Xenopus* and human SMO proteins^27,41,42^. Grids were validated by redocking cholesterol onto the CRD and cyclopamine onto the upper TM/EC loop pocket (Fig. 3A). The high degree of overlap between native and docked binding molecules supported the feasibility of using in silico docking to predict how AA might bind SMO (Fig. 3A green vs. yellow or magenta). Experimental in silico docking predicted AA binding near the top of the SMO 7TM domain in a pocket that significantly overlapped with the pocket targeted by cyclopamine and SAG (Fig. 3B–D)^34^. The high degree of predicted overlap between AA and cyclopamine is consistent with our previously published observation that AA can displace Bodipy-cyclopamine from SMO in cell-based binding assays^18^. Notably, the predicted binding pocket of AA abuts the TM binding pocket of 24(S),25-EpCHO^26^, providing a potential explanation for why 24(S),25-EpCHO-stimulated SMO is less responsive to AA than SMO activated by other sterols (Fig. 2).

Whereas cyclopamine and AA were predicted to occupy a similar space in the upper 7TM/EC pocket, they were predicted to anchor through distinct residues in the EC loops and 7TM helices (Fig. 3B-C’). Cyclopamine anchors through a hydroxyl group that forms a hydrogen bond with the side chain of E491 in TM helix 7 (Fig. 3C). In its most favorable predicted pose, the hydroxyl group of AA is deprotonated to form an O^-^ anion that facilitates a salt bridge with the α-amino group of K368 in EC loop 2 (Fig. 3C’). Accordingly, molecular dynamics simulation of AA-bound *Xenopus* SMO predicts that K368 forms the strongest bond to AA over the course of the 20-nanosecond simulation and that it makes the most consistent contact with AA compared to all other residues surrounding the binding site (Supplementary Fig. 2A, B, Supplementary Movie 1). The predicted topology of AA association with K368 of *Xenopus* SMO positions the fatty acid in a binding pocket that is predicted to be completely offset from the deep 7TM CHO-binding pocket. Accordingly, simultaneous docking of AA and CHO to murine SMO suggests both molecules may bind simultaneously (Fig. 3D). Curiously, a different binding posture of AA is predicted in the co-occupied receptor compared to that predicted in the absence of CHO (Fig. 3B, C’ vs. D). We speculate that the difference in the predicted AA pose between the murine and *Xenopus* structures in the static CHO-bound crystal structure is due to AA’s high number of rotatable bonds conferring a high degree of flexibility. Unlike more rigid compounds like CHO, AA can adopt various low-energy conformations that could be influenced by the local protein conformational environment. Consistent with this hypothesis, the molecular dynamics simulation suggests that AA binds the pocket dynamically and then quickly adopts a linear pose that is stable and maintains prominent contact with the K368 residue (Supplementary Movie 1). Importantly, alignment of SMO protein sequence across species reveals conservation of the predicted AA anchoring lysine and its flanking sequence, particularly across vertebrate SMO proteins (Fig. 3E). Overlay of published *Xenopus*, human, and mouse SMO structures suggests similar topology of all SMO proteins in this region, consistent with the predicted binding pocket and anchoring residue being physiologically relevant (Supplementary Fig. 2C)^27,41,43^. Accordingly, *Xenopus* SMO K368 corresponds to K395 of human SMO, which is a key coordinating residue for binding of several small-molecule SMO inhibitors^25,44^.

### A lysine anchor stabilizes AA binding to SMO

To directly evaluate whether the lysine anchor was essential for AA binding to SMO, we used a previously described binding assay in which click chemistry-generated AA beads are used to capture SMO-YFP from cell lysates^18^. We began to characterize SMO-AA binding by testing the ability of SMO to bind AA in the presence of CRD-binding 7β,27-DHC or multi-site binding 24(S),25-EpCHO. AA beads were incubated with lysates from HEK293T cells expressing murine SMO-YFP proteins in the presence of vehicle, sterol, free AA, or the negative control *N-*stearoyldopamine^18^. We limited our evaluation of SMO ligand co-occupancy to AA plus 7β,27-DHC and 24(S),25-EpCHO due to the above-mentioned technical limitations of combining AA with CHO:MβCD.

**Fig. 4:**
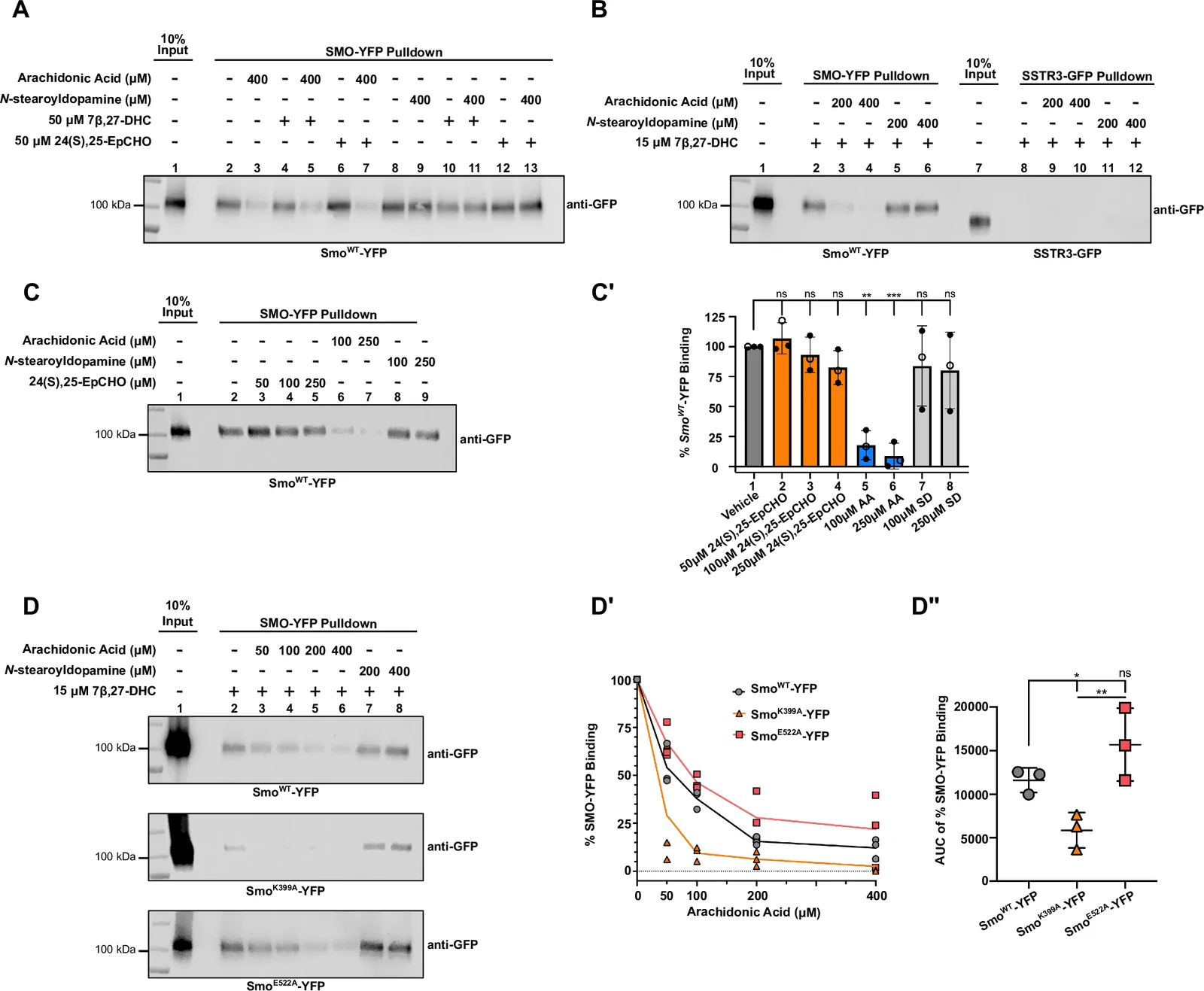
K399 stabilizes AA binding to murine SMO. **A** Arachidonic acid (AA) beads were incubated with lysates from HEK293T cells expressing SMO-YFP in the presence of vehicle or the indicated concentrations of 7β,27-DHC, 24(S),25-EpCHO, *N*-stearoyldopamine (negative control), and/or free AA. SMO is specifically displaced from beads only by free AA. **B**) AA beads were incubated with lysates from HEK293T cells expressing SMO-YFP or the ciliary GPCR SSTR3-GFP in the presence of free AA, *N*-stearoyldopamine, and/or 7β,27-DHC. SMO-YFP binds AA beads specifically; SSTR3-GFP does not bind. **C**-**C’** AA beads were incubated with lysates from HEK293T cells expressing SMO-YFP in the presence of vehicle or increasing concentrations of free AA, 24(S),25-EpCHO, or *N*-stearoyldopamine. SMO-YFP is specifically displaced only by free AA. **C’** Densitometry analysis of three individual experiments was performed to quantify percent SMO-YFP bead binding and all data were averaged. Results are plotted as mean ± S.D, with open circles corresponding to the blot in (**C**). **D**-**D**” AA beads were incubated in lysates from HEK293T cells expressing SMO^WT^-YFP, SMO^K399A^-YFP, or cyclopamine-binding deficient SMO^E522A^-YFP in the presence of 7β,27-DHC and increasing concentrations of free AA or *N*-stearoyldopamine. Densitometry analysis of three individual experiments was performed and quantified as percent SMO-YFP bead binding (**D'**). Area under the curve (AUC) was calculated and revealed a significant reduction in SMO^K399A^-YFP bead binding, compared to SMO^WT^-YFP and SMO^E522A^-YFP proteins (**D”**). Results are plotted as mean ± S.D. Significance was determined by one-way ANOVA (**C’**) and Post-hoc pairwise comparisons (**D”**). For all experiments, significance is indicated as follows: *<0.05, **<0.01, ***<0.001, ****<0.0001, and ns, *p* > 0.05. Source data are provided as a Source Data file.

Anti-GFP western blots of proteins eluted from the AA beads revealed capture of SMO-YFP by AA when provided alone or in the presence of either oxysterol (Fig. 4A, lanes 2, 4, 6, 8, 10, 12). SMO-YFP was competed off the AA beads by high concentrations of free AA, but not by the negative control *N*-stearoyldopamine, supporting specific association (Fig. 4A lanes 3, 5, 7 vs. 9, 11, 13). AA beads incubated in lysates from cells expressing the ciliary GPCR SSTR3-GFP, which localizes to the primary cilium in an AA-independent manner, failed to capture SSTR3 (Figs. 1C and 4B). This further supports SMO-AA binding specificity.

The 7TM binding pocket of multi-site binding 24(S),25-EpCHO and the predicted binding pocket of AA abut one another^26^, suggesting that, despite the ability of SMO-YFP to bind AA beads in the presence of 50 μM 24(S),25-EpCHO (Fig. 4A), the two lipids might compete for SMO binding when 24(S),25-EpCHO concentrations are increased. Thus, we evaluated SMO-AA bead binding against a broad 24(S),25-EpCHO dose curve. Lysates from SMO-YFP-expressing cells were preincubated with increasing concentrations of 24(S),25-EpCHO (50-250 μM) for 30 minutes prior to AA bead incubation. SMO-YFP capture on AA beads was unaltered across the 24(S),25-EpCHO dose curve, arguing against competitive binding between AA and the oxysterol (Fig. 4C–C’, lanes 2-5).

Having established the specificity of SMO^WT-^YFP binding to AA beads, we next sought to test the effect of mutation of the anchoring lysine on SMO-AA association. We introduced a lysine-to-alanine substitution into the murine SMO residue corresponding to K368 of Xenopus SMO (K399) and tested SMO^K399A^-YFP binding to AA beads. Beads were incubated with lysates from cells expressing WT, K399A, or cyclopamine-binding-deficient SMO-YFP^E522A^^35^. We reasoned that SMO^E522A^ would act as a control for a binding pocket mutation that is not predicted to impact AA anchoring (Fig. 3B). Because AA is proposed to bind sterol-activated SMO, and AA-mediated augmentation of 7β,27-DHC-stimulated SMO activity was more pronounced than what occurred with 24(S),25-EpCHO, 7β,27-DHC was included in these experimental binding reactions. All three SMO variants were captured on AA beads (Fig. 4D, lane 2). However, SMO^K399A^-YFP bound the AA beads with decreased affinity and was displaced by lower concentrations of free AA compared to the SMO^WT^-YFP or SMO^E522A^-YFP proteins (Fig. 4D lane 2 vs. 3-6, Fig. 4D’–D”, orange triangles). Whereas SMO^WT^-YFP and SMO^E522A^-YFP proteins showed gradual binding inhibition over the AA competitor dose curve, nearly all SMO^K399A^-YFP bead capture was disrupted by the lowest dose of free AA added (Fig. 4D’–D”, Supplementary Fig. 2D). These results, taken together with in silico docking predictions, support that AA can directly associate with SMO in the presence of sterol agonists and that the lysine anchor plays a crucial role in stabilizing its binding.

### Reduced AA binding attenuates ligand-activated SMO ciliary localization and signaling

**Fig. 5:**
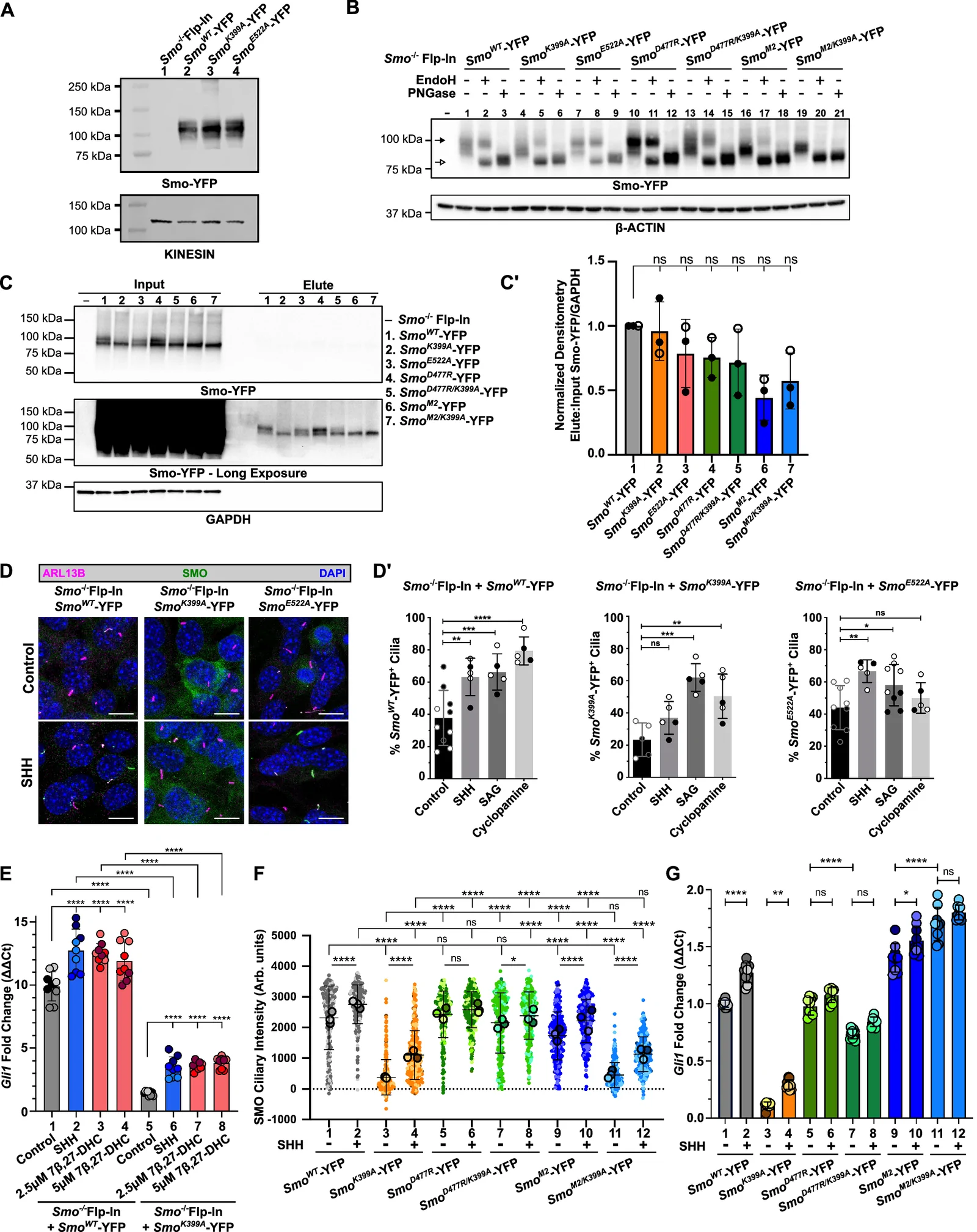
SMO^K399A^ shows reduced ligand responsiveness. **A** *Smo*^*-/-*^ Flp-In-NIH-3T3 cells were stably transfected with *Smo*^*WT*^*-YFP*, *Smo*^*K399A*^*-YFP*, or *Smo*^*E522A*^*-YFP* cDNAs. A representative immunoblot of three experiments is shown; kinesin is the loading control. **B** Cell lysates were treated with Endoglycosidase H (EndoH) or Peptide-*N*-Glycosidase F (PNGase). Open arrow indicates EndoH-sensitive ER protein species; closed arrow indicates post-ER, EndoH-resistant fractions. A representative immunoblot of three experiments is shown; β-actin is the loading control. **C**-**C’** Cell surface biotinylation was performed on the indicated cell lines. Biotinylated proteins collected on streptavidin beads were analyzed by immunoblot. GAPDH marks the cytoplasmic input. Relative cell surface SMO protein levels were quantified across three experiments by densitometry analysis. All data were plotted in (**C’**), with open circles corresponding to immunoblot in (**C**). **D** SHH-stimulated SMO ciliary translocation in the indicated cell lines. Ciliary axonemes are marked by ARL13B (magenta), SMO is green, and DAPI (blue) marks nuclei. Scale bar = 10 μm. **D’** The indicated cell lines were treated with control or SHH-N conditioned media (SHH) and vehicle, 100 nM SAG, or 10μM cyclopamine for ~18 hours, and percent SMO-positive cilia was quantified. The experiment was performed twice with 122–424 cells per condition. **E** qRT-PCR of the indicated cell lines treated with control, SHH, or oxysterols for ~2 hours. ∆∆Ct values were calculated for *Gli1* relative to *Smo*^*K399A*^-YFP control and normalized to the *Btf3* reference gene. **F** The indicated cell lines were treated with control or SHH, and SMO ciliary enrichment was scored over three experiments totaling 242-336 cells per condition. Large, open circles represent experimental averages. **G** The indicated cell lines were incubated with control or SHH for ~2 hours. ∆∆Ct values were calculated relative to control and normalized to the *Btf3* reference gene. The graph represents data from three experiments performed in triplicate. Results in all panels are plotted as mean ± S.D., with individual data point shades indicating experimental replicates. All significance was determined by one-way ANOVA: *<0.05, **<0.01, ***<0.001, ****<0.0001, and ns, *p* > 0.05. Source data are provided as a Source Data file.

Having determined that AA does not bind SMO^K399A^ as efficiently as it does the WT protein, we next sought to determine the functional consequence of reduced AA binding. To avoid ligand-independent ciliary enrichment that can result from transient high-level SMO over-expression, we knocked out endogenous *Smo* in Flp-In competent NIH-3T3 cells using CRISPR/Cas9 and then stably re-expressed WT, K399A, or E522A SMO-YFP proteins using the Flp-In system. We sorted YFP-positive cells and selected sorted cell populations stably expressing SMO^WT^-YFP, SMO^K399A^-YFP, and SMO^E522A^-YFP proteins at comparable levels (Fig. 5A). We analyzed N-linked glycosylation status and performed cell surface biotinylation experiments to test for altered trafficking or plasma membrane localization of the mutant proteins^45^. SMO^K399A^-YFP and SMO^E522A^-YFP mutant proteins harbored complex, EndoH-resistant glycans (Fig. 5B, closed arrows) and reached the cell surface similarly to SMO^WT^-YFP (Fig. 5C–C’). We then used these validated cell lines to test SMO ciliary entry and downstream signaling (Fig. 5D–E). SMO^WT-^YFP accumulated in cilia following treatment with activating ligands SHH and SAG and cyclopamine, which promotes SMO ciliary enrichment despite blocking its downstream signaling to GLI transcription factors (Fig. 5D–D’, Supplementary Fig. 3A)^46^. AA binding-deficient SMO^K399A^-YFP showed modestly reduced baseline PC enrichment and significantly attenuated PC entry in response to SHH stimulation (Fig. 5D–D’). Consistent with our previous observation that SAG does not require cPLA2α-mediated production of AA to stimulate robust SMO ciliary enrichment^18^, SMO^K399A^-YFP maintained its ability to enter cilia in response to SAG treatment. Cyclopamine-induced ciliary localization was also unaltered by the K399A mutation (Fig. 5D’, Supplementary Fig. 3A)^35^. This suggests that attenuation of SHH-stimulated SMO ciliary localization by K399A mutation is likely a specific effect of reduced AA binding, and not a result of binding pocket collapse or protein misfolding. Changes in ciliary structure were unlikely to be responsible for reduced SMO^K399A^ ciliary enrichment because PC lengths were comparable between each of the Flp-In lines (Supplementary Fig. 3A’).

To directly test whether reduced ligand-stimulated SMO^K399A^ ciliary entry resulted from disruption of AA allostery, SMO responsiveness to AA was tested in *Smo*^*WT*^ or *Smo*^*K399A*^ Flp-In cells by scoring PC enrichment following sterol stimulation in the absence or presence of AA. Likely due to higher than endogenous expression levels, sterol-stimulated SMO^WT^-YFP ciliary enrichment was higher than that observed with endogenous SMO by 2 hours post treatment (Fig. 2 compared to Supplementary Fig. 3B–B”). Nevertheless, sterol-stimulated PC enrichment of SMO^WT-^YFP trended up following AA supplementation in *Smo*^*WT*^ Flp-In cells (Supplementary Fig. 3B–B”). Conversely, SMO PC entry was modestly reduced by AA treatment in sterol-stimulated *Smo*^*K399A*^-YFP Flp-In cells (Supplementary Fig. 3C–C”), supporting that loss of the anchoring lysine reduces SMO responsiveness to AA-mediated allostery. Importantly, diminished SHH-stimulated ciliary enrichment of SMO^K399A^-YFP correlated with reduced downstream signaling. Baseline, SHH-stimulated, and 7β,27-DHC-stimulated *Gli1* expression levels were significantly reduced in *Smo*^*K399A*^ Flp-In cells compared to *Smo*^*WT*^ Flp-In cells (Fig. 5E). Altogether, these results support that direct AA-SMO binding, which is anchored by K399, enhances SMO ciliary enrichment and signaling in ligand-stimulated cells.

### Activating SMO mutations do not require AA allostery

Based on its predicted binding site at the top of the 7TM domain, we hypothesized that AA binding to SMO might induce a receptor conformation that stabilizes sterol ligand binding to the CRD or deep in the 7TM pocket. If this is the mechanism by which AA enhances sterol-mediated SMO activation, we reasoned that the functional consequence of the K399A mutation might be mitigated by introducing the D477R mutation, which is proposed to stabilize sterol binding^25,44^. The oncogenic M2 modification, which stabilizes a ligand-independent, highly active TM conformation, was also evaluated^47^. Double and single amino acid SMO-YFP mutants were stably expressed in Flp-In competent *Smo*^*-/-*^ NIH-3T3 cells as described above, and SMO N-linked glycan modifications were analyzed to confirm movement of the single and double mutant proteins out of the ER and to the plasma membrane. PNGase and EndoH treatment revealed that SMO^K399A^ and SMO^D477R^ single and double mutant variants produced EndoH-resistant fractions, consistent with normal trafficking out of the ER (Fig. 5B)^45^. Accordingly, both SMO-YFP proteins reached the plasma membrane, as indicated by cell surface biotinylation (Fig. 5C–C’). SMO^M2^ uses an unconventional secretory route that bypasses the Golgi, where complex glycans are generated^48^. Accordingly, EndoH-resistant N-linked glycosylation was not detected for either SMO^M2^ or SMO^M2/K399A^ (Fig. 5B). Reduced cell surface biotinylation of SMO^M2^ mutants compared to controls was also noted (Fig. 5C–C’).

To evaluate signaling competence of each of the single and double mutant SMO proteins, SMO-expressing cells were exposed to vehicle control, SHH conditioned media, or SAG, and effects on SMO ciliary localization and *Gli1* induction were tested (Fig. 5F, G, Supplementary Fig. 4). SMO^K399A^-YFP showed reduced baseline and SHH-stimulated ciliary localization compared to WT, D477R, or M2 SMO proteins (Fig. 5F and Supplementary Fig. 4A). Accordingly, SHH-mediated induction of *Gli1* was compromised for SMO^K399A^ relative to these three SMO-YFP proteins (Fig. 5G, lanes 3-4 vs. 1-2, 5-6, 9-10). Consistent with our previous demonstration that SAG does not require cPLA2α activity for high-level SMO activation^18^, SMO^K399A^ remained responsive to SAG, as demonstrated by GLI1 protein production (Supplementary Fig. 4B–B’). This result provides further support that the reduced SHH response of SMO^K399A^ is likely a consequence of compromised AA binding and not due to altered SMO structural integrity or collapse of the upper 7TM ligand binding pocket.

Combining the K399A mutation with the D477R sterol-stabilizing mutation^25,44^ reversed the K399A effect, allowing for stable SMO ciliary enrichment and *Gli1* induction in SMO^D477R/K399A^-YFP-expressing cells (Fig. 5F, G, green vs. orange, Supplementary Fig. 4). These results suggest that D477R-mediated stabilization of sterol ligand binding to SMO bypasses the need for AA binding to the top of the upper 7TM pocket to augment SMO ciliary enrichment or signaling.

Intriguingly, ciliary localization of SMO^M2/K399A^ phenocopied that of SMO^K399A^, showing reduced ciliary enrichment compared to WT or M2 SMO proteins (Fig. 5F, blue vs. orange, Supplementary Fig. 4A). Despite this attenuated ciliary localization, SMO^M2/K399A^ remained capable of stimulating robust ligand-independent target gene induction (Fig. 5G and Supplementary Fig. 4B–B’). These observations suggest that different modes of SMO signal control, i.e., stabilizing sterol binding (D477R) vs. inducing an activating conformation shift (M2), may have different requirements for AA-mediated augmentation of SMO ciliary enrichment. This is consistent with AA allosterically enhancing SMO activity by influencing its PC localization^18^.

### Allostery promotes in vivo SMO signaling

**Fig. 6:**
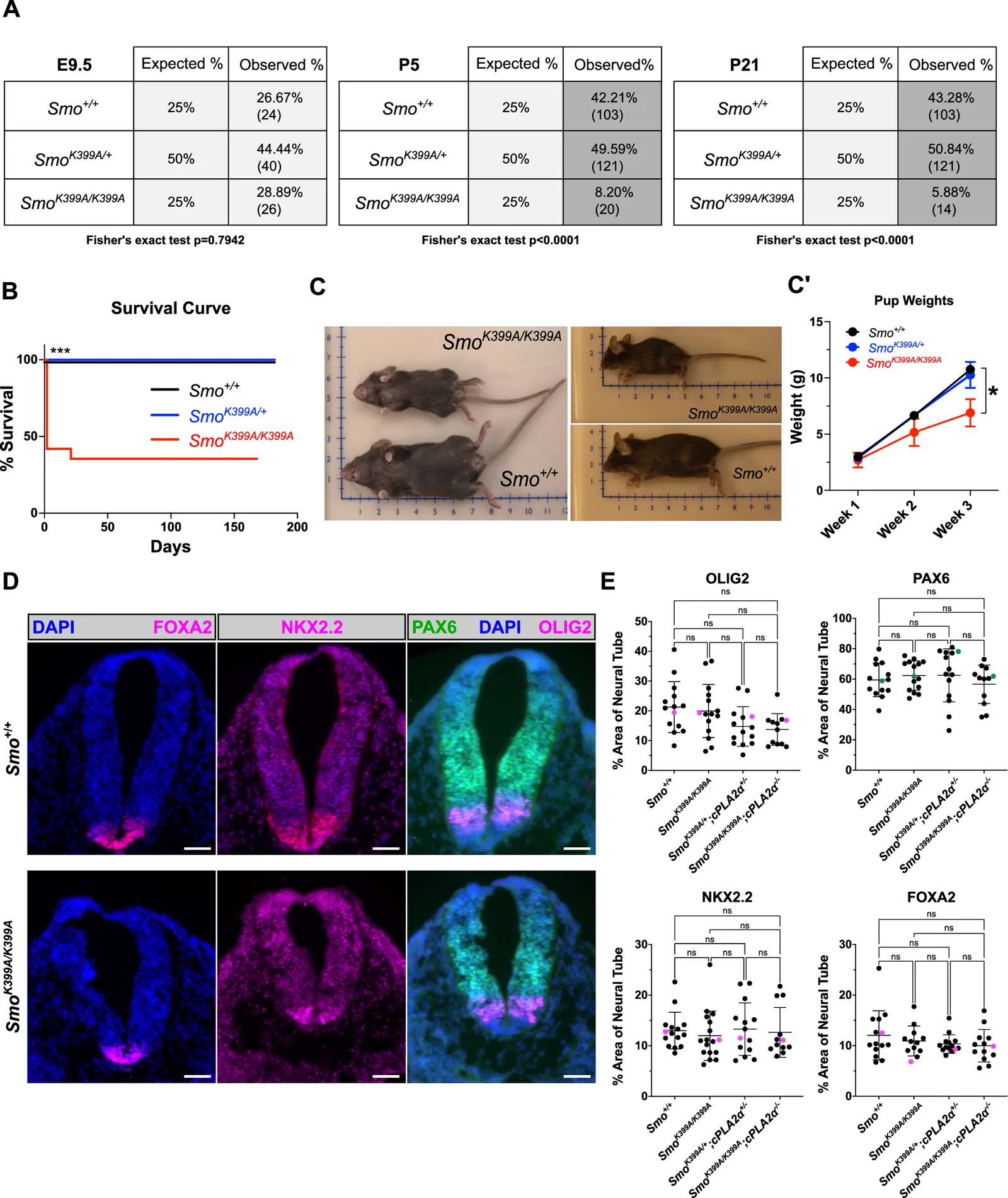
*Smo*^*K399A/K399A*^ knock-in mice fail to thrive. **A** *Smo*^*K399A/K399A*^ knock-in mice show normal Mendelian ratios at E9.5 and skewed ratios (grey) at P5 and P21. Fisher’s exact test values are shown for each time point. **B** A Kaplan-Meier survival curve shows reduced viability of *Smo*^*K399A/K399A*^ mice compared to *Smo*^*K399A/+*^ and *Smo*^*+/+*^ controls. Litters were monitored weekly from P0. *n* ≥ 200 total mice analyzed. *Smo*^*K399A/K399A*^ mice are reduced in size (**C**) and weight (**C’**) compared to controls. *n* = 2–3 mice per genotype; all mice were littermates. Mean ± S.D. is shown. **D** Cardiac level sections of developing neural tubes of E9.5/25-29 somite stage embryos were stained for the floor plate marker FOXA2 and ventral progenitor markers NKX2.2, OLIG2, and PAX6. DAPI marks nuclei. Scale bar = 50 μm. **E** Mean expression domain areas of the indicated progenitor markers were measured and normalized to overall neural tube area. A total of 3-4 embryos per genotype with 3-5 sections per embryo were analyzed. Pink/green shaded dots correspond to the representative sections shown in (**D**) and Supplementary Fig. 5E. Data are represented as mean ± S.D. Statistical significance was calculated by Log-Rank Test (**B**), two-tailed Student’s *t*-test (**C’**). and one-way ANOVA (**E**). Significance is indicated by *<0.05, ***<0.001, and ns, *p* > 0.05. Source data are provided as a Source Data file.

To interrogate whether allostery at the AA binding pocket is required for in vivo SMO signaling, we used CRISPR/Cas9 to generate a K399A knock-in mouse model and evaluated control and *Smo*^*K399A/K399A*^ mice for phenotypic indicators of altered developmental patterning. Whereas *Smo*^*K399A/K399A*^ mice were present at expected Mendelian ratios as embryos, they were underrepresented following birth (Fig. 6A, Supplementary Fig. 5A). Reduced numbers of *Smo*^*K399A/K399A*^ mice were evident by postnatal day P5 and worsened by weaning at P21 (Fig. 6A, B). Whereas no survival or overt phenotypic differences were observed between *Smo*^*+/+*^ and *Smo*^*K399A/+*^ animals, *Smo*^*K399A/K399A*^ mice that survived past birth were reduced in size and failed to thrive (Fig. 6C–C’). Accordingly, computed tomography (CT) scans revealed the skulls of *Smo*^*K399A/K399A*^ mice to be significantly smaller across several reference axes than *Smo*^*K399A/+*^ littermate controls (Supplementary Fig. 5B–B’). To evaluate protein levels in vivo, SMO was immunoprecipitated from lysates prepared from E14.5 *Smo*^*+/+*^, *Smo*^*K399A/+*^, and *Smo*^*K399A/K399A*^ embryos and analyzed by western blot using *Smo*^*-/-*^ MEFs with or without *Smo* re-expression as controls. Whereas SMO protein was undetectable in total embryo lysates, a specific band that aligned with re-expressed SMO in *Smo*^*-/-*^ MEFs was detectable in α-SMO immunoprecipitates (Supplementary Fig. 5C, lanes 1-5 vs. 6-15). Importantly, SMO protein levels in embryo immunoprecipitates were comparable between *Smo*^*+/+*^*, Smo*^*K399A/+*^, and *Smo*^*K399A/K399A*^ samples, supporting that the phenotype was likely not the result of reduced protein stability in homozygous mutant animals (Supplementary Fig. 5C, lane 7 compared to 9 and 11).

We reasoned that if decreased viability of knock-in mice resulted from attenuation of SMO signaling stemming from compromised AA allostery, combining SMO^K399A^ mutation, which binds AA poorly, with decreased SHH-stimulated AA production would likely exacerbate the observed phenotype. Thus, we obtained *cPLA2α*^*-/-*^ mice^49^ to remove activity of the AA-producing phospholipase that is activated downstream of SHH^18,21^. Due to compensation by other cytosolic PLA2 genes, *cPLA2α*^*-/-*^ mice are viable, but females exhibit reduced fertility^49^. Despite this functional compensation, removal of one or both copies of *cPla2α* in the *Smo*^*K399A/K399A*^ background significantly enhanced perinatal lethality of *Smo*^*K399A/K399A*^ mice. Only two *Smo*^*K399A/K399A*^*;cPla2α*^*-/-*^ and four *Smo*^*K399A/K399A*^*;cPla2α*^*+/-*^ mice were recovered out of 268 total animals screened (Supplementary Table 1). All surviving combination mutants were male. Curiously, we did not observe worsening of the *Smo*^*K399A/K399A*^ size reduction phenotype in the surviving *Smo*^*K399A/K399A*^*;cPla2α*^*-/-*^ mice (Supplementary Fig. 5D). We hypothesized that these surviving animals may represent phenotypic *cPla2α*^*-/-*^ escapers with compensatory phospholipase activity occurring through upregulation of other *cPla2* isoforms. This response has been documented in *cPla2α*^*-/-*^ mice that lack overt phenotypes^49^. Altogether, these results suggest that AA produced by cPLA2α in response to SHH enhances in vivo SMO signaling.

### The requirement for in vivo SMO allostery is context-dependent

Because SHH is a key regulator of multiple organs and tissues during embryonic development^50^, identifying the cause of reduced viability of *Smo*^*K399A/K399A*^ mutants necessitated empirical determination. We began by evaluating the nervous system because SHH signaling defines specific neural progenitor cell populations in the ventral neural tube^51^. We analyzed neural tubes of *Smo*^*+/+*^, *Smo*^*K399A/K399A*^, *Smo*^*K399A/+*^*;cPla2α*^*+/-*^, and *Smo*^*K399A/K399A*^*;cPla2α*^*-/-*^ E9.5/25-29 somite stage embryos for induction of the floor plate marker *FoxA2*, ventral fate markers *Nkx2.2* and *Olig2*, and intermediate fate marker *Pax6* (Fig. 6D, E, Supplementary Fig. 5E). Despite the observed viability defect, ventral progenitor domain establishment in *Smo*^*K399A/K399A*^ embryos was comparable to *Smo*^*+/+*^ controls (Fig. 6D, E). Ventral neural tube progenitor domain specification was similarly unaltered in *Smo*^*K399A/+*^*;cPla2α*^*+/-*^ and *Smo*^*K399A/K399A*^*;cPla2α*^*-/-*^ embryos (Fig. 6E, Supplementary Fig. 5E). These results suggest that the early stages of nervous system development proceed normally in mice harboring the *Smo*^*K399A*^ allele with or without combined *cPla2α* loss.

To determine whether neuronal patterning defects arose at later stages of development, we analyzed cerebella of *Smo*^*K399A/+*^ and *Smo*^*K399A/K399A*^ adult mice. No significant differences were detected in the ratios of cerebellar to total brain volumes between control and *Smo*^*K399A/K399A*^ mice (Supplementary Fig. 5F–F’). We were unable to obtain adult mice to assess cerebella of *Smo*^*K399A/K399A*^*;cPLA2α*^*-/-*^ animals due to poor viability of the compound mutants (Supplementary Table 1). Nevertheless, taken together with the results presented above, cerebellar analyses suggest that SMO allostery through the K399 anchor is not essential for neuronal cell fate specification and that reduced viability of *Smo*^*K399A/K399A*^ mice was not due to compromised neurodevelopment.

**Fig. 7:**
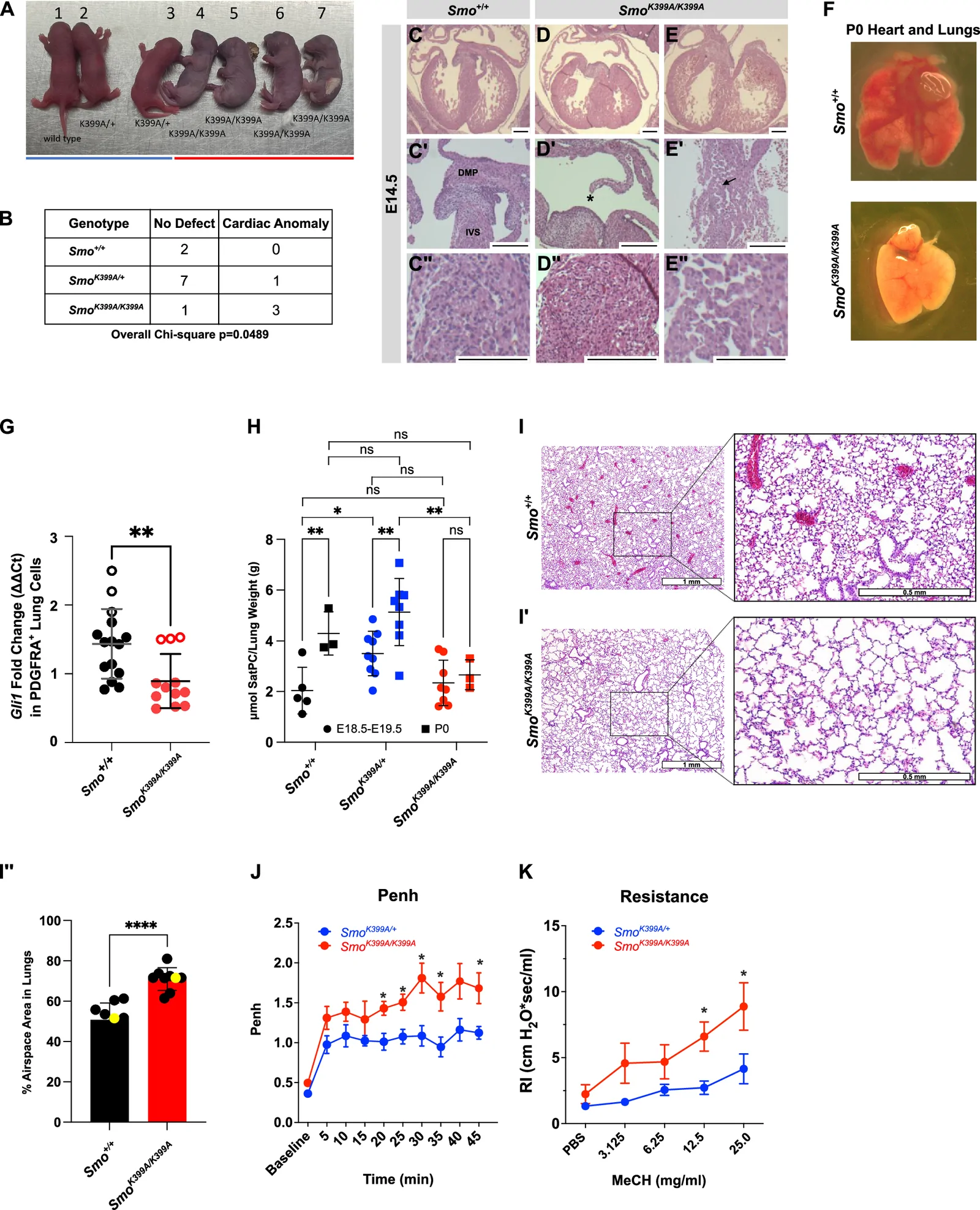
*Smo*^*K399A/K399A*^ mice exhibit cardiopulmonary defects. **A** P0 litter shows cyanotic *Smo*^*K399A/K399A*^ mice compared to *Smo*^*+/+*^ and *Smo*^*K399A/+*^ littermates. **B** Overall Chi-square analysis correlating the *Smo*^*K399A/K399A*^ mutation with cardiac mispatterning defects. **C-E”** Transverse cardiac Hematoxylin/Eosin (H&E) sections of E14.5 embryos. Whole *Smo*^*+/+*^ and *Smo*^*K399A/K399A*^ heart sections (**C**, **D**, **E**) and magnifications (**C’**-**E”**) reveal AVSD (**C’,D’**) and IVS (**C”**, **E”**) patterning defects (arrow). DMP = Dorsal Mesenchymal Protrusion, IVS = Interventricular Septum, * = Atrioventricular Septal Defect (AVSD). Scale bar = 200 µm. **F** P0 *Smo*^*+/+*^ and *Smo*^*K399A/K399A*^ hearts and lungs. **G** qRT-PCR of *Gli1* expression in PDGFRA^+^ cells isolated from *Smo*^*+/+*^ and *Smo*^*K399A/K399A*^ E18.5 (closed circles) and p0 (open circles) lungs. Fold-change was determined using the ∆∆Ct method based on *Btf3* or *Ppia* reference genes. Three technical replicates were performed per individual (*n* = 5 *Smo*^*+/+*^ and 4 *Smo*^*K399A/K399A*^). Mean ± S.D. is plotted. **H** Saturated phosphatidylcholine (SatPC) was measured in E18.5-E19.5 vs. P0 lungs. Total SatPC was normalized to lung weight. Three to nine mice per genotype per time point were analyzed, and mean ± S.D. is plotted. H&E stains of adult (P27) lung sections from *Smo*^*+/+*^ (**I**) and *Smo*^*K399A*/K399A^ (**I’**) mice. Scale bar = 1 mm (left) or 0.5 mm (insets, right). **I”** Quantification of airspace area in lung sections from *Smo*^*+/+*^ and *Smo*^*K399A*/K399A^ animals. One to two randomly chosen 8x zoom regions from 5 mice were evaluated per genotype. Results are plotted as mean ± S.D., and the yellow dots indicate the sections shown in (**I**-**I’**). **J** *Smo*^*K399A/+*^ and *Smo*^*K399A/K399A*^ mice were subjected to whole-body plethysmography. Enhanced pause (Penh) values were recorded over 45 minutes at 5-minute intervals. Results are plotted as maximal fold increase in Penh relative to baseline and expressed as mean ± S.E., where *n* = 4 mice per group. **K** Airway resistance was evaluated before and after exposure to the indicated concentrations of aerosolized methacholine (MeCH) in *n* = 6 *Smo*^*K399A/+*^ and *n* = 7 *Smo*^*K399A/K399A*^. Results are plotted as mean ± S.E. Significance was calculated using a two-tailed Student’s *t*-test (**G**, **I”**, **J**, **K**) and two-way ANOVA (**H**), and is denoted as *<0.05, **<0.01, ****<0.0001, and ns, *p* > 0.05. Source data are provided as a Source Data file.

In addition to its contributions to nervous system development, SHH also plays important roles during patterning of the heart and lungs^5,52–54^. During heart development, SHH drives heterochronic gene expression to ensure proper timing of second heart field (SHF) progenitor differentiation and morphogenesis^6^. In the lung, SHH signaling is active during three key stages of lung morphogenesis, including initial budding from the foregut around E9.5, branching morphogenesis beginning around E11.5, and alveolar remodeling at birth^5,7,55^. We reasoned that the high perinatal lethality associated with SMO^K399A^ mutation could result from compromised cardiopulmonary development, as suggested by observed “gasping” and a cyanotic appearance of P0 *Smo*^*K399A/K399A*^ mice (Fig. 7A). To probe for indicators of altered heart or lung development in *Smo*^*K399A/K399A*^ embryos, we performed bulk RNAseq analysis on E9.5 *Smo*^*K399A/K399A*^ and *Smo*^*+/+*^ littermate control embryos (Supplementary Fig. 6A–F’). Among the top pathways upregulated in *Smo*^*K399A/K399A*^ embryos were heart development, TGFβ signaling, and prostaglandin signaling, which is driven by AA metabolism. Top downregulated pathways included multiple genes related to PC function and ciliopathies (Supplementary Fig. 6A–F’), which underscores the essential relationship between the SHH pathway and primary cilium fitness^8,9,21^.

Given the established link between SHH signaling, ciliopathies, and CHD^56–58^, we hypothesized that disruption of ciliary SMO signaling in *Smo*^*K399A/K399A*^ embryos might lead to compromised heart development. To interrogate this, hearts of E14.5 *Smo*^*+/+*^ and *Smo*^*K399A/K399A*^ embryos were subjected to histological examination. Atrioventricular septum defects (AVSD) were observed in 3 of 4 mutant embryos analyzed (Fig. 7B, C-C" vs. D-D”, asterisk). We also observed mispatterning of the interventricular septum (IVS) in *Smo*^*K399A/K399A*^ embryos (Fig. 7C’-C” vs. E’-E”, arrow). These defects are consistent with the known manifestations of reduced SHH signaling during cardiac progenitor field establishment^6^. Moreover, the upregulation of heart development and differentiation drivers including *Tbx5, Myocd*, and *Cyp26c1* observed in the RNAseq data sets from E9.5 *Smo*^*K399A/K399A*^ embryos is consistent with failed heterochronic differentiation control (Supplementary Fig. 6A, B, D, E)^6,59^.

Significant signal crosstalk occurs between the developing heart and lungs, suggesting that lung development might also be compromised in *Smo*^*K399A/K399A*^ mice^60^. Consistent with this hypothesis, lungs of P0 *Smo*^*K399A/K399A*^ mice showed altered morphology compared to controls (Fig. 7F). To evaluate SHH signaling activity in developing lungs, we purified SHH-responsive PDGFRA^+^ fibroblasts from lungs of E18.5-P0 *Smo*^*+/+*^ and *Smo*^*K399A/K399A*^ embryos and then quantified *Gli1* expression in the PDGFRA^+^ population by qPCR analysis (Fig. 7G and Supplementary Fig. 6G)^61^. *Gli1* expression was significantly reduced in *Smo*^*K399A/K399A*^ mutants compared to control, indicating compromised SHH activity in prenatal lungs (Fig. 7G)^62^. To determine whether reduced SHH signaling activity in PDGFRA^+^ fibroblasts led to alveolar maturation defects in *Smo*^*K399A/K399A*^ neonates, saturated phosphatidylcholine (SatPC) was measured as a primary index of lung surfactant production in embryonic and P0 mice. Surfactant secretion from alveolar type II (AT2) cells at birth facilitates the transition to air breathing by lowering air-liquid surface tension and preventing alveolar collapse^63^. Failure of AT2 cells to produce or secrete surfactant compromises this essential transition, which can result in gasping or inability to breathe at birth^64^. Although the precise signals that stimulate surfactant secretion in vivo are incompletely defined, in vitro studies suggest that cPLA2α and AA signaling may be involved^65,66^. Accordingly, we found that while *Smo*^*+/+*^ and *Smo*^*K399A/+*^ mice increased surfactant SatPC levels at birth, *Smo*^*K399A/K399A*^ mice, which we hypothesize have reduced SMO responsiveness to AA, did not show a SatPC shift (Fig. 7H). Although *Smo*^*K399A/+*^ animals showed elevated SatPC levels compared to *Smo*^*+/+*^ control, SatPC showed a comparable fold increase between the E18.5-19.5 baseline and at birth, suggesting that control of surfactant production and release is intact in *Smo*^*K399A/+*^ mice. We do not know the reason for the increased baseline SatPC levels in heterozygous animals but speculate that altered AA metabolism and/or compensatory AA signaling in *Smo*^*K399A/+*^ animals may contribute.

Although most *Smo*^*K399A/K399A*^ mice died during the perinatal period, those that survived to P21 typically reached adulthood (Fig. 6B). To determine whether the surviving *Smo*^*K399A/K399A*^ mice showed evidence of pulmonary mispatterning, we evaluated alveolar morphology in lung sections from control and surviving *Smo*^*K399A/K399A*^ mice. Hematoxylin and Eosin (H&E) staining of adult lungs revealed alveolar simplification in *Smo*^*K399A/K399A*^ lung sections, suggestive of reduced efficiency of gas exchange (Fig. 7I–I”)^7^. To evaluate the consequence of altered alveolar patterning in mutant mice, we performed unrestrained whole-body plethysmography (WBP) on control and surviving *Smo*^*K399A/K399A*^ adults^67^. WBP revealed that *Smo*^*K399A/K399A*^ mice had significantly higher Penh scores compared to *Smo*^*K399A/+*^ control animals, indicating increased airway hyperresponsiveness and altered lung function (Fig. 7J). To test whether this physiological abnormality correlated with altered lung mechanics, *Smo*^*K399A/K399A*^ and control animals were intubated and exposed to methacholine (MeCH), and airway resistance was measured^68^. Airway resistance was significantly increased in *Smo*^*K399A/K399A*^ mice compared to *Smo*^*K399A/+*^ control animals, indicating higher susceptibility to bronchoconstriction (Fig. 7K). Due to high embryonic lethality, we were unable to obtain sufficient numbers of adult *Smo*^*K399A/K399A*^*;cPla2α*^*-/-*^ animals for lung functional analyses. Nevertheless, the observed cardiac phenotypes combined with neonatal and adult lung analyses of *Smo*^*K399A/K399A*^ mice suggest that in vivo SMO allostery contributes to optimal SHH signaling during cardiopulmonary development.

## Discussion

Allosteric regulation of receptor and/or downstream enzymatic effector activity provides cells with the ability to tune the amplitude of a ligand-induced response to meet context-specific signaling requirements. This may be one mechanism by which morphogens, which signal in time- and concentration-dependent manners, pattern tissues with temporal and spatial precision. Consistent with this hypothesis, the signal transducer activated downstream of the SHH morphogen, the GPCR SMO, can be controlled through distinct orthosteric and allosteric ligand binding pockets^10^. The results presented here provide support for in vivo SMO allostery during cardiopulmonary development. We identified a conserved lysine residue in EC2 as an anchoring residue that stabilizes AA binding to a pocket at the top of the 7TM domain. We demonstrate that compromising AA allostery through mutation of this residue attenuates SHH-stimulated SMO ciliary enrichment, which blunts downstream signaling and triggers cardiopulmonary phenotypes in knock-in mice harboring a lysine-to-alanine anchor mutation.

Although SHH signaling is a known contributor to heart and lung development, its dysfunction is most linked to compromised neurodevelopment^3,69^. As such, it was unanticipated that neural tube patterning and cerebellar development would be unaltered in *Smo*^*K399A/K399A*^ mice and that these animals would instead present with cardiopulmonary mispatterning. One interpretation of this result is that the need for SMO allostery is context-specific in vivo. Cell fate determination during ventral neural tube patterning occurs in response to SHH-mediated activation of highly robust gene regulatory networks that reinforce neuronal fate choices over time^70^. Thus, the neural tube may have sufficient temporal and spatial feedback mechanisms in place to compensate for hypomorphic activity of the SMO^K399A^ mutant protein. By comparison, heart and lung development depends on strong bursts of SHH signaling at distinct developmental time points when crucial patterning decisions are made. In the heart, first and second heart fields are directed to cardiomyocyte fates as early as E7.25, but SHF commitment must be delayed until ~E10.0 so the structures that are necessary for pulmonary circulation specify at the correct time and place^71,72^. SHH instructs this precise timing such that delay or reduction of signal activation results in precocious SHF differentiation, which leads to cardiac mispatterning and AVSD like that observed in *Smo*^*K399A/K399A*^ embryos^6,59^.

Lung morphogenesis is tightly linked to cardiac development and also requires bursts of SHH signaling activity at distinct developmental stages. SHH stimulates initial lung budding from the foregut around E9.5 and contributes to branching morphogenesis as prenatal lung patterning proceeds^4,5^. Strong and rapid induction of SHH pathway activity is again necessary during the neonatal period when surfactant secretion occurs, and the alveoli begin to mature^73^. Failure to induce SHH signaling at this stage leads to alveolar simplification akin to what is observed in *Smo*^*K399A/K399A*^ mice.

Intriguingly, lungs of *Smo*^*K399A/K399A*^ neonates had reduced SatPC levels at birth compared to controls. Although SHH has not been previously linked to regulation of lung surfactant, AA is reported to stimulate surfactant release from AT2 cells^65,66^. This suggests that SHH may directly contribute to surfactant secretion through its ability to stimulate cPLA2α to produce AA. Alternatively, robust SMO signaling downstream of SHH may be necessary to facilitate alveolar epithelial cell-to-fibroblast communication circuits that are required for specification of lipofibroblasts that contribute to surfactant production^7^. Future studies will be required to test these hypotheses and delineate the signal synergy occurring between SHH and other AA-impacted pathways during this crucial stage of alveolar morphogenesis.

Computer docking places AA in a binding cavity at the top of the 7TM bundle and suggests that AA, cyclopamine, and the small molecule SMO agonist SAG likely share this versatile binding pocket. Consistent with this prediction, we previously demonstrated that AA competes with cyclopamine for SMO binding and found that SAG-mediated signaling is less sensitive to cPLA2α inhibition compared to oxysterol- or SHH-induced pathway activity^18^. The current study suggests that AA may serve as a broadly employed allosteric enhancer of sterol/oxysterol-induced SMO activity because 7β,27-DHC, CHO:MβCD, and 24(S),25-EpCHO SMO responses are all blunted by cPLA2α inhibition. The predicted AA pocket is spatially distinct from where 7β,27-DHC, 24(S),25-EpCHO, and CHO bind the CRD and is offset from the 24(S),25-EpCHO and CHO binding cavities in the 7TM core^26–28^. These results informed the hypothesis that AA and each sterol can occupy their respective binding pockets simultaneously for enhanced SMO ciliary retention. Accordingly, AA supplementation can augment 7β,27-DHC, CHO:MβCD, or 24(S),25-EpCHO-stimulated SMO ciliary accumulation within ~2 hours of sterol treatment. Intriguingly, augmentation of SMO ciliary enrichment by 24(S),25-EpCHO, which is predicted to bind to pockets in the CRD, 7TM domain, and IC loops, was less pronounced than what occurred for the other sterol agonists. This reduced allosteric effect may stem from the close proximity of AA and 24(S),25-EpCHO binding pockets in the 7TM domain^26^. We speculate that the active SMO conformation conferred through ligand occupancy near the top of the 7TM bundle may be satisfied by 24(S),25-EpCHO binding, thereby lessening the effect of AA allostery. Nevertheless, our data support that AA can enhance 24(S),25-EpCHO-activated SMO signaling because cPLA2α inhibition significantly blunts SMO PC accumulation and *Gli1* induction in 24(S),25-EpCHO-treated cells. Moreover, AA bead capture assays demonstrate that SMO remains associated with AA even in the presence of high concentrations of 24(S),25-EpCHO, indicating that the two lipids can likely bind simultaneously. Whether AA binds cooperatively with 24(S),25-EpCHO in the 7TM or restricts 24(S),25-EpCHO occupancy to the CRD and/or IC loop pockets is not known. However, redirection of the oxysterol to the CRD or IC loops could provide an explanation for reduced allosteric impact of AA on 24(S),25-EpCHO-stimulated SMO because AA binding would simply switch one upper 7TM binding lipid for another on the activated receptor.

Notably, the murine K399 AA anchoring residue corresponds to K395 of human SMO, which is suggested to function as a coordinating residue for “super stabilizer” ligands that place TM helix 4 in a position that facilitates essential communication between the CRD and EC loops^24,74^. Thus, we propose that AA allostery through the upper 7TM binding pocket likely stabilizes the active SMO conformation that favors the CRD to EC4 communication “bridge”. That AA likely functions to promote active conformations induced by CRD and deep 7TM binding sterol agonists is suggested by the ability of activating mutations in the 7TM domain, D477R and M2, to restore signaling by SMO^K399A^. Intriguingly, whereas the sterol “lock” D477R mutation restored SMO^K399A^ PC entry to normal levels, the M2 mutation, which induces constitutive ligand-independent SMO signaling through an activating 7TM conformation shift, did not. Nevertheless, SMO^M2/K399A^ induced significant ligand-independent *Gli1* activation. This could suggest rapid recycling of constitutively active SMO^M2/K399A^ into and out of the PC or, alternatively, indicate a strict requirement for 7TM lipid ligand occupancy for SMO PC retention.

The insensitivity of neural tube patterning to SMO^K399A^ mutation suggests that AA allostery is dispensable in contexts where the SHH response is reinforced by continuous ligand exposure. This is likely the case in the developing neural tube where SHH activates sophisticated transcriptional regulatory networks designed to buffer perturbations across the signaling gradient^51,70^. Thus, we speculate that allosteric enhancement of SMO signaling activity is more important in developmental contexts where rapid bursts of elevated SHH pathway activity are required, like the developing heart and lungs^4–6,52,55^. A need for optimal control of SMO signaling output in the heart and lungs is further supported by knockout mouse models of other SMO signal “calibrators”, MEGF8 and MGRN1^75^. These proteins interact to form a ubiquitin regulatory complex that directs SMO degradation. Loss of complex activity allows for ligand-independent SMO ciliary localization that heightens SMO responsiveness to SHH, resulting in gain-of-function phenotypes in the developing heart and lungs. Like what is observed for *Smo*^*K399A/K399A*^ animals, neural tube patterning is unaffected by either MEGF8 or MGRN1 loss^75,76^. As such, we propose that cardiopulmonary development is highly sensitive to SMO signal perturbation and suggest that receptor allostery is one context-specific mechanism by which SHH ensures that optimal signaling thresholds occur during developmental tissue patterning.

## Methods

### Ethics Statement

Data and materials generated from animals were obtained in accordance with the IACUC-approved St. Jude Children’s Research Hospital protocol 608-100616-10/19. All animal husbandry and procedures were performed in accordance with protocols approved by St. Jude Children’s Research Hospital (SJCRH).

### Cell Culture

Unless otherwise indicated, all cell lines were grown in Dulbecco’s Modified Eagle Medium (DMEM) supplemented with 10% bovine calf serum (BCS), 2 mM *L*-glutamine, 1 mM sodium pyruvate, 1X non-essential amino acids, and 1% penicillin-streptomycin solution (Thermo Fisher Scientific). Cells were routinely passaged using a 0.25% Trypsin/EDTA solution and maintained in a humidified incubator at 37 °C with 5% CO_2_. All cell lines were regularly tested for mycoplasma contamination using MycoAlert (Lonza). To promote ciliogenesis, cell lines were serum-starved for 2 hours in serum-free media (DMEM supplemented with 2 mM *L*-glutamine, 1 mM sodium pyruvate, 1X non-essential amino acids, and 1% penicillin-streptomycin solution). Media was replaced with low serum (0.5% BCS) media, and cells were treated for the indicated times following serum starvation.

### Cloning

Murine *Smo* was amplified from pCS2-*Smo-YFP* and inserted in frame to generate the pEF5/FRT-*Smo-YFP* backbone using the pEF5/FRT/V5 Directional TOPO™ Cloning Kit (Thermo Fisher Scientific). Site-directed mutagenesis with the QuikChange XL kit (Agilent Technologies) was used to introduce K399A and E522A substitutions into the constructs. D477R, D477R + K399A, M2, and M2 + K399A substitutions were created in the pEF5/FRT-*Smo-YFP* backbone through the custom cloning services of GenScript Biotech.

### Cell line generation

NIH-3T3 *Smo*^*-/-*^ Flp-In cells have been previously described^77^. Clonal cell lines stably expressing the proteins of interest were developed using the Flp-In system (Thermo Fisher Scientific). pEF5 FRT *Smo*^WT^*-*YFP, *Smo*^*K399A*^*-*YFP, *Smo*^*E522A*^*-*YFP, *Smo*^*D477R*^*-*YFP*, Smo*^*M2*^*-*YFP*, Smo*^*D477R+K399A*^*-*YFP, *or Smo* ^*M2+K399A*^*-*YFP and pOG44 Flp-recombinase vector were transfected using Lipofectamine 3000 (Thermo Fisher Scientific) and selected using Hygromycin B. After chemical selection, cell lines were sorted on the BD FACSDiscover™ S8 for mid to low YFP expression. All cell lines were routinely validated using functional assays and western blotting as appropriate.

### Chemical preparation and treatment

Oxysterols (Avanti Polar Lipids) and *N-*stearoyldopamine (Cayman Chemical) were resuspended in DMSO. Cyclopamine, SAG, and arachidonic acid (Cayman Chemical) were dissolved in ethanol. Giripladib (GIRI) was synthesized as described^21^. Resuspended chemicals were stored at −20 °C.

To prepare water-soluble CHO: MβCD complexes, 40 mM CHO and MβCD stock solutions were made. CHO was dissolved in ethanol, and MβCD was dissolved in low serum media. A glass vial was prepared with 2.5 mM of CHO from the organic stock solution. Nitrogen gas was streamed over the CHO solution until the organic solvent was evaporated completely, generating a thin film in the vial. One mL of 25 mM MβCD was added to the dried CHO film in the glass vial. The solution was vortexed to dissolve the mixture until it became clear. Solutions were passed through a 0.2 μm filter and stored in glass vials at 4 °C.

SHH-N-conditioned media were collected from HEK293T cells stably expressing the amino-terminal signaling domain of SHH^78^.

For ciliary localization and qPCR experiments, cells were pretreated with either vehicle, GIRI, or cyclopamine in serum-free media for 2 hours. Media was replaced with low serum media containing vehicle, GIRI, SHH-N conditioned media (100 µL/mL), SAG (100 nM), cyclopamine (10 µM), oxysterols, or CHO:MβCD, and incubated for the indicated time points prior to analysis. For short-term incubations, cells were typically pretreated for 30 mins with AA or sterols.

### Expression vectors and transient transfection

SSTR3-GFP (Addgene #49098)^79^ and ARL13B-GFP (Addgene #40879)^80^ expression constructs were transfected into NIH-3T3 cells 24 hours prior to serum starvation and drug treatments. Transfection of plasmid DNA for transient protein expression was performed using Lipofectamine 3000 (Thermo Fisher Scientific) per the manufacturer’s instructions.

### Immunofluorescence

Cells were cultured on coverslips (Corning) for 18 hours prior to any chemical treatments and were washed with PBS prior to fixation in 4% paraformaldehyde for 12 min. Cells were then washed three times with PBS for 5 min each and incubated in blocking buffer (PBS with 0.1% Triton X-100 and 5% goat or donkey serum) for 60 min at room temperature.

Primary antibody incubations were carried out overnight at 4 °C using the following antibodies and dilutions: anti-SMO (Santa Cruz, 1:500), anti-acetylated-α tubulin (Cell Signaling, 1:1000), anti-IFT88 (Proteintech, 1:500), anti-p-cPLA2 (Cell Signaling, 1:1000), anti-gamma tubulin (Abcam, 1:1000), and/or anti-ARL13B (BiCell Scientific, 1:1000) in wash buffer (PBS with 0.1% Tween-20 + 5% goat or donkey serum). Secondary antibody incubations were performed with AlexaFluor 488, 555, and 647 (1:1000) conjugated secondary antibodies (Thermo Fisher Scientific) and DAPI for 60 min at room temperature. Following secondary antibody incubation, coverslips were washed with wash buffer three times for 5 min each and mounted using ProLong Glass Antifade Mountant (Thermo Fisher Scientific). Microscopy images were captured using a TCS SP8 STED 3X confocal microscope (Leica) or a Nikon A1R confocal microscope. For all immunofluorescence experiments, ≥50 cells were examined over at least two experiments, with representative images shown.

### Quantification of ciliary length and fluorescent intensity

For ciliary length quantification, individual ARL13B or ac.-αTubulin signals were traced using the FIJI/ImageJ line selection tool and lengths were recorded. For ciliary SMO/SSTR3/IFT88/ARL13B signal intensity determination, each cilium surface area was traced from the base to the tip using the spline profile function of LASX or FIJI/ImageJ software. The average fluorescence measurement adjacent to the cilium was subtracted from ciliary fluorescence to correct for background. The intensity values along the cilium were exported and analyzed via GraphPad Prism version 10-11.0.2 (GraphPad Software, La Jolla, CA, USA).

### Ligand docking

The published ligand-bound SMO structures were obtained from the Protein Data Bank entries 5L7D (CHO-bound), 6D32 (cyclopamine-bound), and 6O3C (structure of active Smoothened bound to SAG21k, CHO, and NbSmo8), and subsequently loaded into Maestro (Schrödinger Release 2019-3). To prepare the protein for docking, the protein preparation wizard was used to assign bond orders, add hydrogens, create disulfide bonds, and fill in missing side chains and loops. Default parameters were used for optimization of hydrogen bond assignment (sampling of water orientations and use of pH 7.0). Waters beyond 5 Å of het groups or with fewer than three hydrogen bonds to non-waters were removed. Restrained energy minimization was then applied using the OPLS3e forcefield^81^. Prepared protein systems were further checked with Ramachandran plots to ensure there were no steric clashes. To generate receptor grids for docking, the ligand respective to each structure was first selected as the grid-defining ligand (cholesterol or cyclopamine). Default Van der Waals radius scaling parameters were used (scaling factor of 1, partial charge cutoff of 0.25). Chemical structures of CHO, AA, and cyclopamine were obtained from the PubChem database (https://pubchem.ncbi.nlm.nih.gov/) as SMILES identifiers, and subsequently loaded into the LigPrep panel of Maestro (to convert to a 3D structure at target pH of 7.0 ± 2.0 and retaining specified stereochemical properties). Ligands were then docked using the most stringent docking mode (extra precision, “XP”) of Glide^82^, with the following parameters: dock flexibly, perform post-docking minimization, and keep 100% of scoring compounds.

### Molecular dynamics simulations

The arachidonic acid-bound SMO from *Xenopus* (PDB: 6D32) was simulated for 20 nanoseconds using Desmond (Schrödinger Release 2019-3). All parameters, including solvent model, force field, trajectory recording intervals, system energy, ensemble class, temperature, and pressure, were set according to Wright et al., 2020^42^.

### Sequence and structure alignments

The Clustal W2 program was used to align *Xenopus* (UniProt A0A974HT42), human (UniProt Q99835), mouse (UniProt P56726), rat (UniProt P97698), chicken (UniProt O42224), zebrafish (UniProt Q5RH73), and fruit fly (UniProt P91682) SMO sequences. The structural overlay was generated using PyMOL^©^ Molecular Graphics System to align SMO PDB structures 5L7D (human), 6D32 (*Xenopus*), and 6O3C (mouse). Arachidonic Acid PubChem CID: 444899; 7β,27-DHC PubChem CID: 52931518; 24(S),25-EpCHO PubChem CID: 3247059; Cholesterol PubChem CID: 5997

### Immunoblotting

Cells were washed once with cold PBS and lysed in RIPA buffer (150 mM sodium chloride, 1.0% NP-40, 0.5% sodium deoxycholate, 0.1% sodium dodecyl sulfate, 50 mM Tris, pH 8.0) supplemented with EDTA-free complete protease inhibitor cocktail tablets (PIC, Roche). Lysates were sheared 10 times with a 23 G needle and kept on ice for 30 min at 4 °C. Centrifugation at 14,000 x *g* at 4 °C for 20 min was performed post-incubation, and the supernatant was collected and quantified for protein concentration using the Pierce BCA Protein Assay Kit (Thermo Fisher Scientific).

Samples were diluted in 5X SDS sample buffer, run on 4–15% Tris-Glycine SDS-PAGE gels (Bio-Rad), and transferred to Immobilon-P PVDF membranes (Millipore) using Tris/Glycine buffer (Bio-Rad) at 100 V for 30 min. Membranes were blocked with 5% nonfat dry milk in Tris-buffered saline with 0.1% Tween-20 (TBST) for 1 hour at room temperature. Primary antibody incubations were performed at 4 °C overnight with the following dilutions: mouse anti-SMO (Santa Cruz, 1:500), rabbit anti-Kinesin (KIF5B, Abcam, 1:5000), mouse anti-GLI1 (Cell Signaling, 1:1000), mouse anti-β-actin (Santa Cruz, 1:500), rabbit anti-GAPDH (Cell Signaling Technology, 1:2000), or rabbit anti-GFP (Rockland, 1:8000). Blots were washed three times in TBST and incubated for 1 h with corresponding HRP-conjugated secondary antibodies (Jackson ImmunoResearch Labs, 1:5000). Blots were developed using the Odyssey Fc imaging system (Li-COR) or Chemi-Doc imaging system (Bio-Rad) with the SuperSignal^TM^ West Pico PLUS Chemiluminescent Substrate (Thermo Fisher Scientific). GLI and SMO intensities were analyzed by densitometry using FIJI/ImageJ and calculated as GLI1:SMO divided by densitometry of GAPDH. All experiments were conducted a minimum of three times. Normalized data were pooled and plotted using GraphPad Prism v10-11.0.2.

### Arachidonic acid bead capture assay

AA beads were freshly prepared by click chemistry using the Click-iT cell reaction buffer kit (Invitrogen). Azide-coated agarose beads (Click Chemistry Tools) were washed three times in H_2_O and then resuspended in Click-iT reaction mix containing AA alkyne (Cayman Chemical) according to manufacturer’s protocols. Reactions were incubated at RT for 1 hour with inversion, and then beads were washed once in washing/binding buffer (10 mM Tris-HCl pH 7.4, 150 mM NaCl) with 0.25% NP-40 and twice in washing/binding buffer without NP-40. The beads were resuspended in washing/binding buffer and inverted at 4°C during lysate preparation.

Membrane fractions were prepared as described previously^18^. HEK293T cells were seeded at a density of 2 ×10^6^ cells/10 cm dish in DMEM + 10% FBS. One microgram of pEF5 FRT *Smo*^*WT*^-YFP, pEF5 FRT *Smo*^*K399A*^-YFP, pEF5 FRT *Smo*^*E522A*^-YFP, or pEF5B FRT AP DEST *Sstr3*-EGFP was transfected into HEK293T cells using FuGene HD (Promega) according to the manufacturer’s instructions. Twenty-four hours later, media was changed to DMEM + 0.5% FBS for 24 hours. Cells were harvested and swelled in 1 mL hypotonic SEAT buffer (250 mM sucrose, 1 mM EDTA, 10 mM acetic acid, 10 mM triethanolamine pH 7.4, and SigmaFast EDTA-free PIC) for 30 min on ice. Cells were lysed with 40 strokes using a tight Dounce pestle twice (80 total per sample) and transferred to a microfuge tube. Lysates were needled 10 times with a 26 G blunt needle and then centrifuged 900 x *g* for 5 min at 4 °C. Membranes were isolated by ultracentrifugation (95,000 x *g*, 37 min at 4 °C) and solubilized in n-dodecyl-β-D-maltopyranoside (DDM) buffer (50 mM Tris pH 7.4, 150 mM NaCl, 10% glycerol, 0.5% DDM, and SigmaFast EDTA-free PIC) for 30 min on ice with inversion. To further solubilize membranes, the pellets were resuspended with 10 passes through a 26 G blunt needle. Membranes were centrifuged at 14,000 x *g* for 15 min at 4 °C to remove insoluble protein. Supernatant (DDM-membrane prep) was then transferred to a fresh microfuge tube. Protein concentration was determined by BCA assay; an input sample was set aside, and 1 μg protein was used per reaction in the pull-down assay. The supernatant was precleared as a pool with 50 μL of a 50% unclicked agarose bead slurry, pre-washed in H_2_O, and resuspended in DDM buffer for 30 minutes at 4 °C with inversion.

After preclearing, the supernatant was aliquoted to individual 1.5 mL tubes for each reaction. Samples were preincubated with the indicated compounds or ethanol vehicle for 15 minutes on ice. Then, 50 μL of a 50% bead slurry was added to each reaction and rotated for 1.5 hours at 4 °C. The beads were washed two times at 1000 x *g* for 2 min at 4 °C with 0.5X DDM/0.5X Binding Wash Buffer (50 mM Tris pH 7.4, 150 mM NaCl, 5% glycerol, 0.25% DDM, and SigmaFast EDTA-free PIC) and one time with 0.5X DDM/0.5X Binding Wash Buffer + 0.125% NP-40. Bound proteins were eluted in SDS-PAGE sample buffer with 50 mM DTT at RT with inversion for 10 min. The beads were pelleted at 1000 x *g* for 2 min at 4 °C, and the eluate was transferred to fresh microfuge tubes for analysis via SDS-PAGE. Samples were run on 4-15% TGX Criterion gels (Bio-Rad) and transferred onto methanol-activated Immobilon-P PVDF membranes (Millipore) using Tris/Glycine Buffer containing 20% Methanol at 100 V for 45 min.

For densitometry analysis, western blot intensities were obtained by quantifying the chemiluminescence count per square millimeter (I = counts per mm^2^) using Photoshop (Adobe) and normalized to the intensity of protein bound to beads in the absence of chemical treatment.

### Cell surface biotinylation

*Smo*^*-/-*^ cells stably expressing various *Smo* constructs were seeded in 2 10-cm dishes per cell line and allowed to grow overnight. Cells were washed twice with cold 1x PBS pH 7.4 and incubated for 30 min rocking at room temperature in 3 mL of 1x PBS containing 0.8 mg/ml EZ-Link Sulfo-NHS-SS-Biotin (Thermo Fisher Scientific). Biotinylation was quenched by washing cells twice with cold 1x PBS containing 100 mM glycine. Cells were lysed in RIPA buffer and protein concentrations were determined using the BCA assay. Lysates were incubated with 40 μL of Streptavidin agarose beads (Pierce) for 1 hour at 4 °C. Beads were washed three times with RIPA buffer and bead-purified proteins were extracted in 2X sample buffer containing 2 mM free biotin. Immunoblot SMO intensities were analyzed by densitometry using Fiji/ImageJ and calculated as elution SMO:input SMO divided by densitometry of GAPDH. All experiments were conducted three times. Normalized data were pooled and plotted using GraphPad Prism v10-11.0.2.

### Glycosidase treatments

RIPA lysates from *Smo*^*-/-*^ Flp-In cells were incubated with 1000 U of Peptide-*N*-Glycosidase F (PNGase F) or 1000 U of Endoglycosidase H (EndoH) for 2 hours at room temperature before being subjected to SDS-PAGE and western blot analysis. All enzymes were sourced from New England Biolabs.

### Mouse models

The animals in this study were housed in micro-isolation cages in barrier conditions. The facility has an isolated ventilation system and maintains a 12 hour light/12 hour dark schedule via an automated light system. All animals were fed standard chow and provided water ad libitum. Temperature and humidity are controlled via in-room thermostats with continuous monitoring by an electronic digital thermometer/humidistat. Alarms notify if fluctuations occur outside of the defined range, set in accordance with National Research Council guidelines (NRC 1996), and technicians record temperature and humidity daily.

*cPLA2α*^*-/-*^ mice have been previously described^49^.

### Smo^K399A^ mouse generation

The *Smo*^*K399A*^ mouse model was created using CRISPR-Cas9 technology and direct zygote injection at the SJCRH Genetically Engineered Mouse Model (GEMM) Core Unit. Briefly, a mixture of the 40 ng/µL 3X NLS SpCas9 protein (SJCRH Protein Production Core), 20 ng/µL chemically modified sgRNA (CAGE1103.Smo.g2 – 5’-UUGUAGGCUACAAGAACUAU-3’; Synthego), and 10 ng/µL donor ssODN(CAGE1103.g2.sense.ssODN 5’ agatggagactccgtgagtggcatctgttttgtaggctacGCCaactatcggtaccgtgctggctttgtcctggccccaattggcc 3’; IDT AltR modification) was injected into fertilized zygotes as previously described^83^. Animals were genotyped by targeted deep sequencing using CAGE1103.Smo.F – 5’ ctacacgacgctcttccgatctCACCTGCTCACGTGGTCACTCCCCT 3’ and CAGE1103.Smo. R – 5’ cagacgtgtgctcttccgatctAGAGGAAGGCAGTGGAGCTGGAAGC 3’ at the Center for Advanced Genome Engineering (SJCRH); the resulting sequences were analyzed using CRIS.py as described^84^. Animals positive for the desired K399A modification were backcrossed to C57BL/6 J mice and then bred to homozygosity.

### Embryo Immunoprecipitation

Embryos were dissected at E13.5-E14.5, washed in PBS, transferred to microfuge tubes, flash frozen in liquid nitrogen, and stored at -80°C during genotyping analysis (TransnetYX). Embryos were dissociated in 600 μL TBS with 1X PIC and 1 mM PMSF (embryo wash buffer) using a KIMBLE Motorized Pellet Pestle (Daigger Scientific). The resulting cell suspension was pelleted at 300 × *g* for 5 min, washed twice in embryo wash buffer, and resuspended in 600 μL lysis buffer (1% NP-40, 50 mM Tris-HCl pH 7.9, 150 mM NaCl, 5 mM NaF, 1 mM PMSF, 5% glycerol, 1 mM EDTA, 2 mM MgCl_2_, and 1X PIC (Pierce)). As a control, 1 × 10^6^ *Smo*^-/-^ MEFs were seeded in 10-cm dishes and transfected the following day with 6 μg pcDNA3 vector or pcDNA3-SMO-WT using Lipofectamine 3000 (Thermo Fisher Scientific) according to manufacturer’s protocols. Cells were lysed ~48 h post transfection in lysis buffer. Each sample (embryo and MEF controls) was incubated on ice for 30 min with 25U Benzonase nuclease (Sigma-Aldrich). Cells were needled 10X each with 18G, 21G, and then 23G needles. Samples were centrifuged at 2000 × g for 10 min at 4 °C. Supernatants were transferred to fresh tubes and then centrifuged again prior to protein concentration determination through BCA assay (Thermo Fisher Scientific) following manufacturer’s protocols.

For immunoprecipitation, lysates were pre-cleared with 20 μL equilibrated protein A/G plus agarose beads (50% slurry, SCBT) and 30 μL equilibrated protein L agarose beads (50% slurry, SCBT) for 1 hour, rotating at 4 °C. Cleared lysates were incubated with 2 μg mouse anti-SMO E5 (SCBT) or 0.25 μg mouse IgG control (Jackson ImmunoResearch Laboratories), rotating at 4 °C for 16 h. Immune complexes were collected by addition of 25 μL protein A/G agarose, rotating for 1 hour at 4 °C. Beads were washed 3x in 1% NP40 lysis buffer by centrifugation at 800 × *g* for 1 min at 4 °C. SMO protein was eluted in 5x sample buffer (10% w/v SDS, 50 mM DTT, 20% v/v glycerol, 0.2 M Tris-HCl pH 6.8, 0.05% w/v Bromophenol blue) for 10 min then diluted to 2.5x with 1% NP40 lysis buffer. Samples were centrifuged at 1000 x g for 1 min and eluates were collected. Immunoblotting was performed as described above. Anti-SMO was detected using HRP-conjugated rat anti-mouse light chain (Southern Biotech).

### Mouse immunohistochemistry

Wild-type C57BL/6 J (JAX#000664) and *Smo*^*K399A*^ mutant embryos on a C57BL/6 background were collected and processed for immunohistochemistry at E9.5. Pregnant dams were euthanized, uterine horns were removed, and embryos were dissected in 1X PBS before being rinsed three times. Whole embryos were imaged using a Stereo Microscope (Leica), and somites were counted. The embryos were then fixed in 4% PFA at room temperature for 1 hour, rinsed three times with 1X PBS, and cryo-protected in 30% sucrose at 4 °C overnight. The next day, embryos were frozen in O.C.T. Compound (Tissue-Tek) on dry ice. Transverse sections of the embryos were cut at a thickness of 10 µm using a Leica Microm CM1950 cryostat. The sections were briefly dried, washed in 1X TBST, and blocked with a buffer containing 2% BSA, 1% goat serum, and 0.1% Triton-X-100 in 1X PBS. Antibodies diluted in the blocking buffer were applied to the sections and incubated overnight at room temperature in a humidified chamber. The following antibodies and their dilutions were used: mouse anti-PAX6 (1:10, DSHB), rabbit anti-OLIG2 (1:300, Millipore), rabbit anti-FOXA2 (1:250, Abcam), and rabbit anti-NKX2.2 (1:200, Novus Bio). After removing the primary antibodies, the sections were washed three times with 1X TBST, then incubated with secondary antibodies (Invitrogen) at a 1:500 dilution for 3 hours. The sections were washed three times with 1X TBST, rinsed with tap water, dried, and mounted with cover slips using ProLong Gold mounting media with DAPI. Imaging was performed using a Leica DMi8 widefield microscope and processed with LAS X software. A minimum of five cardiac level sections each from at least three embryos per genotype were analyzed. Analyses were performed on somite-matched pairs of embryos ranging in somites from 25-29.

All images analyzed for neural tube patterning were from the cardiac region adjacent to somites 2 and 3. The branchial pouches, aortic sac, and the size and morphology of the atria and ventricles were used as landmarks to identify and align the transverse sections at the cardiac level. Reference images g and h from the Kaufman Atlas of Mouse Development Plate 19b illustrate the cardiac level regions analyzed for expression domain quantifications^85^.

### Neural tube progenitor domain quantification

Using the polygon selection tool in ImageJ, both the neural tube area and the indicated progenitor domain areas were determined. The expression domain areas were then normalized to the total neural tube area for each section analyzed^21^. Analysis was performed on three to five embryos per genotype, with 4–6 sections examined per embryo.

### RNA sequencing

*Smo*^*K399A/+*^ females were crossed with *Smo*^*K399A/+*^ males to generate litters of E9.5 *Smo*^*+/+*^ and *Smo*^*K399A/K399A*^ embryos. Multiple litters were harvested, and pairs of littermates were used to maximize genetic variability (*n* = 4 embryos per genotype analyzed). Pregnant dams were harvested, embryos were dissected, and each embryo was placed in a 1.5 mL Eppendorf tube, flash frozen in liquid nitrogen, and then stored at −80 °C while genotyping was performed. Once four pairs of embryos were generated, all samples were purified at the same time. Each whole embryo was homogenized, and RNA was extracted using a RNeasy Micro Kit (Qiagen) following the manufacturer’s protocol. RNA sequencing libraries for each sample were prepared with 1 mg total RNA by using the Illumina TruSeq RNA Sample Prep v2 Kit per the manufacturer’s instructions, and sequencing was completed on the Illumina NovaSeq 6000. The 100-bp paired-end reads were trimmed, filtered for quality (Phred-like Q20 or greater) and length (50-bp or longer), and aligned to a mouse reference sequence GRCm38 (UCSC mm10) by using CLC Genomics Workbench v12.0.1 (Qiagen). The transcript per million (TPM) counts were generated from the CLC RNA-Seq Analysis tool. The differential gene expression analysis was performed by using a non-parametric analysis of variance (ANOVA) using the Kruskal-Wallis and Dunn’s tests on log-transformed TPM between four biological replicates from each of two experimental groups, implemented in Partek Genomics Suite v7.0 software (Partek Inc.). The gene set enrichment and pathway analyses were performed using GSEA^86^.

### Quantitative reverse transcriptase polymerase chain reaction (qRT-PCR)

Total RNA was extracted from cells using the TRIzol™ (Invitrogen) method according to the manufacturer’s protocols. One thousand nanograms of RNA was used to synthesize complementary DNA (cDNA) using the High-Capacity cDNA Reverse Transcription Kit (Applied Biosystems). qRT-PCR reactions were performed on a QuantStudio 7 Flex PCR machine using PowerUp Sybr Green Master Mix (Applied Biosystems). Corresponding changes in the expression levels of *Gli1* were calculated using the ΔΔCt method relative to housekeeping genes *Ppia* or *Btf3*^18^.

Primer sequences (IDT):

*Gli1* F: GGTGCTGCCTATAGCCAGTGTCCTC

*Gli1* R: GTGCCAATCCGGTGGAGTCAGACCC

*Pdgfra* F: GCAGTTGCCTTACGACTCCAGA

*Pdgfra* R: GGTTTGAGCATCTTCACAGCCAC

*Btf3* F: GAA CAA CAT CTC TGG TAT TGA AGA

*Btf3* R: AAT GGT GAA GGT GTT TGC TG

*Ppia* F: AGC ACT GGA GAG AAA GGA TT

*Ppia* R: ATT ATG GCG TGT AAA GTC ACC A

### PDGFRA^+^ cell isolation from embryonic lungs

Lungs from E18.5 and P0 *Smo*^*K399A/K399A*^ and *Smo*^*+/+*^ littermates were harvested, and lung tissue was placed in pre-warmed DMEM with collagenase IV (310 U/mL, Worthington). Lungs were minced using scissors and incubated at 37 °C for 1 hour. Following digestion, an equal volume of DMEM supplemented with 2% FBS was added, and the samples were filtered through a 70-µm cell strainer. The resulting cell suspension was centrifuged at 200 x g for 5 min at 4 °C, and the pellet was treated with RBC lysis buffer (Invitrogen) for 5 minutes at room temperature. After a second centrifugation, cells were resuspended in purification buffer (PBS with 0.2% BSA and 2% FBS) at a concentration of 1 × 10^6^/mL. The cell suspension was incubated with biotin-conjugated PDGFRA antibody (1:100, R&D Systems) at room temperature for 10 min, followed by the addition of streptavidin magnetic beads (Pierce). This cell suspension was then incubated at 4 °C for 1 hour on a rotator. After incubation, the streptavidin beads were washed three times for 5 min each with wash buffer (PBS with 2% FBS) at room temperature. TRIzol™ was added directly to the beads, and RNA was extracted and processed as stated above. PDGFRA-positive cell enrichment was validated by analyzing *Pdgfra* expression levels in RNA isolated from pre-conjugated cell suspension compared to post-purification. *Gli1* expression levels were analyzed in enriched PDGFRA*+* lung cells.

### Embryo histology for cardiac phenotyping

*Smo*^*+/+*^*, Smo*^*K399A/+*^, and *Smo*^*K399A/K399A*^ E14.5 embryos were dissected and fixed in 4% paraformaldehyde overnight at 4 °C. Embryo trunk tissues were then dehydrated, embedded in paraffin, and sectioned at a thickness of 5 µm prior to hematoxylin and eosin (H&E) staining by the Human Tissue Resource Center at The University of Chicago facility. A genotype-blind approach was used for cardiac phenotyping, and images were taken using a Leica DM2500 optical microscope.

To analyze lung morphology in adult mice, lungs were dissected and embedded in paraffin blocks, sectioned, and stained with H&E for histology imaging. The airspace area within randomly selected lung sections was calculated using FIJI/ImageJ. A threshold on images was set to segment the void spaces, and the % area was calculated. Two randomly chosen areas from each lung section were analyzed. Sections from five mice/genotype were included in analysis.

### Lung function measurement

#### Assessment of airway hyperresponsiveness by whole body plethysmography (WBP)

Unanesthetized mice were randomly placed into an 8-chamber plethysmograph and allowed to acclimate for 30 min before collecting measurements. To evaluate the change in airway physiological parameters, the pressure within each of the plethysmograph chambers was compared to a reference chamber. Following baseline measurements, breathing frequency, tidal volume, inspiratory, and expiratory events were simultaneously recorded in each chamber at intervals of five minutes for a duration of 45 min. The enhanced pause (Penh) parameter was calculated using Buxco Finepointe software (Data Sciences International).

#### Assessment of lung resistance and dynamic lung compliance in response to Methacholine inhalation

Lung resistance (RI) and dynamic lung compliance to increasing doses of nebulized methacholine were measured using the Buxco Finepointe Resistance and Compliance system (Data Sciences International). Anesthetized mice were mechanically ventilated through endotracheal intubation. Gradient doses of methacholine (MeCH: 3.125, 6.25, 12.5, and 25 mg/mL) were nebulized to the heated chamber. Airway resistance and dynamic lung compliance were measured over 3 min with a 1 min recovery period at each MeCH dose. Results were collected and quantified using Buxco Finepointe software (Data Sciences International).

### Saturated phosphatidylcholine (SatPC) analysis

Lung tissue was collected from fetal (E18.5-19.5) and newborn (P0) mice and flash frozen. Lipids were extracted from homogenized lung tissue by the Bligh and Dyer method^87^. SatPC was isolated using the osmium tetroxide-based method of Mason et al^88^. and quantitated by phosphorus measurement, as we have previously described^89^. SatPC levels reported here represent the total amount of lung SatPC in the lung normalized to lung weight.

### Magnetic resonance imaging

Magnetic resonance imaging (MRI) was performed with a Bruker Clinscan 7 T MRI system (Bruker Biospin MRI GmbH). Prior to imaging, mice were anesthetized in a chamber with 3% Isoflurane in oxygen at a flow rate of 1 L/min. Mice were maintained in an anesthetized state with 1%–2% Isoflurane via a nose cone delivery system with a flow rate of 1 L/min. Thermal support was provided to the animals using a heated bed with warm water circulation, and a physiological monitoring system tracked their breathing rate. During the MRI procedure, a mouse brain volume coil was positioned over the mouse head, with the mouse placed inside a 72-mm transmit/receive coil.

The imaging protocol included T2-weighted turbo spin echo sequences in horizontal (TR/TE = 3080/40 ms, matrix size = 256 × 256, field of view = 36 mm×36 mm, slice thickness = 0.5 mm, number of slices = 20), coronal (TR/TE = 4760/42 ms, matrix size = 192 × 192, field of view = 15 mm×15 mm, slice thickness = 0.5 mm, number of slices = 30), and sagittal (TR/TE = 3580/39 ms, matrix size = 128 × 256, field of view = 16 mm × 32 mm, slice thickness = 0.5 mm, number of slices = 24) orientations.

MRI image analysis was performed using 3D Slicer (Surgical Planning Laboratory). Coronal images were segmented by manually outlining regions of interest to delineate the entire brain. This segmentation process was repeated across all slices to cover the entire brain volume. The brain volume was then calculated based on voxel dimensions. Similarly, a separate segmentation was created for the cerebellum, and its volume was determined using the same method.

### Computed tomography

Computed tomography (CT) scans were conducted using a Siemens Inveon PET/CT system at an isotropic resolution of 45 µm. Mice were anesthetized and positioned as described above. The total duration of the scan was 16 min and 34 sec. The CT images were analyzed using Inveon Research Workplace software (Siemens). Measurements of L1 to L10 dimensions were taken based on predetermined measurement locations.

### Statistical methods

Sample sizes for functional assays were selected to be consistent with previously published work from our lab and others^18,21,22,28^. Sample size was considered in all statistical analyses. Statistical methods were applied as per the instructions of the SJCRH Biostatistics Department. Sample sizes for animal studies were selected to be consistent with previous work from the lab^21,77,83^ and/or calculated by power analysis to ensure ~80% power^90^.

All statistical analyses were performed using GraphPad Prism v10-11.0.2, unless otherwise specified. Student’s t-test was carried out to compare statistical significance between two means, and unpaired non-parametric one-way ANOVA or two-way ANOVA was performed for comparison of multiple means, as indicated. Fisher’s exact test was performed to determine significance between expected and observed Mendelian ratios between different genotypes, and Overall Chi-square analysis was performed for correlation of heart defects to the mouse genotypes.

For the AA bead capture assay, the percentage of SMO protein captured on beads was quantified by comparing densitometry of SMO captured on beads following various treatments to the densitometry of SMO bound to beads in the absence of additional chemical treatments. The area under the curve (AUC) for percentage of bound SMO across dose levels was calculated for each biological replicate using the trapezoidal method with a minimum of three biological replicates per condition. Differences in the AUC and percentage of SMO protein were assessed using one-way ANOVA. When significant differences were identified by the overall F-test, post hoc pairwise comparisons were performed. Statistical analyses were performed under the assumption of normality. Statistical tests were two-sided.

Statistics for ciliary quantification were calculated from at least two independent experiments with at least 50 cilia per condition per experiment. All quantified data are presented as mean ± SD, with *p* < 0.05 considered statistically significant. Significance depicted as *p < 0.05, **p < 0.01, ***p < 0.001, ****p < 0.0001 and ns = not significant.

### Materials availability

Plasmids generated in this study will be deposited to Addgene at the time of publication. Cell lines and mouse models will be available upon request to the lead contact pursuant to SJCRH material transfer agreements.

### Reporting summary

Further information on research design is available in the Nature Portfolio Reporting Summary linked to this article.

## Supplementary information

Supplementary Information

Peer Review file

Description of Additional Supplementary Files

Supplementary Movie 1

Reporting Summary

## Source data

Source Data

## Data Availability

The RNAseq dataset was deposited into GEO with an accession number of GSE283994. Raw data will be available upon request to the corresponding author, Stacey.Ogden@stjude.org. Source data are provided with this paper.

## References

[1] Ingham, P. W. Hedgehog signaling. *Curr. Top. Dev. Biol.* **149**, 1–58 (2022). 35606054 10.1016/bs.ctdb.2022.04.003

[2] Zhang, Y. & Beachy, P. A. Cellular and molecular mechanisms of Hedgehog signalling. *Nat. Rev. Mol. Cell Biol.* **24**, 668–687 (2023). 36932157 10.1038/s41580-023-00591-1 PMC12140928

[3] Chiang, C. et al. Cyclopia and defective axial patterning in mice lacking Sonic hedgehog gene function. *Nature* **383**, 407–413 (1996). 8837770 10.1038/383407a0

[4] Kugler, M. C., Joyner, A. L., Loomis, C. A. & Munger, J. S. Sonic hedgehog signaling in the lung. From development to disease. *Am. J. Respir. Cell Mol. Biol.* **52**, 1–13 (2015). 25068457 10.1165/rcmb.2014-0132TR PMC4370254

[5] Morrisey, E. E. & Hogan, B. L. Preparing for the first breath: genetic and cellular mechanisms in lung development. *Dev. Cell* **18**, 8–23 (2010). 20152174 10.1016/j.devcel.2009.12.010 PMC3736813

[6] Rowton, M. et al. Hedgehog signaling activates a mammalian heterochronic gene regulatory network controlling differentiation timing across lineages. *Dev. Cell* **57**, 2181–2203.e2189 (2022). 36108627 10.1016/j.devcel.2022.08.009 PMC10506397

[7] Zhang, K., Yao, E., Aung, T. & Chuang, P. T. The alveolus: Our current knowledge of how the gas exchange unit of the lung is constructed and repaired. *Curr. Top. Dev. Biol.* **159**, 59–129 (2024). 38729684 10.1016/bs.ctdb.2024.01.002

[8] Goetz, S. C. & Anderson, K. V. The primary cilium: a signalling centre during vertebrate development. *Nat. Rev. Genet* **11**, 331–344 (2010). 20395968 10.1038/nrg2774 PMC3121168

[9] Mukhopadhyay, S. & Rohatgi, R. G-protein-coupled receptors, Hedgehog signaling and primary cilia. *Semin. Cell Dev. Biol.*10.1016/j.semcdb.2014.05.002. 10.1016/j.semcdb.2014.05.002 PMC4130902 24845016

[10] Kinnebrew, M. et al. Patched 1 regulates Smoothened by controlling sterol binding to its extracellular cysteine-rich domain. *Sci. Adv.* **8**, eabm5563 (2022). 35658032 10.1126/sciadv.abm5563 PMC9166294

[11] Tukachinsky, H., Petrov, K., Watanabe, M. & Salic, A. Mechanism of inhibition of the tumor suppressor Patched by Sonic Hedgehog. *Proc. Natl. Acad. Sci. USA* **113**, E5866–E5875 (2016). 27647915 10.1073/pnas.1606719113 PMC5056095

[12] Zhang, Y. et al. StructurAl Basis For Cholesterol Transport-like Activity Of The Hedgehog Receptor Patched. *Cell* **175**, 1352–1364.e1314 (2018). 30415841 10.1016/j.cell.2018.10.026 PMC6326742

[13] Niewiadomski, P. et al. Gli protein activity is controlled by multisite phosphorylation in vertebrate Hedgehog signaling. *Cell Rep.* **6**, 168–181 (2014). 24373970 10.1016/j.celrep.2013.12.003 PMC3915062

[14] Denef, N., Neubuser, D., Perez, L. & Cohen, S. M. Hedgehog induces opposite changes in turnover and subcellular localization of patched and smoothened. *Cell* **102**, 521–531 (2000). 10966113 10.1016/s0092-8674(00)00056-8

[15] Arensdorf, A. M., Marada, S. & Ogden, S. K. Smoothened regulation: a tale of two signals. *Trends Pharm. Sci.* **37**, 62–72 (2016). 26432668 10.1016/j.tips.2015.09.001 PMC4593303

[16] Tukachinsky, H., Lopez, L. V. & Salic, A. A mechanism for vertebrate Hedgehog signaling: recruitment to cilia and dissociation of SuFu-Gli protein complexes. *J. Cell Biol.* **191**, 415–428 (2010). 20956384 10.1083/jcb.201004108 PMC2958481

[17] Polizio, A. H., Chinchilla, P., Chen, X., Manning, D. R. & Riobo, N. A. Sonic Hedgehog activates the GTPases Rac1 and RhoA in a Gli-independent manner through coupling of smoothened to Gi proteins. *Sci. Signal* **4**, pt7 (2011). 22114142 10.1126/scisignal.2002396 PMC5811764

[18] Arensdorf, A. M. et al. Sonic Hedgehog activates Phospholipase A2 to enhance smoothened ciliary translocation. *Cell Rep.* **19**, 2074–2087 (2017). 28591579 10.1016/j.celrep.2017.05.033 PMC5520665

[19] Bijlsma, M. F., Borensztajn, K. S., Roelink, H., Peppelenbosch, M. P. & Spek, C. A. Sonic hedgehog induces transcription-independent cytoskeletal rearrangement and migration regulated by arachidonate metabolites. *Cell. Signal.* **19**, 2596–2604 (2007). 17884337 10.1016/j.cellsig.2007.08.011

[20] Belgacem, Y. H. & Borodinsky, L. N. Sonic hedgehog signaling is decoded by calcium spike activity in the developing spinal cord. *Proc. Natl. Acad. Sci. USA* **108**, 4482–4487 (2011). 21368195 10.1073/pnas.1018217108 PMC3060219

[21] Ansari, S. S. et al. Sonic Hedgehog activates prostaglandin signaling to stabilize primary cilium length. *J. Cell Biol*. **223**, 10.1083/jcb.202306002. 10.1083/jcb.202306002 PMC11166601 38856684

[22] Luchetti, G. et al. Cholesterol activates the G-protein coupled receptor Smoothened to promote Hedgehog signaling. *Elife* **5**, 10.7554/eLife.20304. 10.7554/eLife.20304 PMC5123864 27705744

[23] Huang, P. et al. Cellular cholesterol directly activates smoothened in Hedgehog signaling. *Cell* **166**, 1176–1187.e1114 (2016). 27545348 10.1016/j.cell.2016.08.003 PMC5035717

[24] Myers, B. R. et al. Hedgehog pathway modulation by multiple lipid binding sites on the smoothened effector of signal response. *Dev. Cell* **26**, 346–357 (2013). 23954590 10.1016/j.devcel.2013.07.015 PMC4196939

[25] Qi, X., Friedberg, L., De Bose-Boyd, R., Long, T. & Li, X. Sterols in an intramolecular channel of Smoothened mediate Hedgehog signaling. *Nat. Chem. Biol.* **16**, 1368–1375 (2020). 32929279 10.1038/s41589-020-0646-2 PMC7669734

[26] Qi, X. et al. Cryo-EM structure of oxysterol-bound human Smoothened coupled to a heterotrimeric G(i). *Nature* **571**, 279–283 (2019). 31168089 10.1038/s41586-019-1286-0 PMC6777001

[27] Byrne, E. F. et al. Structural basis of Smoothened regulation by its extracellular domains. *Nature* **535**, 517–522 (2016). 27437577 10.1038/nature18934 PMC4970916

[28] Raleigh, D. R. et al. Cilia-associated Oxysterols activate smoothened. *Mol. Cell* **72**, 316–327 e315 (2018). 30340023 10.1016/j.molcel.2018.08.034 PMC6503851

[29] Nachtergaele, S. et al. Oxysterols are allosteric activators of the oncoprotein Smoothened. *Nat. Chem. Biol.* **8**, 211–220 (2012). 22231273 10.1038/nchembio.765 PMC3262054

[30] Nedelcu, D., Liu, J., Xu, Y., Jao, C. & Salic, A. Oxysterol binding to the extracellular domain of Smoothened in Hedgehog signaling. *Nat. Chem. Biol.* **9**, 557–564 (2013). 23831757 10.1038/nchembio.1290 PMC3749252

[31] Nachtergaele, S. et al. Structure and function of the Smoothened extracellular domain in vertebrate Hedgehog signaling. *Elife* **2**, e01340 (2013). 24171105 10.7554/eLife.01340 PMC3809587

[32] Thal, D. M., Glukhova, A., Sexton, P. M. & Christopoulos, A. Structural insights into G-protein-coupled receptor allostery. *Nature* **559**, 45–53 (2018). 29973731 10.1038/s41586-018-0259-z

[33] Duvernay, M. T., Matafonov, A., Lindsley, C. W. & Hamm, H. E. Platelet lipidomic profiling: novel insight into cytosolic phospholipase A2alpha activity and its role in human platelet activation. *Biochemistry* **54**, 5578–5588 (2015). 26295742 10.1021/acs.biochem.5b00549 PMC7748375

[34] Wang, C. et al. Structural basis for Smoothened receptor modulation and chemoresistance to anticancer drugs. *Nat. Commun.* **5**, 4355 (2014). 25008467 10.1038/ncomms5355 PMC4198951

[35] Sharpe, H. J., Wang, W., Hannoush, R. N. & de Sauvage, F. J. Regulation of the oncoprotein Smoothened by small molecules. *Nat. Chem. Biol.* **11**, 246–255 (2015). 25785427 10.1038/nchembio.1776

[36] Kinnebrew, M. et al. Patched 1 reduces the accessibility of cholesterol in the outer leaflet of membranes. *Elife* **10**, 10.7554/eLife.70504. 10.7554/eLife.70504 PMC8654371 34698632

[37] Maerz, L. D. et al. Pharmacological cholesterol depletion disturbs ciliogenesis and ciliary function in developing zebrafish. *Commun. Biol.* **2**, 31 (2019). 30729178 10.1038/s42003-018-0272-7 PMC6351647

[38] Pike, L. J., Han, X., Chung, K. N. & Gross, R. W. Lipid rafts are enriched in arachidonic acid and plasmenylethanolamine and their composition is independent of caveolin-1 expression: a quantitative electrospray ionization/mass spectrometric analysis. *Biochemistry* **41**, 2075–2088 (2002). 11827555 10.1021/bi0156557

[39] Zidovetzki, R. & Levitan, I. Use of cyclodextrins to manipulate plasma membrane cholesterol content: evidence, misconceptions and control strategies. *Biochim. Biophys. Acta* **1768**, 1311–1324 (2007). 17493580 10.1016/j.bbamem.2007.03.026 PMC1948080

[40] Brunaldi, K., Huang, N. & Hamilton, J. A. Fatty acids are rapidly delivered to and extracted from membranes by methyl-beta-cyclodextrin. *J. Lipid Res.* **51**, 120–131 (2010). 19625735 10.1194/jlr.M900200-JLR200 PMC2789772

[41] Huang, P. et al. Structural basis of smoothened activation in Hedgehog Signaling. *Cell* **174**, 312–324.e316 (2018). 29804838 10.1016/j.cell.2018.04.029 PMC6046275

[42] Wright, W. C. et al. Clobetasol Propionate Is a Heme-mediated selective inhibitor of human Cytochrome P450 3A5. *J. Med Chem.* **63**, 1415–1433 (2020). 31965799 10.1021/acs.jmedchem.9b02067 PMC7087482

[43] Deshpande, I. et al. Smoothened stimulation by membrane sterols drives Hedgehog pathway activity. *Nature* **571**, 284–288 (2019). 31263273 10.1038/s41586-019-1355-4 PMC6709672

[44] Dijkgraaf, G. J. et al. Small molecule inhibition of GDC-0449 refractory smoothened mutants and downstream mechanisms of drug resistance. *Cancer Res.* **71**, 435–444 (2011). 21123452 10.1158/0008-5472.CAN-10-2876

[45] Marada, S. et al. Functional Divergence in the Role of N-Linked Glycosylation in Smoothened Signaling. *PLoS Genet.* **11**, e1005473 (2015). 26291458 10.1371/journal.pgen.1005473 PMC4546403

[46] Wang, Y., Zhou, Z., Walsh, C. T. & McMahon, A. P. Selective translocation of intracellular Smoothened to the primary cilium in response to Hedgehog pathway modulation. *Proc. Natl. Acad. Sci. USA* **106**, 2623–2628 (2009). 19196978 10.1073/pnas.0812110106 PMC2650314

[47] Xie, J. et al. Activating Smoothened mutations in sporadic basal-cell carcinoma. *Nature* **391**, 90–92 (1998). 9422511 10.1038/34201

[48] Hoffmeister, H. et al. Polycystin-2 takes different routes to the somatic and ciliary plasma membrane. *J. Cell Biol.* **192**, 631–645 (2011). 21321097 10.1083/jcb.201007050 PMC3044124

[49] Bonventre, J. V. et al. Reduced fertility and postischaemic brain injury in mice deficient in cytosolic phospholipase A2. *Nature* **390**, 622–625 (1997). 9403693 10.1038/37635

[50] Ingham, P. W. & McMahon, A. P. Hedgehog signaling in animal development: paradigms and principles. *Genes Dev.* **15**, 3059–3087 (2001). 11731473 10.1101/gad.938601

[51] Dessaud, E., McMahon, A. P. & Briscoe, J. Pattern formation in the vertebrate neural tube: a sonic hedgehog morphogen-regulated transcriptional network. *Development* **135**, 2489–2503 (2008). 18621990 10.1242/dev.009324

[52] Bellusci, S. et al. Involvement of Sonic hedgehog (Shh) in mouse embryonic lung growth and morphogenesis. *Development* **124**, 53–63 (1997). 9006067 10.1242/dev.124.1.53

[53] Goddeeris, M. M., Schwartz, R., Klingensmith, J. & Meyers, E. N. Independent requirements for Hedgehog signaling by both the anterior heart field and neural crest cells for outflow tract development. *Development* **134**, 1593–1604 (2007). 17344228 10.1242/dev.02824

[54] Dyer, L. A. & Kirby, M. L. Sonic hedgehog maintains proliferation in secondary heart field progenitors and is required for normal arterial pole formation. *Dev. Biol.* **330**, 305–317 (2009). 19361493 10.1016/j.ydbio.2009.03.028 PMC2810612

[55] Liu, L. et al. Hedgehog signaling in neonatal and adult lung. *Am. J. Respir. Cell Mol. Biol.* **48**, 703–710 (2013). 23371063 10.1165/rcmb.2012-0347OC PMC3727871

[56] Klena, N. T., Gibbs, B. C. & Lo, C. W. Cilia and Ciliopathies in Congenital Heart Disease. *Cold Spring Harb. Perspect. Biol*. **9**, 10.1101/cshperspect.a028266. 10.1101/cshperspect.a028266 PMC5538412 28159874

[57] Koefoed, K., Veland, I. R., Pedersen, L. B., Larsen, L. A. & Christensen, S. T. Cilia and coordination of signaling networks during heart development. *Organogenesis* **10**, 108–125 (2014). 24345806 10.4161/org.27483 PMC4049888

[58] Burnicka-Turek, O. et al. Cilia gene mutations cause atrioventricular septal defects by multiple mechanisms. *Hum. Mol. Genet.* **25**, 3011–3028 (2016). 27340223 10.1093/hmg/ddw155 PMC5181596

[59] Xie, L. et al. Tbx5-hedgehog molecular networks are essential in the second heart field for atrial septation. *Dev. Cell* **23**, 280–291 (2012). 22898775 10.1016/j.devcel.2012.06.006 PMC3912192

[60] Ng, W. H. et al. Recapitulating human cardio-pulmonary co-development using simultaneous multilineage differentiation of pluripotent stem cells. *Elife* **11**, 10.7554/eLife.67872. 10.7554/eLife.67872 PMC8846595 35018887

[61] Zhang, K. et al. A functional circuit formed by the autonomic nerves and myofibroblasts controls mammalian alveolar formation for gas exchange. *Dev. Cell* **57**, 1566–1581.e1567 (2022). 35714603 10.1016/j.devcel.2022.05.021 PMC9308505

[62] Yie, T. A. et al. Hedgehog and platelet-derived growth factor signaling intersect during postnatal lung development. *Am. J. Respir. Cell Mol. Biol.* **68**, 523–536 (2023). 36693140 10.1165/rcmb.2022-0269OC PMC10174164

[63] Stevens, P. A., Wright, J. R. & Clements, J. A. Changes in quantity, composition, and surface activity of alveolar surfactant at birth. *J. Appl Physiol. (1985)* **63**, 1049–1057 (1987). 3654453 10.1152/jappl.1987.63.3.1049

[64] Han, S. & Mallampalli, R. K. The role of surfactant in lung disease and host defense against pulmonary infections. *Ann. Am. Thorac. Soc.* **12**, 765–774 (2015). 25742123 10.1513/AnnalsATS.201411-507FR PMC4418337

[65] Liu, L. Regulation of lung surfactant secretion by phospholipase A2. *J. Cell Biochem.* **72**, 103–110 (1999). 10025671

[66] Baybutt, R. C., Smith, J. E., Gillespie, M. N., Newcomb, T. G. & Yeh, Y. Y. Arachidonic acid and eicosapentaenoic acid stimulate type II pneumocyte surfactant secretion. *Lipids* **29**, 535–539 (1994). 7990659 10.1007/BF02536624

[67] DeLorme, M. P. & Moss, O. R. Pulmonary function assessment by whole-body plethysmography in restrained versus unrestrained mice. *J. Pharm. Toxicol. Methods* **47**, 1–10 (2002). 10.1016/s1056-8719(02)00191-0 12387933

[68] Boyd, D. F. et al. Exuberant fibroblast activity compromises lung function via ADAMTS4. *Nature* **587**, 466–471 (2020). 33116313 10.1038/s41586-020-2877-5 PMC7883627

[69] Zhang, X. M., Ramalho-Santos, M. & McMahon, A. P. Smoothened mutants reveal redundant roles for Shh and Ihh signaling including regulation of L/R symmetry by the mouse node. *Cell* **106**, 781–792 (2001). 11517919

[70] Placzek, M. & Briscoe, J. Sonic hedgehog in vertebrate neural tube development. *Int J. Dev. Biol.* **62**, 225–234 (2018). 29616731 10.1387/ijdb.170293jb

[71] Hoffmann, A. D., Peterson, M. A., Friedland-Little, J. M., Anderson, S. A. & Moskowitz, I. P. Sonic Hedgehog is required in pulmonary endoderm for atrial septation. *Development* **136**, 1761–1770 (2009). 19369393 10.1242/dev.034157 PMC2673765

[72] Lescroart, F. et al. Early lineage restriction in temporally distinct populations of Mesp1 progenitors during mammalian heart development. *Nat. Cell Biol.* **16**, 829–840 (2014). 25150979 10.1038/ncb3024 PMC6984965

[73] Kugler, M. C. et al. Sonic Hedgehog Signaling Regulates Myofibroblast Function During Alveolar Septum Formation In Murine Postnatal Lung. *Am. J. Respir. Cell Mol. Biol.* **57**, 280–293 (2017). 28379718 10.1165/rcmb.2016-0268OC PMC5625221

[74] Zhang, X. et al. Crystal structure of a multi-domain human smoothened receptor in complex with a super stabilizing ligand. *Nat. Commun.* **8**, 15383 (2017). 28513578 10.1038/ncomms15383 PMC5442369

[75] Kong, J. H. et al. A membrane-tethered ubiquitination pathway regulates Hedgehog signaling and heart development. *Dev. Cell* **55**, 432–449.e412 (2020). 32966817 10.1016/j.devcel.2020.08.012 PMC7686252

[76] Pusapati, G. V. et al. CRISPR screens uncover genes that regulate target cell sensitivity to the morphogen Sonic Hedgehog. *Dev. Cell* **44**, 113–129.e118 (2018). 29290584 10.1016/j.devcel.2017.12.003 PMC5792066

[77] Hall, E. T. et al. Cytoneme delivery of Sonic Hedgehog from ligand-producing cells requires Myosin 10 and a Dispatched-BOC/CDON co-receptor complex. *Elife* **10**, 10.7554/eLife.61432. 10.7554/eLife.61432 PMC7968926 33570491

[78] Maity, T., Fuse, N. & Beachy, P. A. Molecular mechanisms of Sonic hedgehog mutant effects in holoprosencephaly. *Proc. Natl. Acad. Sci. USA* **102**, 17026–17031 (2005). 16282375 10.1073/pnas.0507848102 PMC1282174

[79] Ye, F. et al. Single molecule imaging reveals a major role for diffusion in the exploration of ciliary space by signaling receptors. *Elife* **2**, e00654 (2013). 23930224 10.7554/eLife.00654 PMC3736543

[80] Larkins, C. E., Aviles, G. D., East, M. P., Kahn, R. A. & Caspary, T. Arl13b regulates ciliogenesis and the dynamic localization of Shh signaling proteins. *Mol. Biol. Cell* **22**, 4694–4703 (2011). 21976698 10.1091/mbc.E10-12-0994 PMC3226485

[81] Harder, E. et al. OPLS3: a force field providing broad coverage of drug-like small molecules and proteins. *J. Chem. Theory Comput.* **12**, 281–296 (2016). 26584231 10.1021/acs.jctc.5b00864

[82] Friesner, R. A. et al. Extra precision glide: docking and scoring incorporating a model of hydrophobic enclosure for protein-ligand complexes. *J. Med. Chem.* **49**, 6177–6196 (2006). 17034125 10.1021/jm051256o

[83] Hall, E. T. et al. Cytoneme signaling provides essential contributions to mammalian tissue patterning. *Cell* **187**, 276–293.e223 (2024). 38171360 10.1016/j.cell.2023.12.003 PMC10842732

[84] Narina, S., Connelly, J. P. & Pruett-Miller, S. M. High-throughput analysis of CRISPR-Cas9 editing outcomes in cell and animal models using CRIS.py. *Methods Mol. Biol.* **2631**, 155–182 (2023). 36995667 10.1007/978-1-0716-2990-1_6 PMC10427880

[85] Graham, E. et al. The atlas of mouse development eHistology resource. *Development* **142**, 1909–1911 (2015). 26015534 10.1242/dev.124917

[86] Subramanian, A. et al. Gene set enrichment analysis: a knowledge-based approach for interpreting genome-wide expression profiles. *Proc. Natl. Acad. Sci. USA* **102**, 15545–15550 (2005). 16199517 10.1073/pnas.0506580102 PMC1239896

[87] Bligh, E. G. & Dyer, W. J. A rapid method of total lipid extraction and purification. *Can. J. Biochem. Physiol.* **37**, 911–917 (1959). 13671378 10.1139/o59-099

[88] Mason, R. J., Nellenbogen, J. & Clements, J. A. Isolation of disaturated phosphatidylcholine with osmium tetroxide. *J. Lipid Res.* **17**, 281–284 (1976). 932560

[89] Brown, K. et al. Epithelial Gpr116 regulates pulmonary alveolar homeostasis via Gq/11 signaling. *JCI Insight* **2**, e93700 (2017). 10.1172/jci.insight.93700 PMC5453702 28570277

[90] Charan, J. & Kantharia, N. D. How to calculate sample size in animal studies? *J. Pharm. Pharmacother.* **4**, 303–306 (2013). 10.4103/0976-500X.119726 PMC3826013 24250214

