# Supplementary material for: Receptor allostery promotes context-specific Sonic Hedgehog signaling during embryonic development
Source: Nat Commun. 2026 Jul 24;17:9040. doi: 10.1038/s41467-026-75918-5 (PMC13507325; doi:10.1038/s41467-026-75918-5)
Supplement: Supplementary file 1 [file 41467_2026_75918_MOESM1_ESM.pdf]

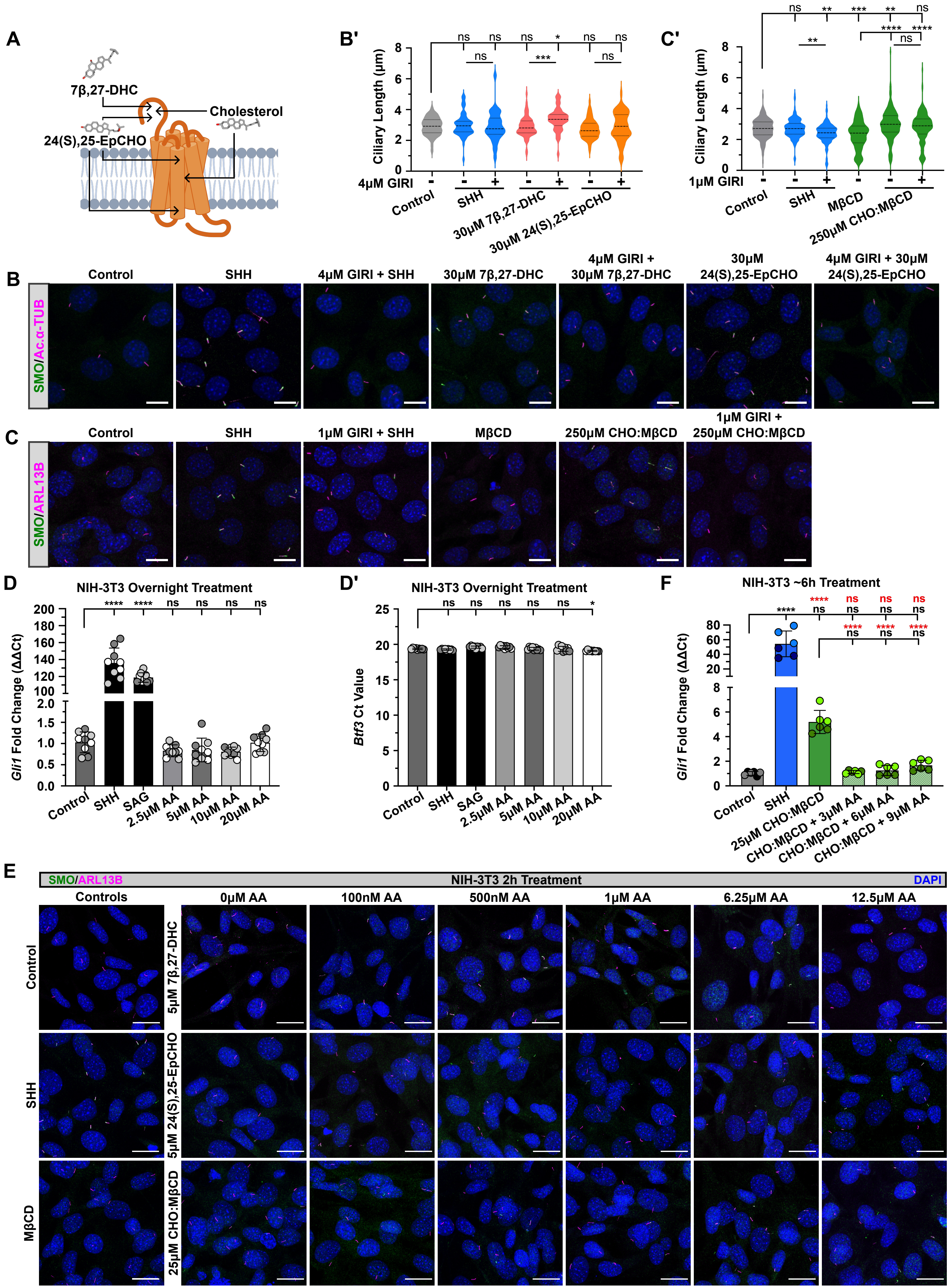

A
7β,27-DHC
Cholesterol
24(S),25-EpCHO
B'
Ciliary Length (μm)
4μM GIRI
Control
SHH
30μM 7β,27-DHC
30μM 24(S),25-EpCHO
C'
1μM GIRI
MβCD
250μM CHO:MβCD
B
SMO/Ac.α-TUB
Control
SHH
4μM GIRI + SHH
30μM 7β,27-DHC
4μM GIRI + 30μM 7β,27-DHC
30μM 24(S),25-EpCHO
4μM GIRI + 30μM 24(S),25-EpCHO
C
SMO/ARL13B
1μM GIRI + SHH
1μM GIRI + 250μM CHO:MβCD
D
NIH-3T3 Overnight Treatment
Gli1 Fold Change (ΔΔCt)
SAG
2.5μM AA
5μM AA
10μM AA
20μM AA
D'
Btf3 Ct Value
F
NIH-3T3 ~6h Treatment
25μM CHO:MβCD
CHO:MβCD + 3μM AA
CHO:MβCD + 6μM AA
CHO:MβCD + 9μM AA
E
SMO/ARL13B
NIH-3T3 2h Treatment
DAPI
Controls
0μM AA
100nM AA
500nM AA
1μM AA
6.25μM AA
12.5μM AA
5μM 7β,27-DHC
5μM 24(S),25-EpCHO
25μM CHO:MβCD

Supplementary Figure 1. Effects of cPLA2α inhibition and sterol treatment on SMO signaling. (A) Diagram of reported binding regions for oxysterols (7β,27-DHC and 24(S),25-EpCHO) and cholesterol (CHO) on SMO. Created in BioRender. Daly, C. (2026) https://BioRender.com/pvkwzoi. (B-C) Confocal images of NIH-3T3 cells show SMO (green) ciliary translocation following treatment with DMSO vehicle, the indicated agonists, and/or GIRI. Ac.α-Tubulin (B) and ARL13B (C) mark primary cilia in magenta. DAPI is blue. Scale bar = 10μm. Ciliary lengths were measured for 52-243 cilia per condition over at least 2 independent experiments (B' and C'). (D-D') qRT-PCR of NIH-3T3 cells treated overnight with control or SHH-N conditioned media (SHH), 100nM SAG, or increasing concentrations of arachidonic acid (AA). *Gli1* fold-change was calculated using the ΔΔCt method, normalized to the *Btf3* reference gene, shown in D'. (E) NIH-3T3 cells were treated for 2 hours with the indicated controls (left) or sterols with increasing concentrations of AA. Cilia are marked with ARL13B (magenta), and endogenous SMO (green). DAPI is blue. Scale bar = 20μm. (F) RT-qPCR of NIH-3T3 cells treated with control, SHH, or 25μM CHO:MβCD ± AA for ~6 hours. *Gli1* fold change was calculated as in (D). Statistical significance was calculated for the full data set (black indicators) and for the dataset with the SHH response omitted (red indicators). Data are plotted as mean ± S.D. Biological replicates are represented by different colored circles. Statistical significance was determined using two-way ANOVA (B', C', F) or one-way ANOVA (D-D') and indicated as follows: *<0.05, **<0.01, ***<0.001, ****<0.0001, and ns, p>0.05. Source data are provided as a Source Data file.

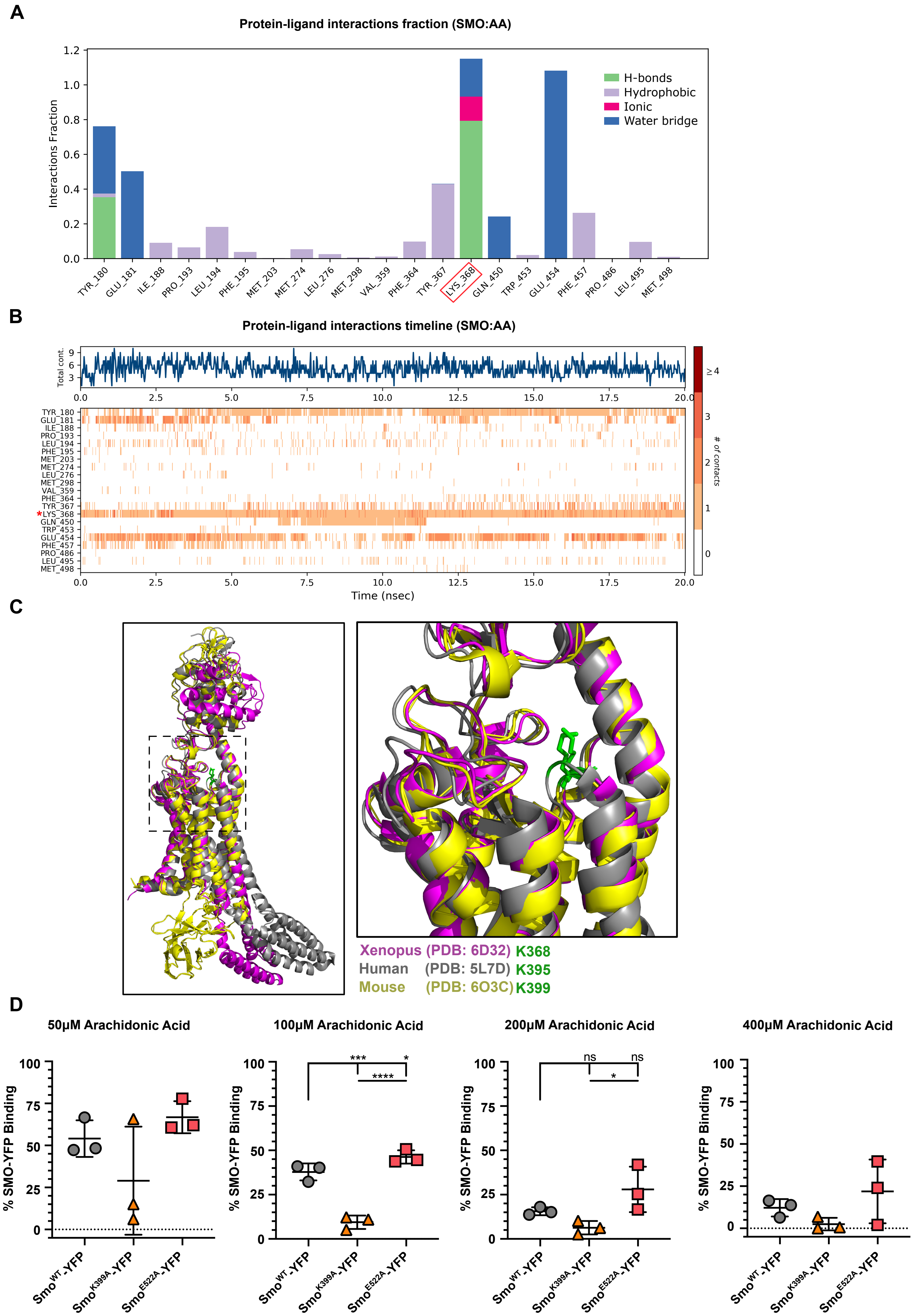
A
Protein-ligand interactions fraction (SMO:AA)
Interactions Fraction
1.2
1.0
0.8
0.6
0.4
0.2
0.0
H-bonds
Hydrophobic
Ionic
Water bridge
TYR_180
GLU_181
ILE_188
PRO_193
LEU_194
PHE_195
MET_203
MET_274
LEU_276
MET_298
VAL_359
PHE_364
TYR_367
LYS_368
GLN_450
TRP_453
GLU_454
PHE_457
PRO_486
LEU_495
MET_498
B
Protein-ligand interactions timeline (SMO:AA)
Total cont.
9
6
3
0.0
2.5
5.0
7.5
10.0
12.5
15.0
17.5
20.0
TYR_180
GLU_181
ILE_188
PRO_193
LEU_194
PHE_195
MET_203
MET_274
LEU_276
MET_298
VAL_359
PHE_364
TYR_367
*LYS_368
GLN_450
TRP_453
GLU_454
PHE_457
PRO_486
LEU_495
MET_498
≥4
3
2
1
0
# of contacts
Time (nsec)
C
Xenopus (PDB: 6D32) K368
Human (PDB: 5L7D) K395
Mouse (PDB: 6O3C) K399
D
50μM Arachidonic Acid
100μM Arachidonic Acid
200μM Arachidonic Acid
400μM Arachidonic Acid
% SMO-YFP Binding
100
75
50
25
0
***
*
****
ns
ns
*
Smo^WT-YFP
Smo^K399A-YFP
Smo^E522A-YFP

Supplementary Figure 2. AA is predicted to anchor in its binding pocket through association with K368 of Xenopus SMO and K399 of murine SMO. (A-B) Molecular dynamic simulation of AA binding to SMO predicts that K368 makes the most contacts with arachidonic acid (AA) (A), and that it forms the most consistent bond with the fatty acid over the course of the simulation (B). (C) Overlay of *Xenopus* (magenta), human (gray), and mouse (yellow) SMO proteins shows structural conservation surrounding the predicted AA binding pocket. The anchoring lysine is shown in green. (D) Murine SMO^{K399A} (orange) is significantly displaced from AA beads by lower concentrations of free AA than wild type SMO (WT, gray) or SMO^{E522A} (cyclopamine binding deficient, red). Data are plotted as mean ± S.D. For plots with a significant overall F-test, statistical significance was determined using Post-hoc pairwise comparisons and indicated as follows: *<0.05, ***<0.001, ****<0.0001, and ns, p>0.05. Source data are provided as a Source Data file.

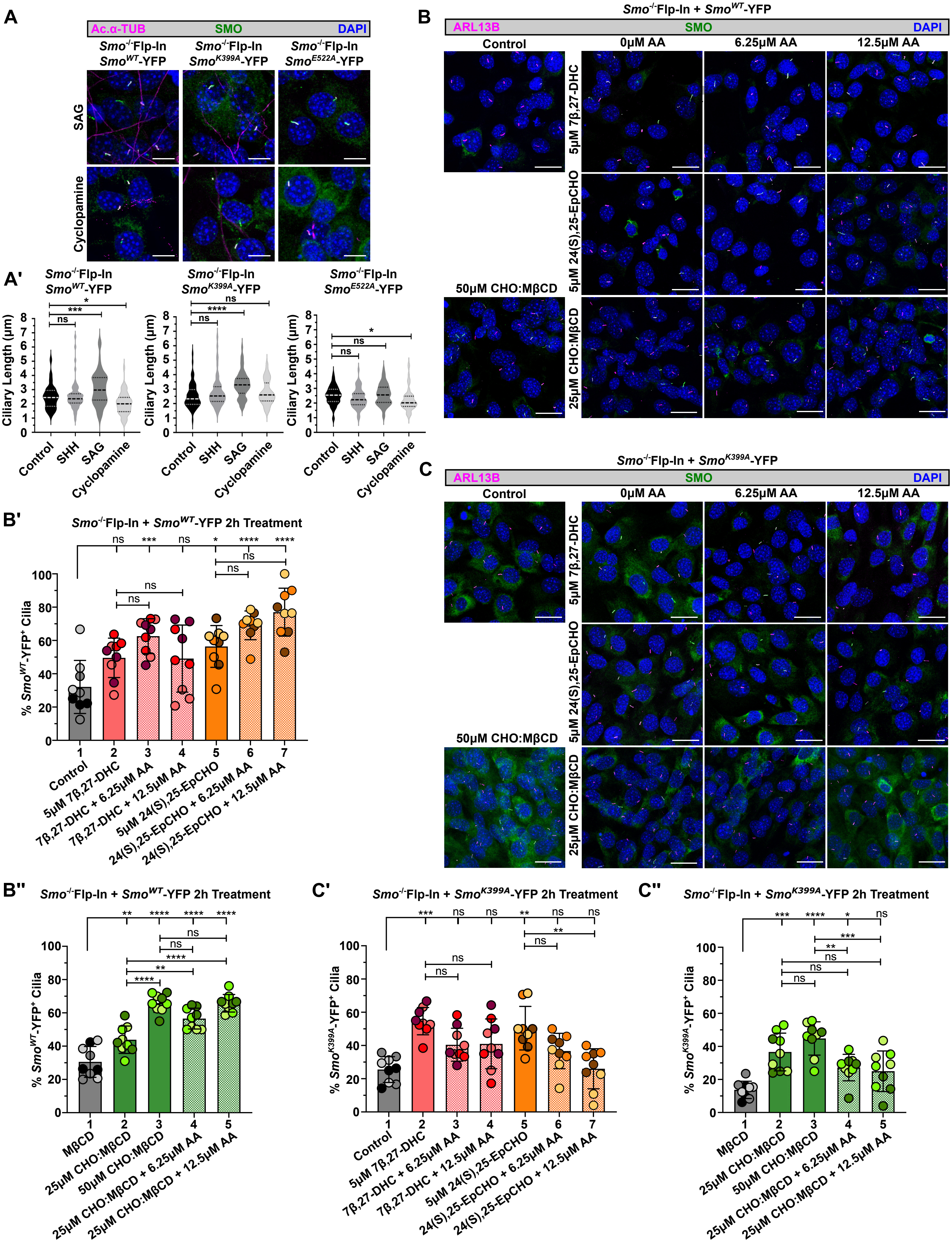

A
Ac.α-TUB
SMO
DAPI
Smo-/-Flp-In SmoWT-YFP
Smo-/-Flp-In SmoK399A-YFP
Smo-/-Flp-In SmoE522A-YFP
SAG
Cyclopamine
A'
Ciliary Length (μm)
Control
SHH
SAG
Cyclopamine
ns
***
*
****
B
Smo-/-Flp-In + SmoWT-YFP
ARL13B
Control
0μM AA
6.25μM AA
12.5μM AA
5μM 7β,27-DHC
5μM 24(S),25-EpCHO
50μM CHO:MβCD
25μM CHO:MβCD
C
Smo-/-Flp-In + SmoK399A-YFP
B'
Smo-/-Flp-In + SmoWT-YFP 2h Treatment
% SmoWT-YFP+ Cilia
5μM 7β,27-DHC
7β,27-DHC + 6.25μM AA
7β,27-DHC + 12.5μM AA
5μM 24(S),25-EpCHO
24(S),25-EpCHO + 6.25μM AA
24(S),25-EpCHO + 12.5μM AA
B''
MβCD
25μM CHO:MβCD
50μM CHO:MβCD
25μM CHO:MβCD + 6.25μM AA
25μM CHO:MβCD + 12.5μM AA
C'
Smo-/-Flp-In + SmoK399A-YFP 2h Treatment
% SmoK399A-YFP+ Cilia
C''

Supplementary Figure 3. SMO^{K399A}-YFP is insensitive to AA allostery. (A-A') The indicated *Smo*$^{-/-}$ Flp-In cells were treated with vehicle control, SHH conditioned media (SHH), 100nM SAG, or 10μM cyclopamine for ~18 hours and ciliary localization of SMO-YFP was evaluated. Representative images of SAG and cyclopamine-treated cells are shown. SMO is green, acetylated alpha-tubulin is magenta, and DAPI is blue. Scale bar = 10μm. (A') Ciliary length was measured for all conditions, totaling 68 cilia per condition for two biological replicates. (B,C) SMO ciliary translocation was evaluated by confocal microscopy in cells stimulated with the indicated oxysterol agonists in the absence or presence of arachidonic acid (AA). The ciliary axoneme is marked by ARL13B (magenta) and SMO-YFP is shown in green. DAPI (blue) marks nuclei. Scale bar = 20μm. (B'-B'',C'-C'') Percentages of SMO-YFP positive primary cilia were quantified in each condition. Each experiment was repeated three times resulting in a total of 137-277 cilia per condition. Bar graphs are plotted as mean ± S.D. Shaded data points indicate individual experiments. Significance was determined by one-way ANOVA (A') or two-way ANOVA (B'-C'') and is indicated as follows: *<0.05, **<0.01, ***<0.001, ****<0.0001, and ns, p>0.05. Source data are provided as a Source Data file.

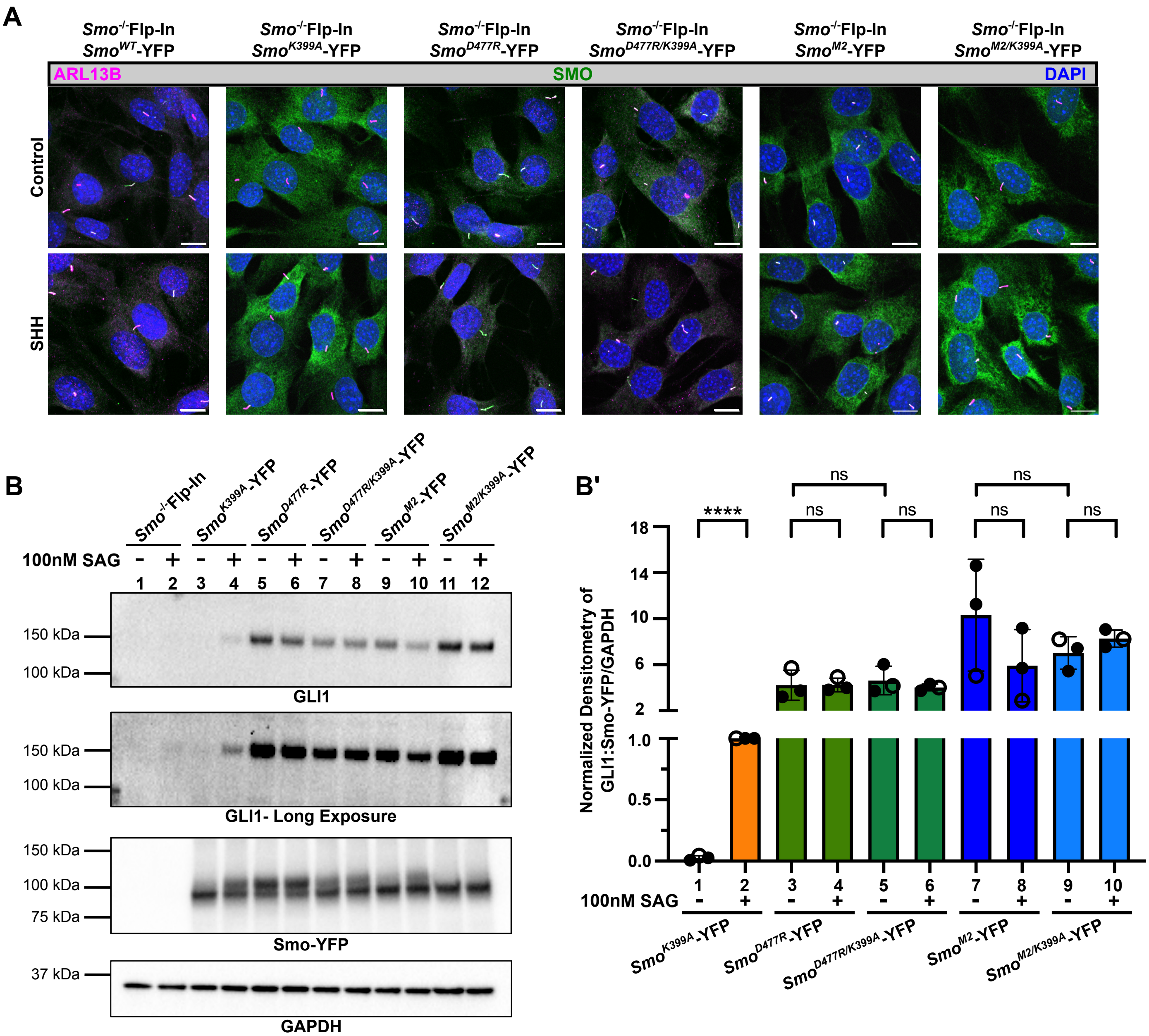
A
Smo-/-Flp-In SmoWT-YFP
Smo-/-Flp-In SmoK399A-YFP
Smo-/-Flp-In SmoD477R-YFP
Smo-/-Flp-In SmoD477R/K399A-YFP
Smo-/-Flp-In SmoM2-YFP
Smo-/-Flp-In SmoM2/K399A-YFP
ARL13B
SMO
DAPI
Control
SHH
B
Smo-/-Flp-In
SmoK399A-YFP
SmoD477R-YFP
SmoD477R/K399A-YFP
SmoM2-YFP
SmoM2/K399A-YFP
100nM SAG
1 2 3 4 5 6 7 8 9 10 11 12
150 kDa
100 kDa
GLI1
GLI1- Long Exposure
75 kDa
Smo-YFP
37 kDa
GAPDH
B'
****
ns
Normalized Densitometry of GLI1:Smo-YFP/GAPDH
18
14
10
6
2
1.0
0.5
0.0
1 2 3 4 5 6 7 8 9 10
100nM SAG
SmoK399A-YFP
SmoD477R-YFP
SmoD477R/K399A-YFP
SmoM2-YFP
SmoM2/K399A-YFP

Supplementary Figure 4. SMO^{K399A}-YFP is responsive to SAG. (A) The indicated *Smo*$^{-/-}$ Flp-In lines were treated with control or SHH-N conditioned media (SHH) for ~18 hours and SMO-YFP (green) ciliary localization was examined by confocal microscopy. ARL13B (magenta) marks primary cilia. DAPI (blue) marks nuclei. Scale bar = 10μm. (B-B') The indicated *Smo*$^{-/-}$ Flp-In lines were treated with vehicle or 100nM SAG for ~18 hours. GLI1 and SMO-YFP protein levels were evaluated by western blot and densitometry across 3 independent experiments. GLI1 protein levels were calculated relative to SMO-YFP and normalized to GAPDH loading control. Data are represented as mean ± S.D. Significance was calculated by one-way ANOVA and is indicated as follows: ****<0.0001, and ns, p>0.05. Source data are provided as a Source Data file.

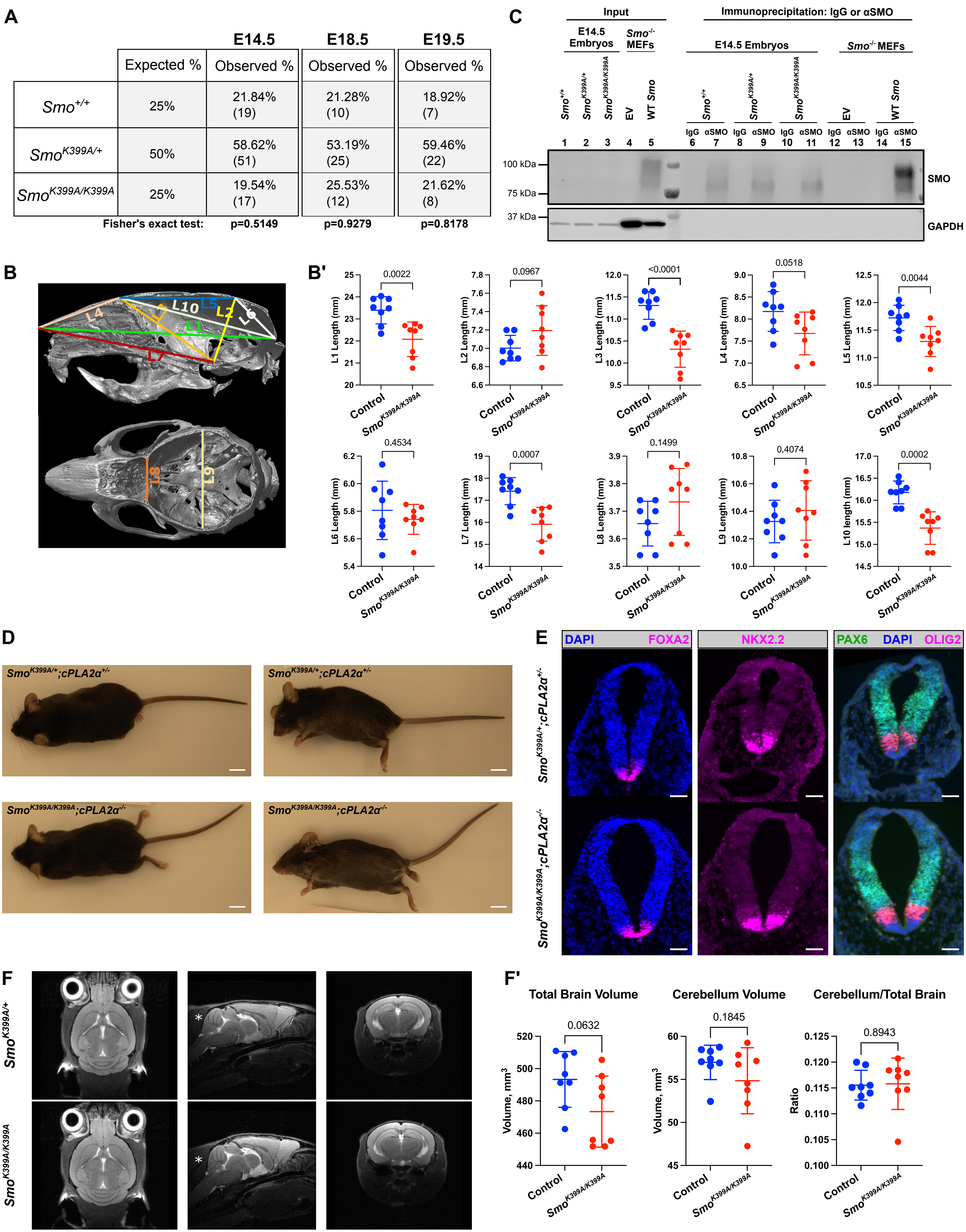

	Expected %	E14.5 Observed %	E18.5 Observed %	E19.5 Observed %
Smo+/+	25%	21.84% (19)	21.28% (10)	18.92% (7)
*Smo*K399A/+	50%	58.62% (51)	53.19% (25)	59.46% (22)
*Smo*K399A/K399A	25%	19.54% (17)	25.53% (12)	21.62% (8)
Fisher's exact test:		p=0.5149	p=0.9279	p=0.8178

Supplementary Figure 5. Analysis of *Smo*$^{K399A/K399A}$ mice. (A) *Smo*$^{K399A/K399A}$ knock-in mice show normal Mendelian ratios at E14.5, E18.5, and E19.5. Fisher's exact test values were conducted for each developmental stage. (B) Diagram of micro-computed tomography (microCT) linear-based measurement landmarks that were used for analysis of skulls along the sagittal and horizontal planes for 8 control (3 *Smo*$^{+/+}$ and 5 *Smo*$^{K399A/+}$) and 8 *Smo* $^{K399A/K399A}$ mice. (B') Linear measurements of indicated landmarks (L1-10) were calculated. (C) A western blot shows endogenous SMO protein in embryo lysates (lanes 1-3) and immunoprecipitates from embryo lysates of the indicated genotypes (lanes 6-11). SMO protein levels are too low to detect in lysates. *Smo*$^{-/-}$ MEFs ± SMO re-expression serve as controls (lanes 4-5, 12-15). The experiment was repeated three times, and a representative blot is shown. (D) One of two *Smo*$^{K399A/K399A}$;*cPLA2α*$^{-/-}$ mice that survived to weaning is shown compared to the heterozygous littermate control. (E) Neural tubes of E9.5/25-29 somite stage *Smo*$^{K399A/+}$ and *Smo*$^{K399A/K399A}$ embryos show normal *FoxA2, Nkx2.2, Olig2*, and *Pax6* progenitor domains. At least 3 sections each from 3 embryos were analyzed. A representative section is shown. Scale bar = 50μm. (F-F') Magnetic resonance imaging (MRI) scans of 8 control (3 *Smo*$^{+/+}$ and 5 *Smo*$^{K399A/+}$) and 8 *Smo* $^{K399A/K399A}$ brains from adult mice show similar brain morphology (F), and cerebellum volume relative to total brain volume was comparable (F'). For all graphs, male and female data were combined, and results are shown as mean ± S.D. Statistical significance was calculated using two-tailed Student's t-tests. Source data are provided as a Source Data file.

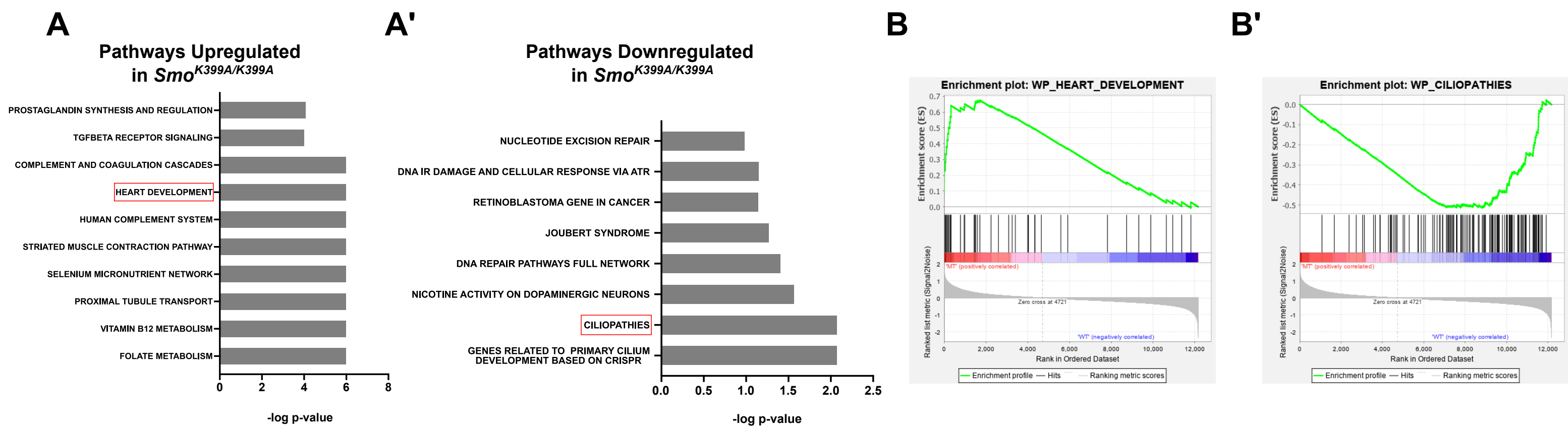

C

	GO TERMS UPREGULATED IN $Smo^{K399A/K399A}$	FDR q- value
GO Cellular Component	BRUSH BORDER	< 1.0 e-6
	ACTIN FILAMENT BUNDLE	< 1.0 e-6
	CONTRACTILE FIBER	7.4 e-5
GO Biological Process	PLASMA LIPOPROTEIN PARTICLE CLEARANCE	< 1.0 e-6
	POSITIVE REGULATION OF EPITHELIAL CELL MIGRATION	< 1.0 e-6
	GROWTH FACTOR BINDING	< 1.0 e-6
GO Molecular Function	EXTRACELLULAR MATRIX STRUCTURAL CONSTITUENT	0.0006
	GROWTH FACTOR BINDING	0.001
	TRANSFORMING GROWTH FACTOR BETA BINDING	0.002

C'

	GO TERMS DOWNREGULATED IN $Smo^{K399A/K399A}$	FDR q- value
GO Cellular Component	CENTRIOLE	0.0006
	REPLICATION FORK	0.03
	CILIARY BASAL BODY	0.05
GO Biological Process	CILIARY BASAL BODY PLASMA MEMBRANE DOCKING	0.007
	AXONEME ASSEMBLY	0.01
	CILIUM ORGANIZATION	0.02
GO Molecular Function	CATALYTIC ACTIVITY ACTING ON DNA	0.05
	DAMAGED DNA BINDING	0.06
	DEOXYRIBONUCLEASE ACTIVITY	0.07

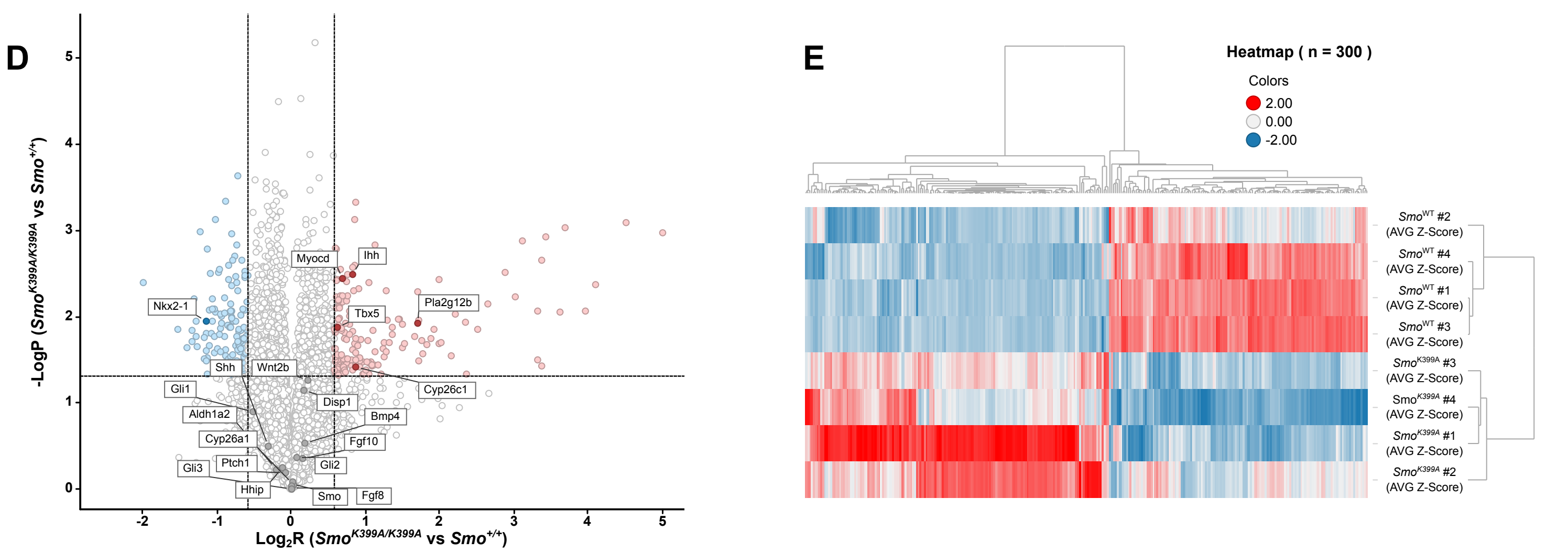

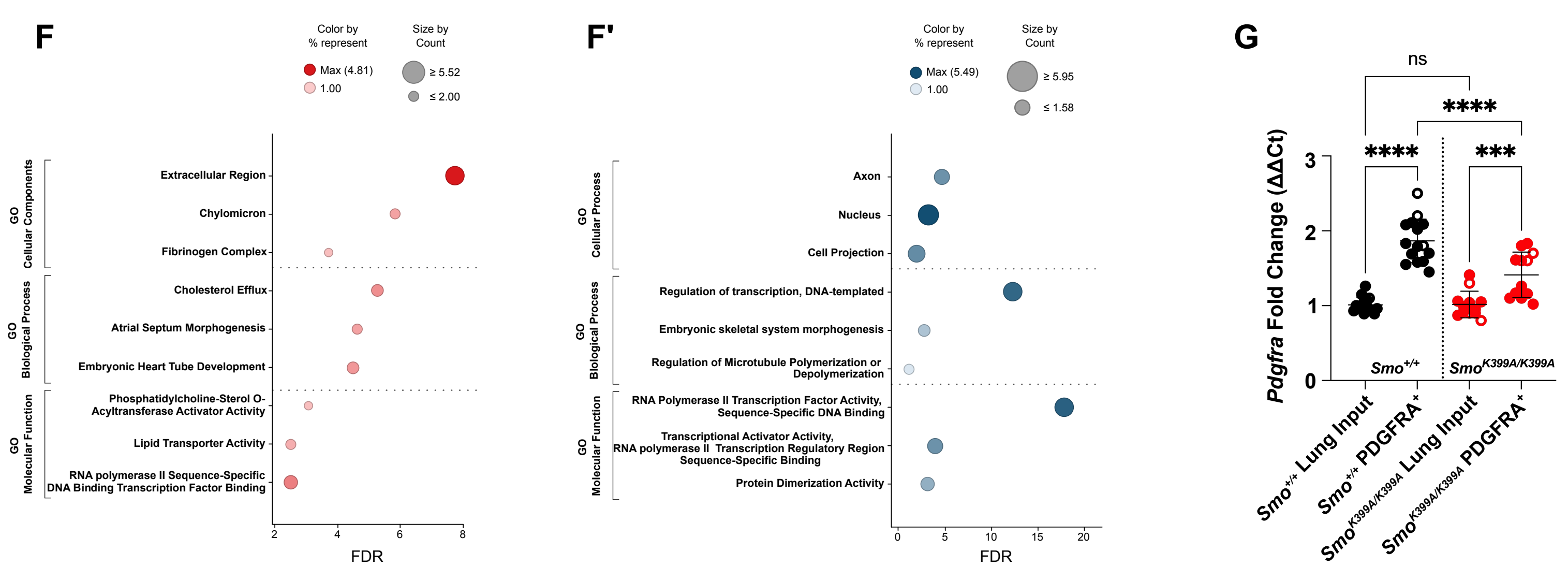

Supplementary Figure 6. Gene expression analysis of E9.5/25-29 somite stage *Smo*^{WT} and *Smo*$^{K399A/K399A}$ mutant embryos. Wiki pathways upregulated (A) and downregulated (A') Gene Set Enrichment Analyses (GSEA) for *Smo*$^{K399A/K399A}$ embryos compared to *Smo*$^{+/+}$ are shown. *n* = 4 embryos per genotype. Representation of the positively correlated enrichment of heart development (B) and negatively regulated (B') genes related to ciliopathies in *Smo*$^{K399A/K399A}$ embryos are shown compared to *Smo*$^{+/+}$ control. The top three Gene Ontology (GO) terms of cellular component, biological process, and molecular function upregulated (C) and downregulated (C') in *Smo*$^{K399A/K399A}$ embryos compared to *Smo*$^{+/+}$ are shown. (D) A volcano plot with the log10 of the p-value versus the log ratio fold-change in differentially expressed genes (DEGs) in *Smo*$^{K399A/K399A}$ embryos compared to *Smo*$^{+/+}$ are shown. Genes with p-value <0.01 and log2ratio >0.5 (upper right quadrant, red) and <0.5 (upper left quadrant, blue) are shown. (E) A heatmap of the top statistically significant differentially regulated genes shows ranking by expression value in log scale. (F-F') Top three GO terms for statistically significant DEGs in *Smo*$^{K399A/K399A}$ embryos as compared to *Smo*$^{+/+}$ with p<0.05 (302 genes) using EnrichR. The x-axis represents the false discovery rate (FDR), which refers to the proportion of false discoveries among a set of hypothesis tests. The circle size indicates the number of DEGs that are associated with each significant pathway. The circle color indicates the significant level with the adjusted p-value <0.05. (G) qRT-PCR analyses showing *Pdgfra* expression in E18.5 (closed circles) and p0 (open circles) PDGFRA^{+} cells isolated from lungs of control and *Smo*$^{K399A/K399A}$ mutant mice. Elevated *Pdgfrα* expression in the anti-PDGFRA-biotin-enriched lung cells following streptavidin pull down confirms successful PDGFRA^{+} cell isolation. Fold-change in expression was determined using the ΔΔCt method against *Btf3* and *Ppia* reference genes. Average fold change was calculated across five *Smo*$^{+/+}$ and four *Smo*$^{K399A/K399A}$ individuals analyzed in triplicate. Data are plotted as mean ± S.D. Significance was determined by one-way ANOVA. Significance is denoted as follows: ***<0.001, ****<0.0001 and ns, p>0.05. Source data are provided as a Source Data file.

	Expected %	Observed %
***Smo*$^{+/+}$*;cPLA2α*$^{+/+}$**	6.25%	15.67% (42)
***Smo*$^{+/+}$*;cPLA2α*$^{+/-}$**	12.5%	19.03% (51)
***Smo*$^{+/+}$*;cPLA2α*$^{-/-}$**	6.25%	6.34% (17)
***Smo*$^{K399A/+}$*;cPLA2α*$^{+/+}$**	12.5%	18.28% (49)
***Smo*$^{K399A/+}$*;cPLA2α*$^{+/-}$**	25.0%	25.37% (68)
***Smo*$^{K399A/+}$*;cPLA2α*$^{-/-}$**	12.5%	10.45% (28)
***Smo*$^{K399A/K399A}$*;cPLA2α*$^{+/+}$**	6.25%	2.61% (7)
***Smo*$^{K399A/K399A}$*;cPLA2α*$^{+/-}$**	12.5%	1.49% (4)
***Smo*$^{K399A/K399A}$*;cPLA2α*$^{-/-}$**	6.25%	0.75% (2)

Fisher's exact test p<0.0001

Supplementary Table 1. *Smo*$^{K399A/K399A}$*, Smo*$^{K399A/K399A}$*;cPla2α*$^{+/-}$*, and Smo*$^{K399A/K399A}$;*cPLA2α*$^{-/-}$ mutant mice show enhanced perinatal lethality as indicated by skewed Mendelian ratios between birth and weaning. A two-tailed Fisher's exact test was performed to analyze the significance between expected and observed outcomes; $p = 1.83 \times 10^{-9}$. *n*=268 mice. Source data are provided as a Source Data file.

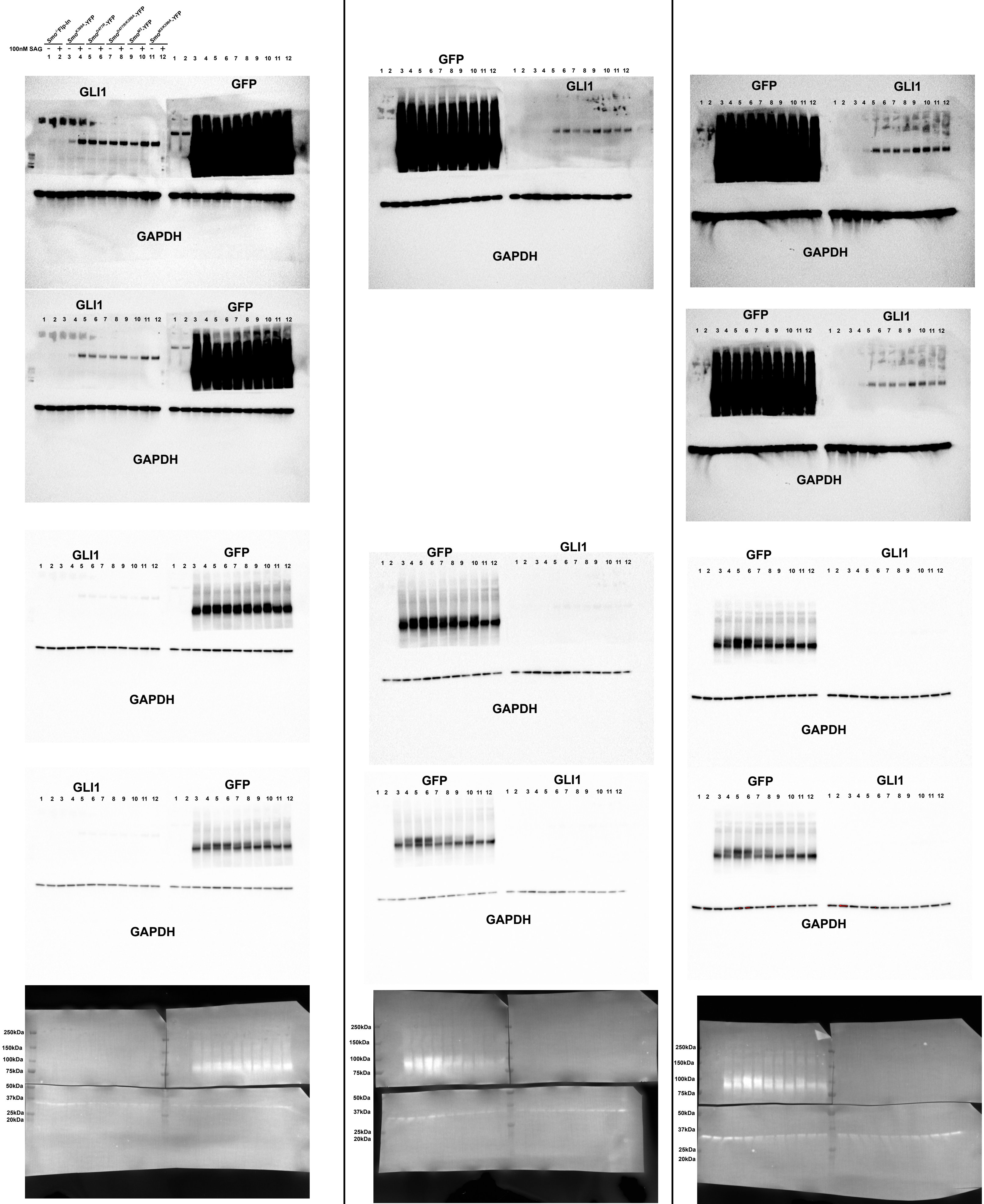

Replicate 1: for Figure | **Replicate 2** | **Replicate 3**

Source blots for Supplementary Figure 4B.

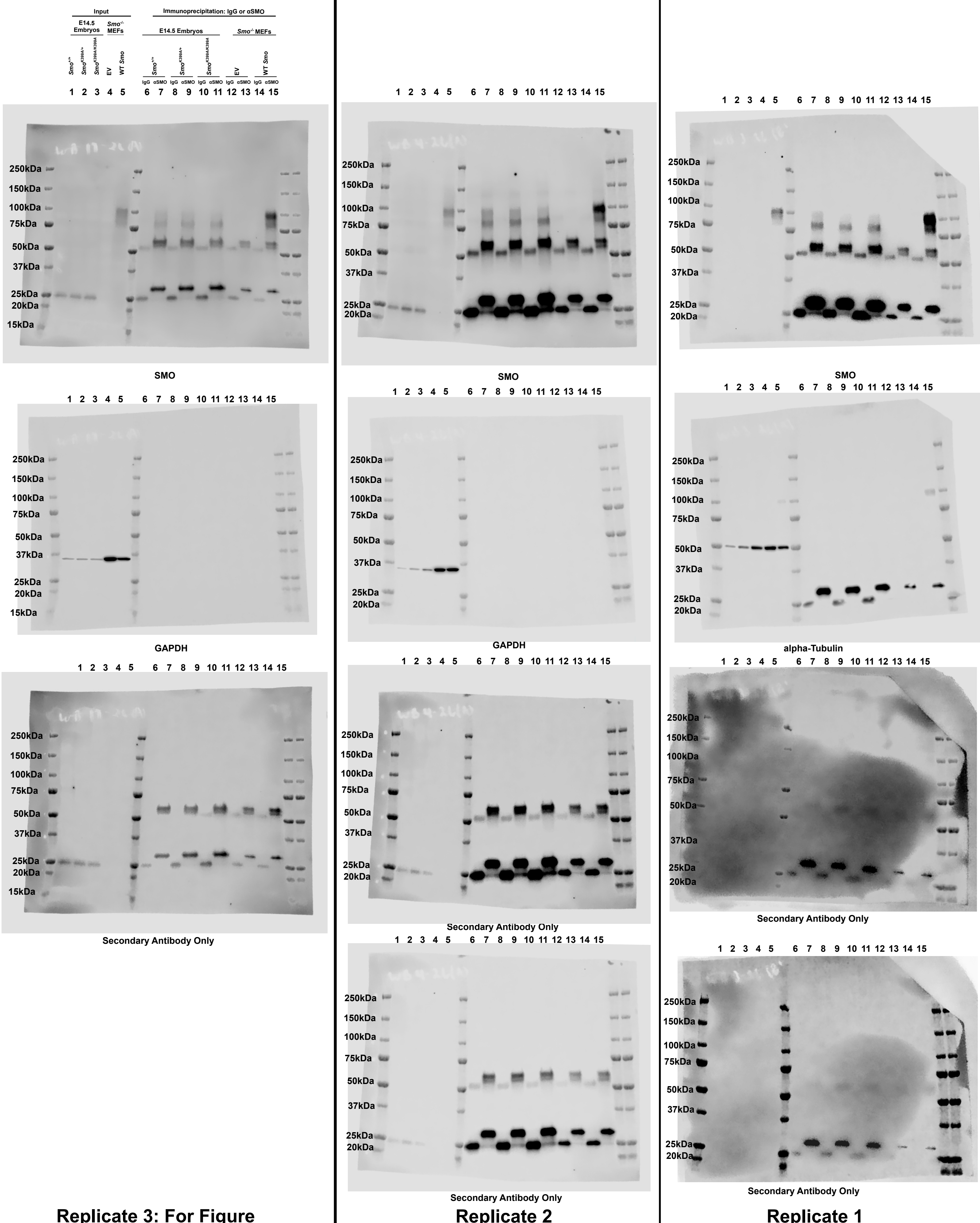

Input
Immunoprecipitation: IgG or αSMO
E14.5 Embryos
Smo-/- MEFs
EV
WT Smo
IgG
αSMO
1 2 3 4 5 6 7 8 9 10 11 12 13 14 15
250kDa
150kDa
100kDa
75kDa
50kDa
37kDa
25kDa
20kDa
15kDa
SMO
GAPDH
alpha-Tubulin
Secondary Antibody Only
Replicate 3: For Figure
Replicate 2
Replicate 1

Source blots for Supplementary Figure 5C.
