# Supplementary material for: Receptor allostery promotes context-specific Sonic Hedgehog signaling during embryonic development
Source: Nat Commun. 2026 Jul 24;17:9040. doi: 10.1038/s41467-026-75918-5 (PMC13507325; doi:10.1038/s41467-026-75918-5)
Supplement: Supplementary file 2 [file 41467_2026_75918_MOESM2_ESM.pdf]

nature portfolio

Peer Review File

Receptor Allostery Promotes Context-Specific Sonic Hedgehog Signaling During Embryonic Development

Corresponding Author: Dr Stacey Ogden

This file contains all reviewer reports in order by version, followed by all author rebuttals in order by version.

Version 0:

Reviewer comments:

Reviewer #1

(Remarks to the Author)
Review of "Receptor allostery promotes context-dependent Sonic Hedgehog signaling during embryonic development" (Ansari et al. 2025)

Summary of findings and noteworthy results: The Ogden Lab (Arensdorf et al., 2017) previously demonstrated that cytosolic phospholipase A2α (cPLA2α) is activated through Gβγ signaling, leading to the release of arachidonic acid (AA). In a positive feedforward loop, AA binds to Smoothened (SMO) and synergizes with the cysteine-rich domain (CRD)-binding agonist 20(S)-hydroxycholesterol to enhance SMO activation. While this study provided valuable mechanistic insights, it was conducted exclusively in cultured cells, leaving open the question of whether this regulatory mechanism plays a biologically significant role in vivo. Building upon these findings, Ansari et al. (2025) demonstrate that cPLA2α-produced AA functions as a key allosteric enhancer of SMO activity, capable of amplifying pathway activation in response to multiple oxysterols (beyond 20(S)-hydroxycholesterol) and cholesterol. Using in silico docking assays, the authors predicted that AA binds to the top of the seven-transmembrane (7TM) pocket of SMO, thereby enhancing sterol-driven activation. They validate this prediction with ligand-induced thermal stability assays, showing that mutation of this site (SMOK399A) disrupts AA binding. To assess the in vivo significance of this interaction, the authors employ CRISPR/Cas9 to generate a K399A knock-in mouse. Interestingly, the phenotypes of the SmoK399A/K399A mice are tissue specific. SmoK399A/K399A mice exhibit congenital heart defects, pulmonary maldevelopment, and neonatal respiratory failure, while neural development remains unaffected. Developmental and transcriptomic analyses further reveal SHH-dependent defects in heart septation, lung fibroblast differentiation, and surfactant production, highlighting a previously unappreciated role for AA-SMO interactions in cardiopulmonary development. Together, these findings provide compelling in vivo evidence that cPLA2α-mediated AA production is a critical modulator of Hedgehog signaling, selectively influencing tissue-specific developmental processes.

Overall, I think this is a significant advance in the field. By utilizing a combination of biochemical, structural, and genetic approaches, this study extends previous cell-based findings to demonstrate a physiologically relevant role for AA-SMO interactions in cardiopulmonary development. The findings open up new avenues for investigating lipid-mediated modulation of Hh signaling.

I have a few concerns, which I would appreciate the authors addressing.
-The reduced SHH response in the SmoK399A addback cells is an important finding. However, I was surprised that the authors did not conduct a similar analysis with the addition of AA, oxysterols, and cholesterol (as done in Figure 2). Testing these conditions would more directly demonstrate that the K399A mutation indeed disrupts AA-mediated activation of SMO. Can the authors clarify why this was not performed or consider adding this analysis?
-While the SmoD477R mutant serves as a useful control, the reduced surface localization of SmoK399A (Supplementary Figure 2B) raises concerns about whether this mutation affects protein folding rather than just AA binding. If misfolding is an issue, it could explain both the reduced signaling in vitro and the developmental defects observed in vivo. To rule this out, could the authors provide a Western blot from SmoK399A/K399A mice to confirm that total SMO protein levels are comparable to WT?
- One aspect that I found confusing is the apparent lack of developmental phenotypes in cPLA2α-/- mice. Bonventre et al. (1997) previously reported that cPLA2α-/- mice develop normally, and this finding is supported by the authors' own data (Supplementary Table 1), where the knockout mice appear at expected Mendelian ratios. Given that cPLA2α is proposed to be a key producer of AA, I would have expected the cPLA2α-/- phenotype to be at least as severe, if not more so, than that of

SmoK399A/K399A mice. This raises the possibility that AA can still be produced through alternative pathways? Can the authors comment on whether other enzymes compensate for AA production in cPLA2α-/- mice? Additionally, is it possible that the phenotypes observed in SmoK399A/K399A mice are due to reduced SMO levels rather than its inability to bind AA? Is it possible to measure AA levels in cPLA2α-/- cells or tissues? This would help clarify whether AA is still being produced in these mutants.

Reviewer #2

(Remarks to the Author)
This is an exciting and well-written manuscript from Ogden and colleagues. The authors have used cellular assays, computational docking and mouse models to explore the role of arachidonic acid in regulating SMO in vitro and in vivo. Important findings of this research are the unusual tissue-specificity of the mouse mutant phenotype, and the clear dose-responsive synergy between Cholesterol:MβCD and arachidonic acid in GLI reporter assays. Overall the reviewer supports publication; however, there are some major issues that need to be addressed.

The major concerns are firstly to determine if the differences in arachidonic acid binding pose for Xenopus SMO versus the murine SMO structures have any impact on the importance of the murine K399 residue. Further computational work (e.g. computational molecular dynamics simulations) could help address this concern. Secondly, there is a need to further clarify the effect of the K399A mutant on SMO glycosylation state and stability, and the degree to which this mutant disrupts arachidonic acid binding (total versus partial). Appropriate experiments with purified proteins would address these concerns.

Specific comments:

- Line 61: "Removal of PTCH1 from ciliary and plasma membranes allows for localized accumulation of sterols that bind SMO to stimulate its PC accumulation and signaling through two distinct effector routes" - Has this been shown previously? The reviewer is not aware of any publication measuring sterol concentrations in the PC.

- Line 64: The distinction between canonical and non-canonical HH signaling, and which of the pathways is relevant for this publication is not clear – please specify and clarify in the text.

- Line 116: SSTR3 is a ciliary marker and is always expressed in the primary cilium, so there will be SSTR3 in the cilium before treatment. For this reason, it is difficult to use SSTR3 as a control for SMO specific dynamic localization to the PC. Another membrane protein that is dynamically regulated within the primary cilium could control for SMO specificity. Maybe clarify this point in the text.

- Figure 1: In microscopy images, it is probably clearer to keep each protein in the same color throughout the figure (and if possible throughout all the figures). It would also be clearer if the DAPI channel is labelled separately, as the blue is tricky to distinguish from the cyan.

- Figure 1: Check if a one-way or a two-way ANOVA is appropriate, given there are two independent variables (SHH treatment, and GIRI treatment).

- Line 168: To show that GIRI is specifically acting through cPLA2α, the authors might consider a rescue experiment where GIRI inhibition of cPLA2α is combined with exogenous AA treatment.

- Figure 3: consistent colors, background, and labeling of key amino acids on all panels would help interpretation of this figure. For the red color scheme on the conservation analysis, it is unclear which of the lighter shades represents highest conservation.

- Figure 3: in the text there should be some comment and analysis of why the binding pose of arachidonic acid is different in the murine structure compared to the Xenopus structure, and what implications this has for the expected role of mouse K399 in arachidonic acid binding. This residue should be labelled in the mouse structures.

- Line 190: To test the effect of AA on TMD cholesterol binding and provide further evidence for the identified salt bridge between K368 and the AA hydroxyl group, it might be useful to carry out some molecular dynamics simulations. These would also confirm the overall binding pose of AA in general.

- Line 203: The stability assay uses crude membrane extracts. This will not provide any information of potentially destabilized and/or unfolded protein sample. To properly assess the stability of the mutants, the reviewer suggests carrying out the thermostability assay with purified proteins.

- Line 232: "We confirmed that SMOK399A and SMOE522A mutant proteins reached the cell surface like WT SMO-YFP" There is concern about the stability of the SMO-K399A mutant. Although stated, the Western blot analysis clearly indicates different behaviour in glycosylation state compared to wildtype (no clear band for the glycosylated post-ER form). This could

be indicative of a protein that is less stable. To prove stability and integrity of the SMO-K399A mutant, the authors could carry out thermostability assays with purified proteins (see above).

- Line 269: ‘Double mutant’ might be a clearer term than ‘compound mutant’, to avoid confusion with compounds/ligands.

- Line 274: Analysis of the ciliary localization of these double mutants (as was completed for the single mutants) might provide further insight into these results.

-Line 304: “may represent phenotypic “escapers” with compensatory signaling activity”. Some explanation is needed to clarify this hypothesis (rather than SmoK399A/K399A simply being epistatic over Pla2α-/- with respect to the size reduction phenotype, because SMO is acting downstream of PLA2α in the signalling pathway).

-Line 299: Would we expect the double mutant to phenocopy rather than exacerbate the single mutant if there is loss of function in the same pathway? The authors might want to mention here that SMO and PLA2a loss of function could have distinct effects that only synergize at the level of the whole mouse's development (as they comment on in the discussion, line 446).

- Line 465: Given several experiments in this publication suggest that arachidonic acid binding is required for SHH-mediated SMO activity, perhaps some further comment should be made about whether arachidonic acid is considered a necessary ligand or a dispensable allosteric modulator.

- General comment: It would be informative if experiments characterizing primary cilia would include an analysis of the lengths of the imaged primary cilia, to check whether mutations and/or drug treatments have any major effects on cilium integrity. This is especially the case given the in vivo RNA-seq data for the K399A/K399A embryos indicates disruption of genes regulating PC integrity (line 345).

Reviewer #3

(Remarks to the Author)

This study investigates how arachidonic acid (AA) binds to a putative allosteric site in the 7TM domain of Smoothened (SMO) and its implications for Hedgehog signaling. The authors build on previous work from the Ogden lab (e.g., Arensdorf et al, Cell Reports 2017) by mapping this AA-binding site and testing its functional importance in cell-based and in vivo models. The identification of a specific SMO mutation (K399A) that disrupts AA binding is intriguing, the in vivo analysis of this knock-in allele provides intriguing developmental phenotypes, and the integration of structural, functional, and in vivo assays is impressive. Unfortunately, however, significant technical and interpretational issues, particularly regarding the thermal shift assays, ciliary localization assays, and functional assays, weaken the strength of the conclusions. In addition, while the mouse mutant supports the functional importance of this allosteric site for some aspects of Hedgehog signaling, the biochemical and cultured cell data do not definitively map it as an AA-specific binding site. The same site is also used by 24,25-epoxycholesterol and potentially other endogenous SMO ligands, which the authors have not ruled out.

Major Concerns

1. Lack of strong evidence for AA binding specificity
-The thermal shift assay (Fig 4) used to infer AA binding to SMO lacks proper controls and standardization, making it difficult to draw conclusions from the data.
-The data are fit with a curve using GraphPad prism, but no details are provided on the fitting method –many individual data points deviate significantly from the fitted curve, and the shapes of the curves are vastly different for the wild-type and mutant SMO proteins. This makes it very difficult to evaluate the data.
-The actual thermal shift in SMO upon AA binding appears modest, raising concerns that the effect of AA may be very modest and that the assay may not be detecting a meaningful, specific binding interaction.
-The K399A mutant shows a significantly altered thermal profile compared to wild-type SMO, suggesting potential structural instability rather than a specific loss of AA binding.
-The cyclopamine-resistant E518A mutant still shows a substantial shift upon cyclopamine treatment (comparable in magnitude to the effect of AA on wild-type SMO), further calling into question whether the assay is measuring specific ligand interactions.
-No negative control is shown for AA (i.e., a non-AA-binding GPCR, such as SSTR3), which is necessary to show that the effect of AA on SMO in this assay reflects a specific binding event (as opposed to a nonspecific effect on SMO or on the assay in general).
-No raw data are presented for the assay – only analyzed, quantified data. Raw data should be shown for the blots used in this analysis.

If the goal is to demonstrate AA binding disruption, it would be much more effective and convincing to use direct ligand binding assay, such as the bead-based AA binding assay they developed in their 2017 study. Such an assay is more direct and interpretable than the thermal shift assay, which is an indirect proxy for binding.

2. Issues with GLI reporter assays and cellular experiments
-The GLI reporter assays (Fig. 2F, 2G) show that AA synergizes with SMO agonists, but the authors do not test whether AA

enhances SMO ciliary localization in response to these agonists —a critical mechanistic prediction of their model. Instead, they only test whether blocking AA metabolism (via GIRI) reduces SMO localization.
-The Firefly/Renilla luciferase ratio is used to measure GLI reporter activity, but given that AA is highly hydrophobic and is being used at relatively high concentrations (15 micromolar) over a 36-hour incubation, it could cause unintended effects on cell viability or on Renilla luciferase activity, artifactually increasing the Firefly/Renilla ratio. The authors should show raw Renilla readings from their assays, perhaps in a supplementary figure, to exclude this possibility.
-The authors claim that SMO K399A undergoes normal glycosylation and surface expression, but in Supp Fig 2A-B, the K399A mutant exhibits reduced glycosylation and markedly reduced surface expression. This discrepancy should be addressed, as it complicates interpretation of the in vivo and signaling defects.
-Figs 5E and E' – the GLI1 western blot shows very low signal to noise, with only 2 fold induction over background (and large error bars) for wild-type SMO. This makes it very difficult to assess the functional effects of the mutants. GLI transcriptional reporter assay, as in Fig 2, or a Gli1 qPCR measurement, would be a much more accurate and quantitative way to assess signaling activity of the SMO mutants.

3. Issues with interpretation of in vivo findings
-The K399A knock-in mouse reveals that the shallow 7TM site is important for SMO function, but the authors overstate the role of AA as the primary endogenous ligand for this site. This site is also used by other sterol-like molecules, including 24,25-epoxycholesterol, which has not been ruled out.
-The cPLA2 knockout is claimed to synergize with SMO K399A, yet the authors never analyze the double mutant in the heart or lung, which are the tissues most affected by SMO K399A. Without this, the claim of synergy between cPLA2 and SMO K399A is unsupported and could simply be independent, unrelated effects.

Version 1:

Reviewer comments:

Reviewer #1

(Remarks to the Author)
Thank you to the authors for addressing my comments. I continue to believe this is a great paper and will be a significant contribution to the field. I fully support its publication.

Reviewer #2

(Remarks to the Author)
The authors have now addressed all of my comments and concerns. With the inclusion of the new data and the revisions made to the manuscript, the work has improved substantially and the conclusions are now more convincing. I therefore recommend the manuscript for publication.

Reviewer #3

(Remarks to the Author)
The authors have addressed my concerns, and I now support publication.

Open Access This Peer Review File is licensed under a Creative Commons Attribution 4.0 International License, which permits use, sharing, adaptation, distribution and reproduction in any medium or format, as long as you give appropriate credit to the original author(s) and the source, provide a link to the Creative Commons license, and indicate if changes were made.
In cases where reviewers are anonymous, credit should be given to 'Anonymous Referee' and the source.
The images or other third party material in this Peer Review File are included in the article's Creative Commons license, unless indicated otherwise in a credit line to the material. If material is not included in the article's Creative Commons license and your intended use is not permitted by statutory regulation or exceeds the permitted use, you will need to obtain permission directly from the copyright holder.
To view a copy of this license, visit https://creativecommons.org/licenses/by/4.0/

We thank the reviewers for carefully evaluating our manuscript and providing constructive suggestions to improve it. We have repeated multiple experiments using alternative/improved methods and added several new experiments to address the critiques. We hope the reviewers will agree that the additions and text revisions to the manuscript have significantly strengthened the overall study and that the work is now suitable for publication. Below, we provide our point-by-point responses to the reviewers' comments.

REVIEWER COMMENTS

Reviewer #1 (Remarks to the Author):

Review of "Receptor allostery promotes context-dependent Sonic Hedgehog signaling during embryonic development" (Ansari et al. 2025)

Summary of findings and noteworthy results: The Ogden Lab (Arensdorf et al., 2017) previously demonstrated that cytosolic phospholipase A2α (cPLA2α) is activated through Gβγ signaling, leading to the release of arachidonic acid (AA). In a positive feedforward loop, AA binds to Smoothened (SMO) and synergizes with the cysteine-rich domain (CRD)-binding agonist 20(S)-hydroxycholesterol to enhance SMO activation. While this study provided valuable mechanistic insights, it was conducted exclusively in cultured cells, leaving open the question of whether this regulatory mechanism plays a biologically significant role in vivo. Building upon these findings, Ansari et al. (2025) demonstrate that cPLA2α-produced AA functions as a key allosteric enhancer of SMO activity, capable of amplifying pathway activation in response to multiple oxysterols (beyond 20(S)-hydroxycholesterol) and cholesterol. Using in silico docking assays, the authors predicted that AA binds to the top of the seven-transmembrane (7TM) pocket of SMO, thereby enhancing sterol-driven activation. They validate this prediction with ligand-induced thermal stability assays, showing that mutation of this site (SMOK399A) disrupts AA binding. To assess the in vivo significance of this interaction, the authors employ CRISPR/Cas9 to generate a K399A knock-in mouse. Interestingly, the phenotypes of the SmoK399A/K399A mice are tissue specific. SmoK399A/K399A mice exhibit congenital heart defects, pulmonary maldevelopment, and neonatal respiratory failure, while neural development remains unaffected. Developmental and transcriptomic analyses further reveal SHH-dependent defects in heart septation, lung fibroblast differentiation, and surfactant production, highlighting a previously unappreciated role for AA-SMO interactions in cardiopulmonary development. Together, these findings provide compelling in vivo evidence that cPLA2α-mediated AA production is a critical modulator of Hedgehog signaling, selectively influencing tissue-specific developmental processes.

Overall, I think this is a significant advance in the field. By utilizing a combination of biochemical, structural, and genetic approaches, this study extends previous cell-based findings to demonstrate a physiologically relevant role for AA-SMO interactions in

cardiopulmonary development. The findings open up new avenues for investigating lipid-mediated modulation of Hh signaling.

Thank you! We appreciate the reviewer's enthusiasm for our work.

I have a few concerns, which I would appreciate the authors addressing.
-The reduced SHH response in the SmoK399A addback cells is an important finding. However, I was surprised that the authors did not conduct a similar analysis with the addition of AA, oxysterols, and cholesterol (as done in Figure 2). Testing these conditions would more directly demonstrate that the K399A mutation indeed disrupts AA-mediated activation of SMO. Can the authors clarify why this was not performed or consider adding this analysis?

The revised manuscript now includes experiments testing for loss of AA responsiveness by SMO^{K399A} using SMO ciliary localization as a read out (new Supplementary Figure 3B-C"). Whereas supplemental AA enhanced ciliary localization of wild type (WT) SMO following 7β,27-DHC, 24,25-EpC, or CHO:MβCD stimulation, it did not enhance ciliary localization of ligand-stimulated SMO^{K399A}. Thus, mutation of the anchoring AA residue attenuates AA-mediated enhancement of sterol/oxysterol-stimulated SMO ciliary enrichment.

-While the SmoD477R mutant serves as a useful control, the reduced surface localization of SmoK399A (Supplementary Figure 2B) raises concerns about whether this mutation affects protein folding rather than just AA binding. If misfolding is an issue, it could explain both the reduced signaling in vitro and the developmental defects observed in vivo. To rule this out, could the authors provide a Western blot from SmoK399A/K399A mice to confirm that total SMO protein levels are comparable to WT?

Thank you for pointing this out. The figure included in the original submission was not reflective of the level of SMO^{K399A} cell surface localization that we typically observe. To rectify this, we replaced the original figures with a new glycosylation analysis (new Figure 5B) and cell surface biotinylation figures in new Figures 5C (representative blot) and C' (quantification of three experiments).

Glycosylation analysis shows similar EndoH and PNGase sensitivity between WT, K399A, D477R, and D477R/K399A SMO proteins. Importantly, WT and SMO^{K399A} proteins show comparable glycosylation patterns (new Figure 5B, lanes 1-3 vs. 4-6). Both have EndoH sensitive (open arrow, ER resident) and EndoH-resistant/PNGase-sensitive (black arrow, post-ER) protein fractions. Consistent with a previous report that the SMO M2 protein does not receive complex glycosylation (PMCID 3044124), we did not observe an EndoH-resistant fraction in M2 and M2/K399A proteins.

Regarding cell surface localization, we ran three independent experiments and found that there is comparable cell surface localization between wild type and each of the SMO variants evaluated (new Figure 5C-C'). Whereas the double mutant proteins had a downward trend in cell surface localization, these changes were not significant

compared to the WT protein. Moreover, each of the double mutants had significantly enhanced signaling activity despite the modestly decreased cell surface localization (Figure 5G, Supplementary Figure 4B-B').

In vivo stability: As suggested by the reviewer, we attempted to evaluate *in vivo* SMO protein levels through western blots of lysates from *Smo*$^{+/+}$ and *Smo*$^{K399A/K399A}$ embryos. Unfortunately, each SMO antibody tested failed to pick up the endogenously expressed protein. To circumvent this issue, we immunoprecipitated SMO from *Smo*$^{+/+}$, *Smo*$^{K399A/+}$ and *Smo*$^{K3999A/K399A}$ embryo lysates and *Smo*$^{-/-}$ MEFs transfected with empty vector or *pcDNA-Smo* as controls. We were able to specifically detect SMO protein using this enrichment strategy. Similar amounts of SMO protein were pulled down from *Smo*$^{+/+}$ *Smo*$^{K399A/+,}$ and *Smo*$^{K3999A/K399A}$ lysates (new Supplementary Figure 5C, lanes 7, 9, and 11). The band observed in these lanes aligns with a band absent in *Smo*$^{-/-}$ MEFs and present in knockout MEFs re-expressing WT SMO protein.

We think that these new results, combined with the normal neural tube patterning observed in *Smo*$^{K399A/K399A}$ mice, provide strong evidence against pronounced misfolding or stability defects of SMO^{K399A} *in vivo*. Ventral neural tube progenitor domain specification is entirely dependent upon SHH signaling through SMO. Progenitor domain specification and cerebellar development are unaltered in mutant mice compared to the control (Figure 6D-E and Supplementary Figure 5F-F'), suggesting that SMO^{K399A} has sufficient stability to signal in this specific context. As such, we think it unlikely that protein misfolding or reduced cell surface localization is responsible for the observed phenotypes *in vitro* or *in vivo*.

- One aspect that I found confusing is the apparent lack of developmental phenotypes in cPLA2α-/- mice. Bonventre et al. (1997) previously reported that cPLA2α-/- mice develop normally, and this finding is supported by the authors' own data (Supplementary Table 1), where the knockout mice appear at expected Mendelian ratios. Given that cPLA2α is proposed to be a key producer of AA, I would have expected the cPLA2α-/- phenotype to be at least as severe, if not more so, than that of SmoK399A/K399A mice. This raises the possibility that AA can still be produced through alternative pathways? Can the authors comment on whether other enzymes compensate for AA production in cPLA2α-/- mice?

Thank you for raising this point. We have added text to lines 462-463 and 469-473 to clarify that compensation by other cPLA2 isoforms is the most likely reason we do not see more pronounced phenotypes in the *cPLA2*$\alpha^{-/-}$ mice. There are at least four additional cytosolic PLA2 genes including cPLA2δ (group IVD), cPLA2ε (group IVE), cPLA2ζ (group IVF), and cPLA2β (group IVB) (DOI 10.1074/jbc.M413711200). All these enzymes contain the essential catalytic domain, and several are expressed in the heart and lungs – especially cPLA2β (please see: www.ncbi.nlm.nih.gov/gene/211429). Dr. Joseph Bonventre and colleagues, who provided us with the *cPLA2α*$^{-/-}$ mice, showed that group IV PLA2 genes are widely expressed and suggested that modified expression of the various isoforms following loss of another allows for redundancy (DOI:

10.1194/jlr.m200298-jlr200). We have provided this potential explanation with the results lines 458-473.

Additionally, is it possible that the phenotypes observed in SmoK399A/K399A mice are due to reduced SMO levels rather than its inability to bind AA?

Please refer to our explanation above for why we do not believe the phenotype results from reduced SMO protein levels.

Is it possible to measure AA levels in cPLA2α-/- cells or tissues? This would help clarify whether AA is still being produced in these mutants.

Unfortunately, this experiment is not feasible. AA is metabolized very rapidly, and we do not currently have the technology to assess AA in developing tissues. However, the ability of other cPLA2 isoforms to produce AA in the absence of cPLA2α suggests that we would not likely observe pronounced reduction of AA in *cPLA2α*$^{-/-}$ mice. We hypothesize that the enhanced phenotype of *Smo*$^{K399A/K399A}$;*cPLA2α*$^{-/-}$ mice compared to the phenotype of either mutation on its own results from 1) loss of SHH-stimulated AA production at the ciliary base (which usually occurs through cPLA2α), and 2) reduced AA binding affinity by the SMO^{K399A} protein.

Reviewer #2 (Remarks to the Author):

This is an exciting and well-written manuscript from Ogden and colleagues. The authors have used cellular assays, computational docking and mouse models to explore the role of arachidonic acid in regulating SMO in vitro and in vivo. Important findings of this research are the unusual tissue-specificity of the mouse mutant phenotype, and the clear dose-responsive synergy between Cholesterol:MβCD and arachidonic acid in GLI reporter assays. Overall the reviewer supports publication; however, there are some major issues that need to be addressed.

Thank you! We appreciate the reviewer's support.

The major concerns are firstly to determine if the differences in arachidonic acid binding pose for Xenopus SMO versus the murine SMO structures have any impact on the importance of the murine K399 residue.

Further computational work (e.g. computational molecular dynamics simulations) could help address this concern.

Thank you for this helpful suggestion. We now include a 20-nanosecond simulation of AA-bound *Xenopus* SMO as new Supplementary Figure 2A-B and new Supplementary Movie 1. This simulation predicts that of all SMO residues in and around the predicted AA pocket, K368, which corresponds to murine SMO K399, forms the strongest bonds to AA over the course of the simulation (new Supplementary Figure 2A). Moreover, K368 makes the most consistent contact with AA out of all nearby SMO residues over

the 20-nanosecond simulation (new Supplementary Figure 2B and new Supplementary Movie 1), increasing confidence that this is a crucial residue for stable SMO-AA interaction.

In addition to the simulation data, we have also included an overlay of reported structures of *Xenopus*, mouse, and human SMO proteins with the relevant lysine shown in green in the new Supplementary Figure 2C. Consistent with conservation of the lysine and its flanking sequence between human, mouse, and *Xenopus* SMO proteins (Figure 3E), we found that all three SMO structures have similar configurations in the region of the relevant lysine (new Supplementary Figure 2C).

Secondly, there is a need to further clarify the effect of the K399A mutant on SMO glycosylation state and stability, and the degree to which this mutant disrupts arachidonic acid binding (total versus partial). Appropriate experiments with purified proteins would address these concerns.

New Figure 5B shows that wild type and SMO^{K399A} proteins show comparable glycosylation patterns (lanes 1-3 vs. 4-6). Both have EndoH-sensitive (open arrow, ER resident) and EndoH-resistant/PNGase-sensitive (black arrow, post-ER) protein fractions. These results, combined with an earlier publication from our lab showing that complex glycosylation is not required for SMO signaling to GLI (PMC4546403), make it unlikely that reduced complex glycosylation of the SMO^{K399A} protein is responsible for reduced signaling activity.

To evaluate cell surface localization of WT and mutant SMO proteins, we have included cell surface biotinylation analyses as the new Figure 5C-C'. SMO cell surface localization is quantified for WT, K399A, E522A, D477R, D477R/K399A, M2, and M2/K399A SMO proteins. Over three independent experiments, we found that there is comparable cell surface localization between WT and each of the SMO variants evaluated. We do note a downward trend in cell surface localization for the double mutant SMO proteins, but these reductions failed to reach significance, and the double mutant proteins remained active in functional assays (new Figure 5F-G, Supplementary Figure 4B-B'). As such, we believe it unlikely that reduced cell surface localization triggers the observed signaling defects.

Specific comments:

- Line 61: "Removal of PTCH1 from ciliary and plasma membranes allows for localized accumulation of sterols that bind SMO to stimulate its PC accumulation and signaling through two distinct effector routes" - Has this been shown previously? The reviewer is not aware of any publication measuring sterol concentrations in the PC.

We rephrased the text for clarification (lines 61-63). The molecular mechanism we were referring to is the proposed ability of PTCH1 to limit free cholesterol (i.e. cholesterol not bound to sphingolipids) in ciliary membrane – possibly by depleting it from the inner and/or outer leaflets of the ciliary membrane. (PMC8654371 and PMC6326742).

The document now reads: *Removal of PTCH from ciliary and plasma membranes eliminates its sterol depleting activity, allowing for localized accumulation of ciliary sterols that bind SMO to stimulate its PC retention and downstream signaling.*

- Line 64: The distinction between canonical and non-canonical HH signaling, and which of the pathways is relevant for this publication is not clear – please specify and clarify in the text.

We expanded discussion of canonical and noncanonical signaling in the introduction. Please see lines 65-75.

- Line 116: SSTR3 is a ciliary marker and is always expressed in the primary cilium, so there will be SSTR3 in the cilium before treatment. For this reason, it is difficult to use SSTR3 as a control for SMO specific dynamic localization to the PC. Another membrane protein that is dynamically regulated within the primary cilium could control for SMO specificity. Maybe clarify this point in the text.

We are sorry that we did not clearly explain our rationale for evaluating SSTR3 in the initial submission. We did not intend SSTR3 to be considered a marker for dynamic GPCR entry into the PC. Rather, we evaluated SSTR3 as an established PC-resident GPCR to show that cPLA2α is unlikely to function as a general regulator of GPCR ciliary entry. We rephrased the text to clarify that SSTR3 is a ciliary-resident GPCR. Additionally, please see our recently published study demonstrating that dynamic, SHH-regulated GPR161 ciliary trafficking is unaltered by cPLA2α inhibition (Figure S1F-F' in PMC11166601). We now note this point and reference this supporting publication in the revised text. The relevant figure panels from the published study are copied below for ease of reference.

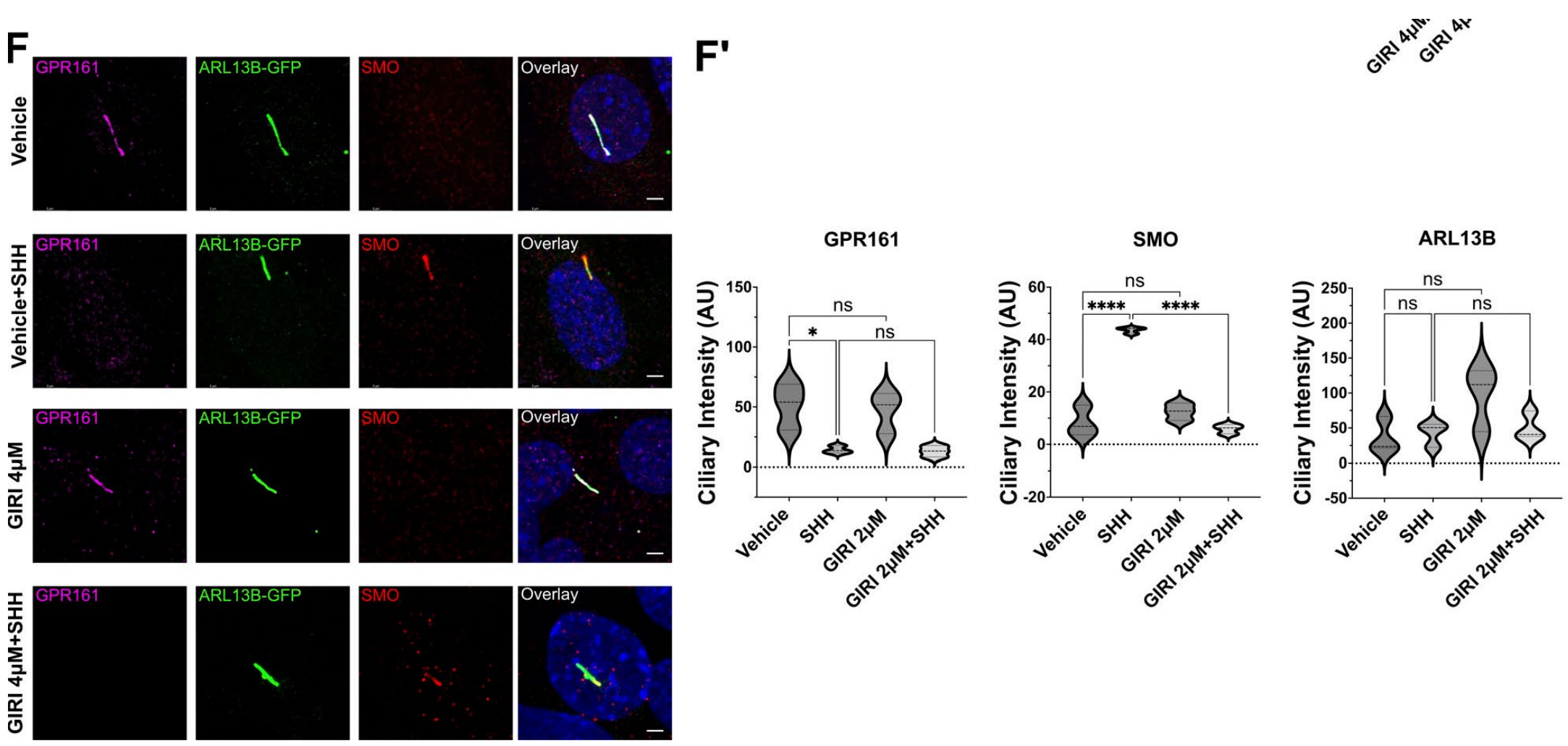

(F) IMCD3 cells were treated with vehicle or GIRI (4 μM) in the absence or presence of SHH. GPR161 is magenta, SMO is red, ARL13B is green and marks PC. Scale bar = 3 μm. (F9) Quantification of ciliary signal intensity for GPR161, SMO, and ARL13B in cells treated with SHH, GIRI, or vehicle control. The experiment was performed twice with 30 cilia imaged per condition per experiment and all data were pooled. For all graphs, significance was determined using a one-way ANOVA. Significance is indicated as follows: *<0.05, **<0.01, ***<0.001, ****<0.0001, and ns, P > 0.05. Data are represented as mean ± SD.

- Figure 1: In microscopy images, it is probably clearer to keep each protein in the same color throughout the figure (and if possible throughout all the figures). It would also be clearer if the DAPI channel is labelled separately, as the blue is tricky to distinguish from the cyan.

We appreciate the reviewer bringing up image accessibility. We have adjusted the colors for consistency across the figures and avoided combining blue and cyan where possible.

- Figure 1: Check if a one-way or a two-way ANOVA is appropriate, given there are two independent variables (SHH treatment, and GIRI treatment).

Thank you for pointing this out. We consulted with the St. Jude Biostatistics Department and have adjusted one-way vs. two-way ANOVA as appropriate throughout the figures. Statistical methods are specified in the figure legends.

- Line 168: To show that GIRI is specifically acting through cPLA2α, the authors might consider a rescue experiment where GIRI inhibition of cPLA2α is combined with exogenous AA treatment.

We have previously shown that exogenous AA can rescue effects of GIRI-mediated cPLA2α inhibition across multiple publications (PMC5520665, PMC11166601). In the first study, we demonstrated that exogenous AA could rescue SHH reporter gene activity following cPLA2α inhibition using another inhibitory compound, MAFP (PMC5520665, see Figure 6B). In another study, we found that the effects of GIRI-mediated cPLA2α inhibition on ciliary length were partially rescued by exogenous AA (PMC11166601, see Figure S1E-E'). Our prior demonstrations that exogenous AA can rescue effects of inhibition of cPLA2α using two different inhibitors give us confidence that the drug effects are on target. We have included these references for the specificity of GIRI in the revised document (lines 133-141).

The relevant figure panels from each publication are copied below for reference.

From: PMC5520665

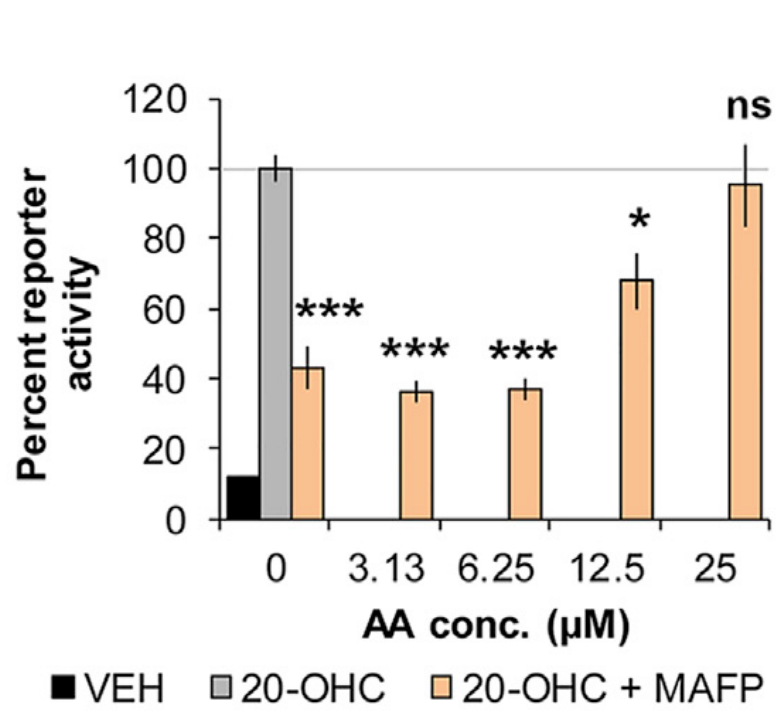

B) Light2 cells were treated with 20(S)-OHC (10 mM) with or without MAFP (5 mM). MAFP-treated cells were treated with arachidonic acid as indicated. The line indicates the baseline 20(S)-OHC-stimulated level, set to 100%. Significance was determined relative to this control using one-way ANOVA.

From: PMC11166601

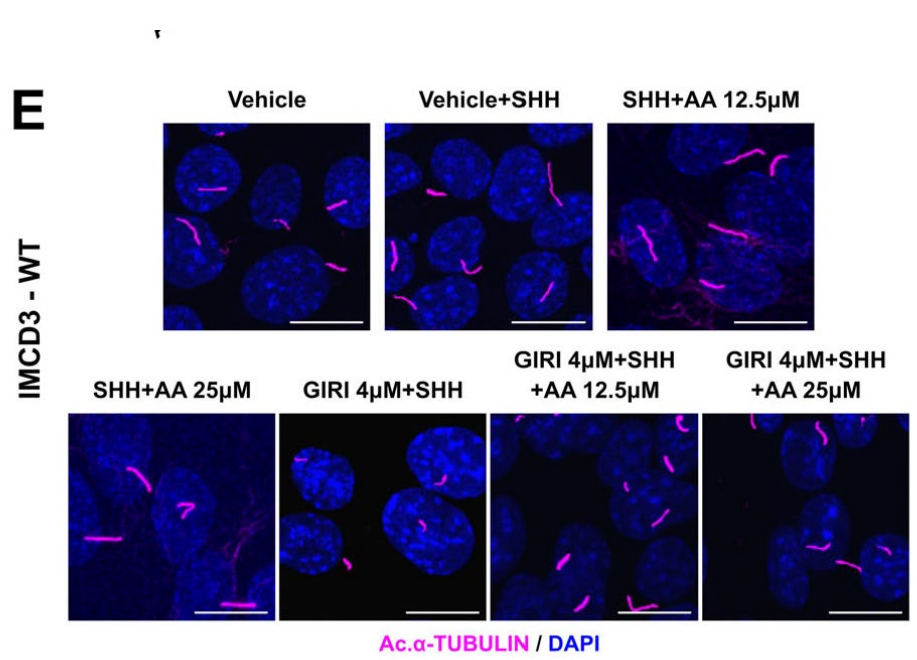

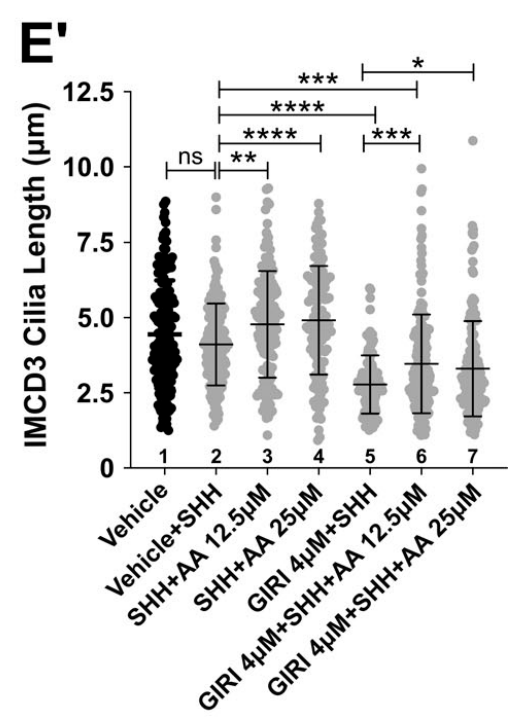

(E) IMCD3 cells were treated with control or SHH-conditioned media in the presence of vehicle or 4 μM GIRI plus increasing concentrations of arachidonic acid (12.5 or 25 μM). The ciliary axoneme is marked by acetylated α-tubulin (magenta) and DAPI (blue) marks the nuclei. Scale bar = 10 μm. (E9) Ciliary length quantification is shown in E9.

- Figure 3: consistent colors, background, and labeling of key amino acids on all panels would help interpretation of this figure. For the red color scheme on the conservation analysis, it is unclear which of the lighter shades represents highest conservation.

We thank the reviewer for this suggestion for improving the interpretability of the figure. Figure 3 is now presented with a uniform background color, consistent ligand and protein coloring schemes, removal of non-polar hydrogens for clarity, and clearer labeling of relevant amino acid residues.

We have also added a key for the conservation analysis in Figure 3E to clarify what the shading indicates. Darker shading is more conserved while lighter shading is less conserved.

- Figure 3: in the text there should be some comment and analysis of why the binding pose of arachidonic acid is different in the murine structure compared to the Xenopus structure, and what implications this has for the expected role of mouse K399 in arachidonic acid binding. This residue should be labelled in the mouse structures.

We thank the reviewer for pointing this out and appreciate the opportunity to clarify our intended meaning. The AA ligand was docked into each protein using the respective crystal structure ligand to define a reference receptor grid. The difference in AA pose (for example between the murine and *Xenopus* structures) in a static crystal structure is due to AA's very large amount of rotatable bonds providing a high degree of flexibility. Unlike more rigid compounds such as cholesterol, AA can adopt many different low-energy conformations depending on the static protein's local environment. Upon analysis of the molecular dynamics simulation suggested by the reviewer, we found that AA quickly adopts a more linear pose which is stable and maintains prominent contact with the K368 residue (new Supplementary Movie 1). We added this discussion to the text (lines 273-295).

- Line 190: To test the effect of AA on TMD cholesterol binding and provide further evidence for the identified salt bridge between K368 and the AA hydroxyl group, it might be useful to carry out some molecular dynamics simulations. These would also confirm the overall binding pose of AA in general.

As noted above, molecular dynamic simulations are included in new Supplementary Figure 2A-B and new Supplementary Movie 1. We thank the reviewer for suggesting the molecular dynamics simulations as we believe they do indeed clarify how AA likely binds to SMO.

- Line 203: The stability assay uses crude membrane extracts. This will not provide any information of potentially destabilized and/or unfolded protein sample. To properly assess the stability of the mutants, the reviewer suggests carrying out the thermostability assay with purified proteins.

To more directly measure SMO-AA binding, we replaced the thermal stability assays with AA bead capture assays in new Figure 4. We found that AA beads can capture SMO-YFP and cyclopamine binding-deficient SMO^{E522A}-YFP proteins similarly and that both SMO-YFP proteins are similarly displaced from the beads by free competitor AA (Figure 4D top and bottom panels and D'-D"). SMO^{K399A} is captured on AA beads less efficiently and is displaced from the beads at lower concentrations of free AA competition (Figure 4D, middle panel). This experiment was repeated three times, and all data were evaluated by densitometry to quantify changes in area under the curve. These data were then analyzed by Drs. Tomi Mori and April Sykes from the St. Jude Biostatistics department. Figures 4D'-D" show that the area under the curve is

significantly reduced by the K399A mutation. Supplementary Figure 2D shows the percent of SMO bound at each dose of free AA competition for each of WT, K399A, and E522A SMO proteins, again highlighting increased sensitivity of K399A to displacement by free AA when compared to WT or E522A SMO proteins. Based on these new results, we are comfortable concluding that K399A mutation destabilizes SMO-AA binding.

- Line 232: "We confirmed that SMOK399A and SMOE522A mutant proteins reached the cell surface like WT SMO-YFP" There is concern about the stability of the SMO-K399A mutant. Although stated, the Western blot analysis clearly indicates different behaviour in glycosylation state compared to wildtype (no clear band for the glycosylated post-ER form). This could be indicative of a protein that is less stable. To prove stability and integrity of the SMO-K399A mutant, the authors could carry out thermostability assays with purified proteins (see above).

Thank you for pointing this out. Unfortunately, the figure included in the original submission was not reflective of the SMO cell surface localization we typically observe. We have included newly repeated glycosylation and cell surface biotinylation experiments that show similar SMO^{WT} and SMO^{K399A} glycosylation patterns, protein levels, and cell surface localization when expressed in cultured cells (new Figure 5B-C'). Moreover, embryo lysates revealed that WT and K399A SMO proteins are present at similar levels *in vivo* (Supplementary Figure 5C; additionally, please see the response to the reviewer's question regarding protein stability above).

Regarding glycosylation status, our previously published work indicates that N-linked glycosylation is not required for murine SMO signaling to GLI transcriptional effectors (PMC4546403, see Figures 4-6). Additionally, please note that SMO-M2, which is constitutively active and signals at a very high level, had reduced protein levels and lacked normal glycosylation when compared to the WT SMO protein (Figure 5B-C', Supplementary Figure 4B-B').

In vivo, $Smo^{K399A/K399A}$ mice demonstrate normal neural tube patterning. Ventral neural tube progenitor domain specification is entirely dependent upon SHH signaling through SMO. Progenitor domain specification and cerebellar development were unaltered in mutant mice compared to control (Figure 6D-E and Supplementary Figure 5E-F'), suggesting that SMO^{K399A} has sufficient protein stability to signal in this context. We propose that these observations argue against a pronounced stability or cell surface localization defect *in vivo*.

- Line 269: 'Double mutant' might be a clearer term than 'compound mutant', to avoid confusion with compounds/ligands.

This is a good point and we have made this suggested change throughout the revised text.

- Line 274: Analysis of the ciliary localization of these double mutants (as was completed for the single mutants) might provide further insight into these results.

We performed the suggested experiment and have added the results in new Figure 5F. Like the observed effects on *Gli1* expression, we found that the D477R mutation rescued SMO ciliary localization when combined with the K399A mutation. Intriguingly, combining the M2 mutation with the K399A mutation did not rescue SMO ciliary localization, but robustly rescued transcriptional activity (Figure 5G, Supplementary Figure 4B-B'). This is consistent with the high signaling activity conferred by the M2 mutation. These results are discussed in lines 428-435 and 654-666.

-Line 304: "may represent phenotypic "escapers" with compensatory signaling activity". Some explanation is needed to clarify this hypothesis (rather than SmoK399A/K399A simply being epistatic over Pla2α-/- with respect to the size reduction phenotype, because SMO is acting downstream of PLA2α in the signalling pathway).

We apologize that our explanation was not clear here. We meant to suggest that the mice were *cPla2α*$^{-/-}$ phenotypic escapers, not *Smo*$^{K399A/K399A}$ mutant escapers. We think the surviving *Smo*$^{K399A/K399A}$*;cPla2α*$^{-/-}$ mice likely have upregulation of other *cPla2* isoforms, as has been described in the literature (DOI: 10.1038/37635). We have rephrased lines 470-473 to clarify our point:

We hypothesize that these surviving animals may represent phenotypic cPLA2α$^{-/-}$ *escapers with compensatory phospholipase activity occurring through upregulation of other cPLA2 isoforms. This response has been documented in for cPla2α*$^{-/-}$ *mice that lack overt phenotypes (DOI: 10.1038/37635).*

-Line 299: Would we expect the double mutant to phenocopy rather than exacerbate the single mutant if there is loss of function in the same pathway? The authors might want to mention here that SMO and PLA2a loss of function could have distinct effects that only synergize at the level of the whole mouse's development (as they comment on in the discussion, line 446).

The reviewer has raised a good point. We edited the paragraph beginning at line 458 to clarify that we hypothesized that enhancement would likely occur because we are compromising AA-SMO effects in two ways when combining *Smo*$^{K399A/K399A}$ with *cPLA2α* knockout: 1). cPLA2α loss is predicted to eliminate SHH-stimulated production of AA (PMC5520665 and PMC11166601), and 2). K399A mutation reduces SMO responsiveness to AA that is produced by other sources (i.e., other *cPla2* isoforms).

- Line 465: Given several experiments in this publication suggest that arachidonic acid binding is required for SHH-mediated SMO activity, perhaps some further comment should be made about whether arachidonic acid is considered a necessary ligand or a dispensable allosteric modulator.

We think it is a dispensable allosteric modulator – at least in the context of the neural tube. Genetic experiments suggest it may be more important in organs that require

discrete, temporal bursts in SHH signaling, like the heart and lungs. We have expanded discussion of this possibility in the text lines 668-684.

- General comment: It would be informative if experiments characterizing primary cilia would include an analysis of the lengths of the imaged primary cilia, to check whether mutations and/or drug treatments have any major effects on cilium integrity. This is especially the case given the in vivo RNA-seq data for the K399A/K399A embryos indicates disruption of genes regulating PC integrity (line 345).

Ciliary length measurements are shown in new Supplementary Figures 1B', C' and 3A'. These results are discussed in lines 165-174 and 368-370.

Reviewer #3 (Remarks to the Author):

This study investigates how arachidonic acid (AA) binds to a putative allosteric site in the 7TM domain of Smoothened (SMO) and its implications for Hedgehog signaling. The authors build on previous work from the Ogden lab (e.g., Arensdorf et al, Cell Reports 2017) by mapping this AA-binding site and testing its functional importance in cell-based and in vivo models. The identification of a specific SMO mutation (K399A) that disrupts AA binding is intriguing, the in vivo analysis of this knock-in allele provides intriguing developmental phenotypes, and the integration of structural, functional, and in vivo assays is impressive. Unfortunately, however, significant technical and interpretational issues, particularly regarding the thermal shift assays, ciliary localization assays, and functional assays, weaken the strength of the conclusions. In addition, while the mouse mutant supports the functional importance of this allosteric site for some aspects of Hedgehog signaling, the biochemical and cultured cell data do not definitively map it as an AA-specific binding site. The same site is also used by 24,25-epoxycholesterol and potentially other endogenous SMO ligands, which the authors have not ruled out.

Major Concerns

1. Lack of strong evidence for AA binding specificity
-The thermal shift assay (Fig 4) used to infer AA binding to SMO lacks proper controls and standardization, making it difficult to draw conclusions from the data.

-The data are fit with a curve using GraphPad prism, but no details are provided on the fitting method –many individual data points deviate significantly from the fitted curve, and the shapes of the curves are vastly different for the wild-type and mutant SMO proteins. This makes it very difficult to evaluate the data.

-The actual thermal shift in SMO upon AA binding appears modest, raising concerns that the effect of AA may be very modest and that the assay may not be detecting a meaningful, specific binding interaction.

Per the reviewer's suggestion (below), we replaced the thermal stability assay with SMO-AA bead binding assays in new Figure 4. This approach provides a more direct assessment of AA binding ability and specificity of SMO proteins. We included tests for binding specificity by using specific and nonspecific free competitors and assessing binding of another GPCR, SSTR3, to AA beads. Discussion of these results begins at line 299 of the revised document. Additional considerations for specific reviewer comments are discussed in more detail below.

-The K399A mutant shows a significantly altered thermal profile compared to wild-type SMO, suggesting potential structural instability rather than a specific loss of AA binding.

Given the indirect nature of measuring SMO small molecule binding and stability in bulk cell extracts we replaced these experiments with the AA bead binding assay (new Figure 4). We do not think that the K399A protein exhibits pronounced structural instability due to it showing similar protein levels, glycosylation status, and cell surface localization to WT (new Figure 5B-C'). Moreover, the ability of SMO^{K399A} to translocate to the primary cilium like the WT protein following SAG or cyclopamine treatment and to be activated by SAG (Figure 5D-D' and Supplementary Figure 4A-B') suggests that structural integrity is sufficient for SMO^{K399A} to be responsive to small molecule binding in the upper transmembrane binding pocket. As such, we are comfortable concluding that SMO^{K399A} is compromised in its ability to stably bind AA due to mutation of the anchor and not collapse of the pocket or pronounced instability.

-The cyclopamine-resistant E518A mutant still shows a substantial shift upon cyclopamine treatment (comparable in magnitude to the effect of AA on wild-type SMO), further calling into question whether the assay is measuring specific ligand interactions.

As stated above, we replaced the thermal shift assays with more direct AA bead binding assays (new Figure 4). SMO^{E522A} behaved similarly to the wild type protein in bead binding assays (new Figure 4D-D").

-No negative control is shown for AA (i.e., a non-AA-binding GPCR, such as SSTR3), which is necessary to show that the effect of AA on SMO in this assay reflects a specific binding event (as opposed to a nonspecific effect on SMO or on the assay in general).

We included SSTR3 as a negative control in the new AA bead binding assays (new Figure 4B). SSTR3 is not captured on AA beads, supporting the specificity of this assay.

-No raw data are presented for the assay – only analyzed, quantified data. Raw data should be shown for the blots used in this analysis.

All thermal stability assays have been replaced with bead binding assays. Representative western blots are shown for all data presented in this figure (new Figure 4D-D"). Replicates will be available with source data that will be uploaded to the journal if/when the manuscript is accepted for publication.

If the goal is to demonstrate AA binding disruption, it would be much more effective and convincing to use direct ligand binding assay, such as the bead-based AA binding assay they developed in their 2017 study. Such an assay is more direct and interpretable than the thermal shift assay, which is an indirect proxy for binding.

We appreciate the reviewer's suggestion and have incorporated it (see above responses).

2. Issues with GLI reporter assays and cellular experiments
-The GLI reporter assays (Fig. 2F, 2G) show that AA synergizes with SMO agonists, but the authors do not test whether AA enhances SMO ciliary localization in response to these agonists —a critical mechanistic prediction of their model. Instead, they only test whether blocking AA metabolism (via GIRI) reduces SMO localization.

Thank you for the suggestion. We have included these experiments as new Figures 2C-C" and new Supplementary Figure 3, which shows effects of AA treatment on WT and K399A SMO protein ciliary enrichment following stimulation with 7β,27-DHC, 24(S),25-EpCHO, and MβCD-CHO. AA enhanced ligand-stimulated SMO ciliary enrichment for the WT protein, but it did not augment ciliary localization of the AA-binding deficient SMO^{K399A} protein.

-The Firefly/Renilla luciferase ratio is used to measure GLI reporter activity, but given that AA is highly hydrophobic and is being used at relatively high concentrations (15 micromolar) over a 36-hour incubation, it could cause unintended effects on cell viability or on Renilla luciferase activity, artifactually increasing the Firefly/Renilla ratio. The authors should show raw Renilla readings from their assays, perhaps in a supplementary figure, to exclude this possibility.

Thank you for bringing this issue to our attention! We checked the raw data and found that AA did not impact the Renilla normalization control at any concentration tested when added on its own. However, combining cholesterol or oxysterols with AA did reduce Renilla activity at higher concentrations, which skewed our normalized data and lowered confidence about the fitness of the cells. To eliminate these complications, we replaced all luciferase reporter assays presented in the manuscript with qPCR assays for endogenous *Gli1* induction. We confirmed that neither sterol nor AA impacted expression of the normalization control gene *Btf3* in any of the experiments we performed. Control graphs are included below for the reviewer's reference.

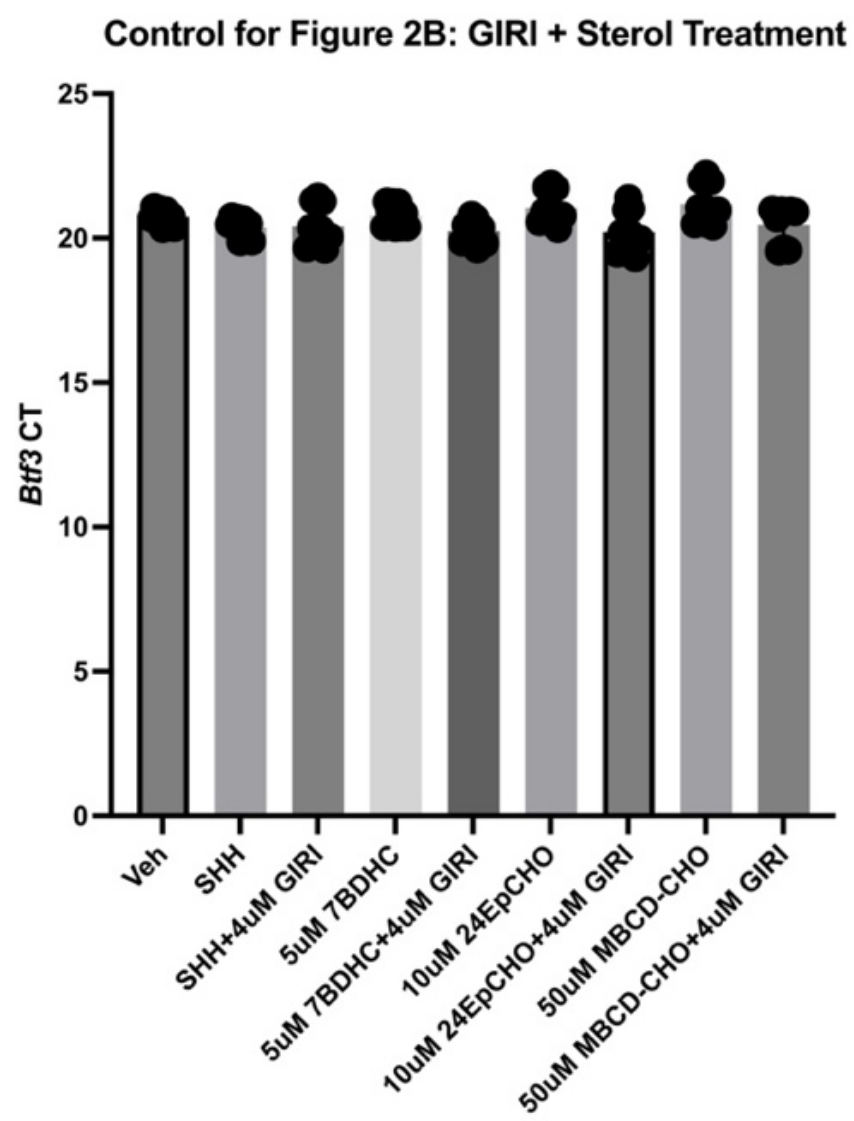

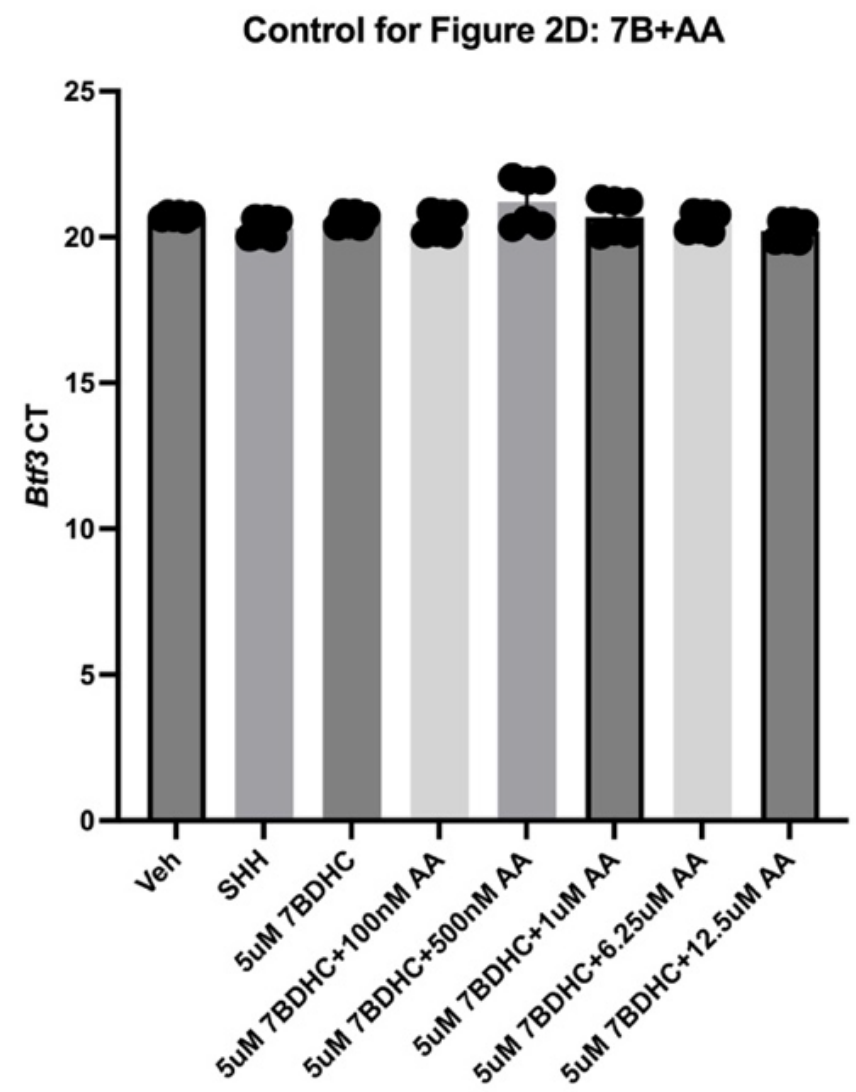

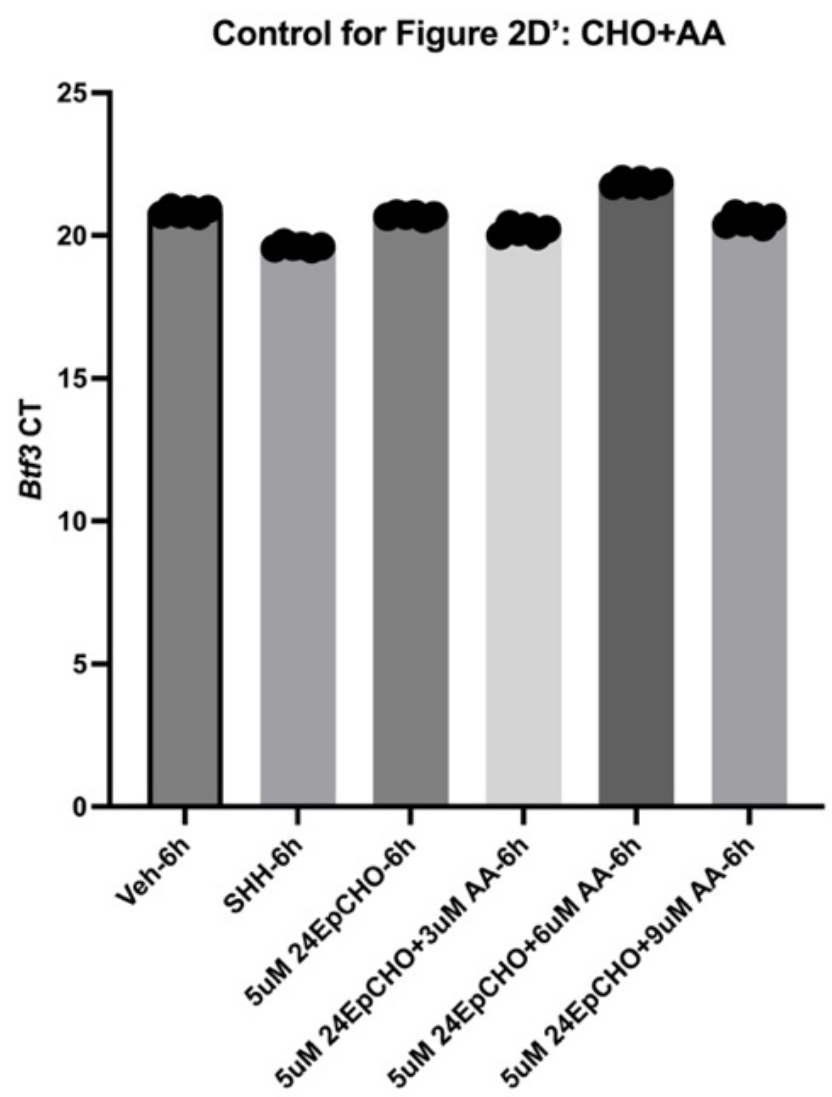

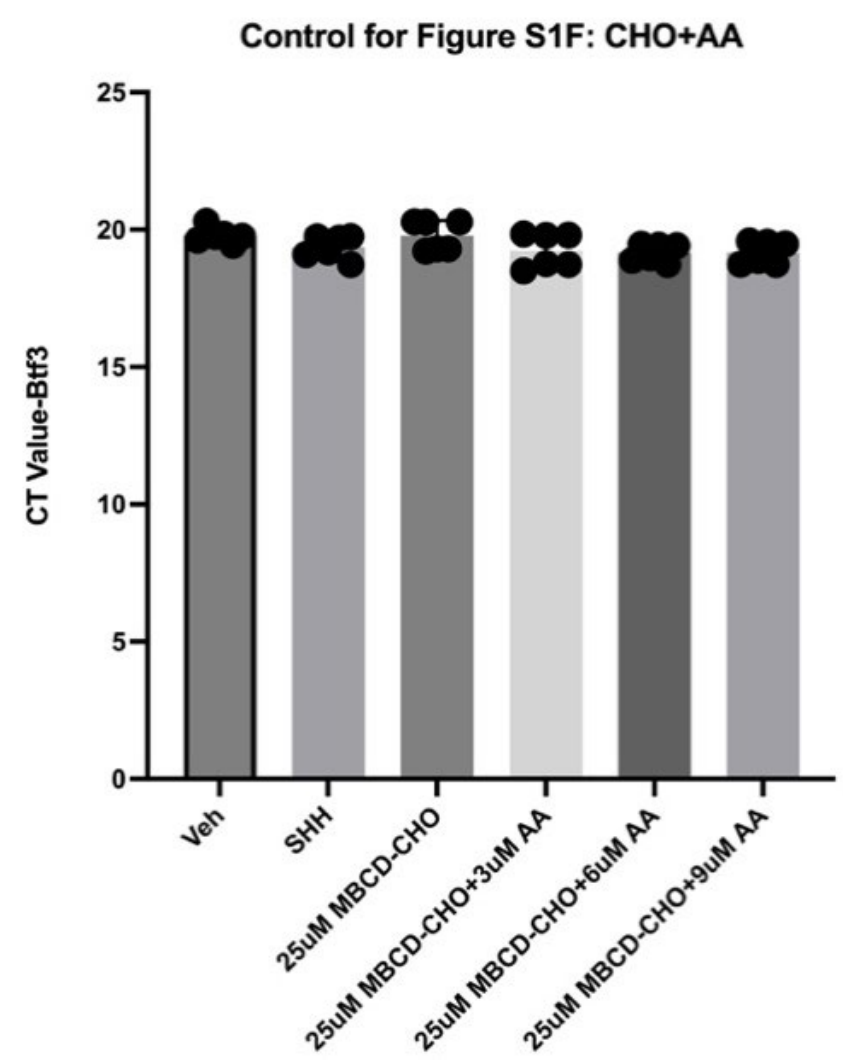

During our trouble shooting, we found that overnight treatments with sterol or oxysterol + AA negatively impacted cell viability. This was especially pronounced when combining AA with MβCD as a CHO delivery mechanism. However, since SMO ciliary translocation occurs rapidly after ligand stimulation, and qPCR is a more sensitive assay than the luciferase reporter, we were able to detect changes in SMO PC retention and transcriptional induction by ~2-6 hours post treatment. Due to complications of combining AA with MβCD that are outlined in the text (lines 236-250), we were unable to assess the effect of AA on CHO:MβCD-mediated target gene induction. However, we were able to assess SMO ciliary localization at shorter time points (~2 hours post treatment) and observed that CHO:MβCD-stimulated SMO ciliary enrichment is augmented by addition of AA (new Figure 2C'). We believe that this result, combined with the observation that treatment of CHO:MβCD-stimulated cells with the cPLA2α

inhibitor GIRI lowers *Gli1* induction (new Figure 2B), allows us to conclude that AA can augment CHO-stimulated SMO activity.

Importantly, qPCR analyses of sterol-induced effects on *Gli1* induction allowed us to draw new and interesting conclusions about sterol and AA modulation of SMO activation and signaling. The key new takeaways from our new ciliary localization and transcriptional analyses are:

- 7β,27-DHC, which is reported to be restricted to binding the CRD (PMC6503851), activated SMO to levels near what is seen following SHH stimulation.
- AA augments 7β,27-DHC-stimulated SMO ciliary localization and *Gli1* induction to SHH-like levels (new Figure 2C).
- CHO:MβCD, which binds the CRD and deep in the 7TM domain, activated less robustly than SHH, but did show enhanced SMO ciliary retention when AA was added (new Figure 2C').
- 24,25-EpCHO, which can bind SMO through the CRD, upper 7TM domain, and IC loops (PMC6503851), stimulated SMO ciliary enrichment and *Gli1* induction less robustly than SHH.
- The effects of AA on PC enrichment and signaling by 24(S),35-EpCHO-activated SMO were less pronounced than the augmentation observed for 7β,27-DHC and CHO:MβCD-stimulated SMO (Figure 2C" and D').

Altogether, these results suggest that AA may be a preferred allosteric enhancer for ligands that bind through the CRD or deeper in the 7TM core. Please see text lines 228-234 and 624-653 detailed discussion of this point.

-The authors claim that SMO K399A undergoes normal glycosylation and surface expression, but in Supp Fig 2A-B, the K399A mutant exhibits reduced glycosylation and markedly reduced surface expression. This discrepancy should be addressed, as it complicates interpretation of the in vivo and signaling defects.

We apologize for including blots that were not reflective of what we typically observe for SMO protein stability. This concern was addressed for Reviewers 1 and 2, above. The response has been copied here for ease:

We have included newly repeated glycosylation and cell surface biotinylation experiments that show similar SMO^{WT} and SMO^{K399A} glycosylation patterns, protein levels, and cell surface localization when expressed in cultured cells (new Figure 5B-C'). Moreover, embryo lysates revealed that WT and K399A SMO proteins are present at similar levels *in vivo* (Supplementary Figure 5C; additionally, please see the response to the reviewer's question regarding protein stability above).

Regarding glycosylation status, our previously published work indicates that N-linked glycosylation is not required for murine SMO signaling to GLI transcriptional effectors (PMC4546403, see Figures 4-6). Additionally, please note that SMO-M2, which is

constitutively active and signals at a very high level, had reduced protein levels and lacked normal glycosylation when compared to the WT SMO protein (Figure 5B-C", Supplementary Figure 4B-B').

In vivo, *Smo*$^{K399A/K399A}$ mice demonstrate normal neural tube patterning. Ventral neural tube progenitor domain specification is entirely dependent upon SHH signaling through SMO. Progenitor domain specification and cerebellar development were unaltered in mutant mice compared to control (Figure 6D-E and Supplementary Figure 5F), suggesting that SMO^{K399A} has sufficient protein stability to signal in this context. We propose that these observations argue against a pronounced stability or cell surface localization defect *in vivo*.

-Figs 5E and E' – the GLI1 western blot shows very low signal to noise, with only 2 fold induction over background (and large error bars) for wild-type SMO. This makes it very difficult to assess the functional effects of the mutants. GLI transcriptional reporter assay, as in Fig 2, or a Gli1 qPCR measurement, would be a much more accurate and quantitative way to assess signaling activity of the SMO mutants.

Thank you for this suggestion. We have included qPCR analysis of signaling activity of SMO^{K399A} vs. the constitutively active SMO mutants as new Figure 5G. We found that SMO^{K399A} had significantly less transcriptional activity than all other SMO proteins evaluated. Importantly, combining D477R or M2 mutations with the K399A mutation rescued ciliary localization and signaling activity, consistent with active SMO mutants not requiring AA binding for high-level activity (new Figure 5F-G). Please see text lines 343-370 for discussion of these results.

3. Issues with interpretation of in vivo findings
-The K399A knock-in mouse reveals that the shallow 7TM site is important for SMO function, but the authors overstate the role of AA as the primary endogenous ligand for this site. This site is also used by other sterol-like molecules, including 24,25-epoxycholesterol, which has not been ruled out.

We have included new experiments to address this concern:

1. We analyzed ciliary enrichment of WT and K399A SMO proteins following treatment with 7β,27-DHC, 24(S),25-EpCHO, or CHO:MβCD to evaluate whether K399A mutation would compromise SMO responsiveness to either oxysterol (new Supplementary Figure 3). Importantly, we found that SMO^{K399A} is responsive to 7β,27-DHC, 24(S),25-EpCHO, and CHO:MβCD (new Supplementary Figure 3C'-C") suggesting that the K399A mutant can still bind and respond to these sterol/oxysterol ligands. These results are consistent with published data indicating that the 24(S)25-EpCHO pocket is slightly deeper in the transmembrane bundle than the predicted AA binding pocket that overlaps with the cyclopamine/SAG pocket (DOI: 10.1038/s41586-019-1286-0, see Figure 3D and Extended Data Figure 6F of that publication). Moreover, AA augmented

7β,27-DHC and 24(S),25-EpCHO-stimulated ciliary localization for the WT protein, but not for the K399A mutant, further suggesting that AA-mediated allostery occurs through the K399A-anchored upper 7TM pocket.

2. AA bead binding assays shown in new Figure 4 support that SMO can be captured on AA beads in the presence of either 7β,27-DHC (Figure 4A-B) or 24,25-EpCHO (Figures 4A and C). Given the specific concern with 24,25-EpCHO, we included a broader competition curve against this oxysterol and found that it could not compete SMO off the AA beads (Figure 4C, lanes 3-5 and C'). Similar doses of free AA significantly competed SMO off the AA beads (lanes 6-7), suggesting that AA and the oxysterol do not engage in competitive SMO binding.

   Unfortunately, we could not directly test 24,25-EpCHO binding to WT or SMO^{K399A} proteins in the presence or absence of AA using bead binding assays because we could not find a company that was willing or able to synthesize clickable 24(S),25-EpCHO. Nevertheless, we think that the ability of 24,25-EpCHO to simulate SMO^{K399A} ciliary enrichment and its inability to compete WT SMO off AA beads argues against the theory the K399A pocket facilitates 24,25-EpCHO SMO activation or binding.

However, because cPLA2α is the specific cytosolic phospholipase activated by SHH, we reasoned that reducing the SHH-stimulated AA pool in a mouse expressing a SMO protein that binds the fatty acid less efficiently might worsen the phenotype. In line with this prediction, we generated only 2 $Smo^{K399A;K399A}$;$cPla2a^{-/-}$ mice out of 268 genotyped. As such, we think it is safe to conclude that the enhanced neonatal lethality observed with $Smo^{K399A/K399A}$;$cPLA2\alpha^{-/-}$ is a reliable indicator of a combinatorial effect from reduced AA production and impaired AA-SMO binding downstream of SHH. We have include these considerations in the results lines 457-472.

-The cPLA2 knockout is claimed to synergize with SMO K399A, yet the authors never analyze the double mutant in the heart or lung, which are the tissues most affected by SMO K399A. Without this, the claim of synergy between cPLA2 and SMO K399A is unsupported and could simply be independent, unrelated effects.

We tried to do the experiments requested. Unfortunately, we were unable to assess surfactant production or monitor adult lung function because the double mutant animals are so rarely obtained. As stated in the text, we recovered only **2** $Smo^{K399A/K399A}$;$cPLA2\alpha^{-/-}$ mice out of **over 268** genotyped (Supplementary Table 1). We set multiple crosses while completing revision experiments but failed to obtain any double mutant mice of appropriate ages to analyze lung function. We did manage to obtain two double mutant embryos of the appropriate age to evaluate heart development, but tragically, these embryos were damaged upon shipping to our collaborator for analysis. It took over 6 months to obtain just these two embryos, which makes it unfeasible to get enough embryos to properly evaluate cardiopulmonary phenotypic exacerbation.

However, our inability to collect *Smo*$^{K399A/K399A}$*;cPla2*$\alpha^{-/-}$ embryos combined with the significantly reduced numbers of live double mutant mice obtained (2 out of 268) strongly suggests exacerbation of the *Smo*$^{K399A/K399A}$ phenotype by combinatorial loss of cPLA2α. We hypothesize that the reason other cPLA2 isoforms do not compensate in the background of SMO^{K399A} mutation is likely because cPLA2α is the specific isoform induced downstream of SHH to produce AA for SMO binding. Thus, eliminating SHH-stimulated AA production in an animal expressing the SMO^{K399A} mutant, which binds AA poorly, worsens the viability phenotype of *Smo*$^{K399A/K399A}$. These results are discussed in lines 458-473.

REVIEWERS' COMMENTS

Reviewer #1 (Remarks to the Author):

Thank you to the authors for addressing my comments. I continue to believe this is a great paper and will be a significant contribution to the field. I fully support its publication.

Thank you!

Reviewer #2 (Remarks to the Author):

The authors have now addressed all of my comments and concerns. With the inclusion of the new data and the revisions made to the manuscript, the work has improved substantially and the conclusions are now more convincing. I therefore recommend the manuscript for publication.

Thank you!

Reviewer #3 (Remarks to the Author):

The authors have addressed my concerns, and I now support publication.

Thank you!
