# Supplementary material for: Receptor allostery promotes context-specific Sonic Hedgehog signaling during embryonic development
Source: Nat Commun. 2026 Jul 24;17:9040. doi: 10.1038/s41467-026-75918-5 (PMC13507325; doi:10.1038/s41467-026-75918-5)
Supplement: Supplementary file 3 [file 41467_2026_75918_MOESM3_ESM.pdf]

File Name: Supplementary Movie 1

Description: Molecular Dynamics simulation of Arachidonic Acid-bound SMO. Arachidonic Acid (AA, green) was docked onto Xenopus SMO (PDB: 6D32), and the interaction was simulated over the course of 20 nanoseconds. Throughout the simulation, SMO K368 (blue) formed the strongest bonds to AA. AA adopted flexible conformations within the binding pocket while maintaining consistent contact with K368.
