# Supplementary material for: Receptor allostery promotes context-specific Sonic Hedgehog signaling during embryonic development
Source: Nat Commun. 2026 Jul 24;17:9040. doi: 10.1038/s41467-026-75918-5 (PMC13507325; doi:10.1038/s41467-026-75918-5)
Supplement: Supplementary file 4 [file 41467_2026_75918_MOESM5_ESM.pdf]

nature portfolio

Corresponding author(s): Stacey K. Ogden

Last updated by author(s): 7-1-26

# Reporting Summary

Nature Portfolio wishes to improve the reproducibility of the work that we publish. This form provides structure for consistency and transparency in reporting. For further information on Nature Portfolio policies, see our Editorial Policies and the Editorial Policy Checklist.

## Statistics

For all statistical analyses, confirm that the following items are present in the figure legend, table legend, main text, or Methods section.

| n/a | Confirmed | |
|---|---|---|
| ☐ | ☒ | The exact sample size (*n*) for each experimental group/condition, given as a discrete number and unit of measurement |
| ☐ | ☒ | A statement on whether measurements were taken from distinct samples or whether the same sample was measured repeatedly |
| ☐ | ☒ | The statistical test(s) used AND whether they are one- or two-sided<br>*Only common tests should be described solely by name; describe more complex techniques in the Methods section.* |
| ☒ | ☐ | A description of all covariates tested |
| ☐ | ☒ | A description of any assumptions or corrections, such as tests of normality and adjustment for multiple comparisons |
| ☐ | ☒ | A full description of the statistical parameters including central tendency (e.g. means) or other basic estimates (e.g. regression coefficient) AND variation (e.g. standard deviation) or associated estimates of uncertainty (e.g. confidence intervals) |
| ☐ | ☒ | For null hypothesis testing, the test statistic (e.g. *F*, *t*, *r*) with confidence intervals, effect sizes, degrees of freedom and *P* value noted<br>*Give P values as exact values whenever suitable.* |
| ☒ | ☐ | For Bayesian analysis, information on the choice of priors and Markov chain Monte Carlo settings |
| ☒ | ☐ | For hierarchical and complex designs, identification of the appropriate level for tests and full reporting of outcomes |
| ☒ | ☐ | Estimates of effect sizes (e.g. Cohen's *d*, Pearson's *r*), indicating how they were calculated |

*Our web collection on statistics for biologists contains articles on many of the points above.*

## Software and code

Policy information about availability of computer code

| | |
|---|---|
| Data collection | N/A |
| Data analysis | All statistical analyses were performed using GraphPad Prism v10-11.0.2, except the Area Under the Curve statistical analyses which were performed using SAS 9.4 and the Supplementary Table Fisher's Exact Test which was performed using Python 3.14.2. |

For manuscripts utilizing custom algorithms or software that are central to the research but not yet described in published literature, software must be made available to editors and reviewers. We strongly encourage code deposition in a community repository (e.g. GitHub). See the Nature Portfolio guidelines for submitting code & software for further information.

## Data

Policy information about availability of data

All manuscripts must include a data availability statement. This statement should provide the following information, where applicable:

- Accession codes, unique identifiers, or web links for publicly available datasets
- A description of any restrictions on data availability
- For clinical datasets or third party data, please ensure that the statement adheres to our policy

The RNAseq dataset was deposited into GEO with an accession number of GSE283994 [https://www.ncbi.nlm.nih.gov/geo/query/acc.cgi]. Source data are provided with this paper. Raw data will be available upon request to the corresponding author Stacey.Ogden@stjude.org.

## Research involving human participants, their data, or biological material

Policy information about studies with human participants or human data. See also policy information about sex, gender (identity/presentation), and sexual orientation and race, ethnicity and racism.

| | |
|---|---|
| Reporting on sex and gender | N/A |
| Reporting on race, ethnicity, or other socially relevant groupings | N/A |
| Population characteristics | N/A |
| Recruitment | N/A |
| Ethics oversight | N/A |

Note that full information on the approval of the study protocol must also be provided in the manuscript.

# Field-specific reporting

Please select the one below that is the best fit for your research. If you are not sure, read the appropriate sections before making your selection.

- [x] Life sciences
- [ ] Behavioural & social sciences
- [ ] Ecological, evolutionary & environmental sciences

For a reference copy of the document with all sections, see nature.com/documents/nr-reporting-summary-flat.pdf

# Life sciences study design

All studies must disclose on these points even when the disclosure is negative.

| | |
|---|---|
| Sample size | Samples sizes were chosen to include at least 2 biological replicates with at least 3 technical replicates per experiment. At least 40-50 individual cells were included for analyses that required individual measurements. All mouse experiments utilized male and female animals or embryos with cohorts of 4 or more animals per genotype per experiment. |
| Data exclusions | Three data points were excluded from lung surfactant analysis following Prism outlier analysis (1 of 6 for WT E18.5-19.5, 1 of 4 for K399A p0, and 1 of 9 for K399A E18.5-19.5). |
| Replication | At least 2 biological replicates were included in the analyses, and all data were represented in the figures. |
| Randomization | There was no randomization needed for any of the experiments. |
| Blinding | Blinding was conducted for lung and heart pathological analyses. qPCR analysis on lung samples were conducted in a blinded manner, prior to genotyping. |

# Reporting for specific materials, systems and methods

We require information from authors about some types of materials, experimental systems and methods used in many studies. Here, indicate whether each material, system or method listed is relevant to your study. If you are not sure if a list item applies to your research, read the appropriate section before selecting a response.

### Materials & experimental systems

| n/a | Involved in the study | |
|---|---|---|
| [ ] | [x] | Antibodies |
| [ ] | [x] | Eukaryotic cell lines |
| [x] | [ ] | Palaeontology and archaeology |
| [ ] | [x] | Animals and other organisms |
| [x] | [ ] | Clinical data |
| [x] | [ ] | Dual use research of concern |
| [x] | [ ] | Plants |

### Methods

| n/a | Involved in the study | |
|---|---|---|
| [x] | [ ] | ChIP-seq |
| [x] | [ ] | Flow cytometry |
| [ ] | [x] | MRI-based neuroimaging |

## Antibodies

| | |
|---|---|
| Antibodies used | Smoothened (Clone E-5, Santa Cruz Biotechnology, sc-166685), acetyl-alpha-Tubulin (D20G3, Cell Signaling Technology, 5335), ARL13B (BiCell Scientific, 90413), p-cPLA2 (Cell Signaling Technology, 2831), Gli1 (L42B10, Cell Signaling Technology, 2643), IFT88 (Proteintech, 13967-1-AP), PDGFRa-biotin (R&D Systems, BAF1062), PAX6 (DSHB, pax6), OLIG2 (Millipore, AB9610), FOXA2 (Abcam, ab108422), NKX2.2 (Novus, NBP1-82554), gamma-Tubulin (Abcam, ab11316), KIF5B (Abcam, ab167429), B-actin (AC-15, Santa Cruz |

| | |
|---|---|
| | Biotechnology, sc-69879), GAPDH (Cell Signaling Technology, 5174), GFP (Rockland, 600-401-215), Goat anti-Mouse IgG (H+L) AF488 (ThermoFisher Scientific A-11029), Goat anti-Rabbit IgG (H+L) AF 488 (Thermo Fisher Scientific, A-11034), Goat anti-Mouse IgG AF555 (H+L) (Thermo Fisher Scientific, A21424), Goat anti-Rabbit IgG (H+L) (Thermo Fisher Scientific, A-21429), Goat anti-Mouse IgG (H+L) AF647 (Thermo Fisher Scientific, A-21236), Goat anti-Rabbit IgG (H+L) (ThermoFisher Scientific, A21245), Peroxidase-AffiniPure Donkey Anti-Mouse IgG (H+L) (Jackson ImmunoResearch Labs, 715-035-151), Peroxidase-AffiniPure Donkey Anti-Rabbit IgG (H+L) (Jackson ImmunoResearch Labs, 711-035-152), HRP-conjugated rat anti mouse light chain (Southern Biotech, 1170-05), |
| Validation | All antibodies used are commercially available and have been validated. Secondary antibody-only controls were performed to ensure specificity of primary antibodies, where appropriate. Proper negative and positive controls were included with each experiment to ensure specificity of antibody recognition. |

## Eukaryotic cell lines

Policy information about cell lines and Sex and Gender in Research

| | |
|---|---|
| Cell line source(s) | Homo Sapiens: HEK293T (ATCC; RRID: CVCL_1926), Mus musculus: NIH3T3 (ATCC; RRID: CVCL_0594), Mus musculus: NIH3T3 Flp-In (Thermo Fisher Scientific R76107), Mus musculus: Smo-/- NIH3T3 Flp-In (DOI: /10.7554/eLife.61432),Mus musculus: WT Smo-YFP NIH3T3 Flp-In (This paper), Mus musculus: K399A SMO-YFP NIH3T3 Flp-In (This paper), Mus musculus: E522A SMO-YFP NIH3T3 Flp-In (This paper), Mus musculus: D477R SMO-YFP NIH3T3 Flp-In (This paper), Mus musculus: K399A/D477R SMO-YFP NIH3T3 Flp-In (This paper), Mus musculus: M2 SMO-YFP NIH3T3 Flp-In (This paper), Mus musculus: M2/K399A SMO-YFP NIH3T3 Flp-In (This paper) |
| Authentication | All cell lines were routinely validated using functional assays and western blotting as appropriate. Cell lines are routinely subjected to STR Profiling using the ATCC authentication service. |
| Mycoplasma contamination | All cell lines were regularly tested for mycoplasma contamination using MycoAlert (Lonza). |
| Commonly misidentified lines (See ICLAC register) | N/A |

## Animals and other research organisms

Policy information about studies involving animals; ARRIVE guidelines recommended for reporting animal research, and Sex and Gender in Research

| | |
|---|---|
| Laboratory animals | C57BL/6J (JAX RRID: IMSR 000664), C57BL/6-cPla2a+/- (Provided by Dr. Joseph Bonventre DOI: 10.1038/37635), C57BL/6-SmoK399A/K399A (This paper). Ages of embryos and mice are stated for each experiment. |
| Wild animals | N/A |
| Reporting on sex | Both male and female mice and embryos were included in the study. |
| Field-collected samples | N/A |
| Ethics oversight | Data and materials generated from animals were obtained in accordance with the IACUC-approved St. Jude Children's Research Hospital protocol 608-100616-10/19. All animal husbandry and procedures were performed in accordance with protocols approved by St. Jude Children's Research Hospital (SJCRH). |

Note that full information on the approval of the study protocol must also be provided in the manuscript.

## Plants

| | |
|---|---|
| Seed stocks | N/A |
| Novel plant genotypes | N/A |
| Authentication | N/A |

## Magnetic resonance imaging

### Experimental design

| | |
|---|---|
| Design type | Experiment was resting state. |

| | |
|---|---|
| Design specifications | N/A |
| Behavioral performance measures | N/A |

## Acquisition

| | |
|---|---|
| Imaging type(s) | Structural |
| Field strength | 72-mm<br>transmit/receive coil. |
| Sequence & imaging parameters | The imaging protocol included T2-weighted turbo spin echo sequences in horizontal (TR/TE = 3080/40 ms, matrix size = 256 x 256, field of view = 36 mm x 36 mm, slice thickness = 0.5 mm, number of slices = 20), coronal (TR/TE = 4760/42 ms, matrix size = 192 x 192, field of view = 15 mm x 15 mm, slice thickness = 0.5 mm, number of slices = 30), and sagittal (TR/TE = 3580/39 ms, matrix size = 128 x 256, field of view = 16 mm x 32 mm, slice thickness = 0.5 mm, number of slices = 24) orientations. |
| Area of acquisition | whole brain scan |

Diffusion MRI ☐ Used ☒ Not used

## Preprocessing

| | |
|---|---|
| Preprocessing software | MRI image analysis was performed using 3D Slicer (Surgical Planning Laboratory). Coronal images were segmented by manually outlining regions of interest to delineate the entire brain. This segmentation process was repeated across all slices to cover the entire brain volume. The brain volume was then calculated based on voxel dimensions. Similarly, a separate segmentation was created for the cerebellum, and its volume was determined using the same method. |
| Normalization | Data were normalized based on total brain volume. |
| Normalization template | Data were normalized based on total brain volume. |
| Noise and artifact removal | N/A |
| Volume censoring | N/A |

## Statistical modeling & inference

| | |
|---|---|
| Model type and settings | Two-tailed Student's t-test |
| Effect(s) tested | Dimensions of brain regions. |

Specify type of analysis: ☒ Whole brain ☐ ROI-based ☐ Both

| | |
|---|---|
| Statistic type for inference<br>(See Eklund et al. 2016) | N/A |
| Correction | N/A |

## Models & analysis

| n/a | Involved in the study |
|---|---|
| ☒ | ☐ Functional and/or effective connectivity |
| ☒ | ☐ Graph analysis |
| ☒ | ☐ Multivariate modeling or predictive analysis |
